# Comprehensive and Combined Omics Analysis Reveals Immune–Infiltration‐Associated Alternative Splicing and RBP Regulators Contributing to Ischemia–Reperfusion Injury in Liver Transplantation

**DOI:** 10.1155/ijog/4835678

**Published:** 2026-09-21

**Authors:** Bincheng Gui, Hanfei Huang

**Affiliations:** ^1^ Department of Organ Transplantation, The First Affiliated Hospital of Kunming Medical University, Kunming, Yunnan, China, kmmc.cn

**Keywords:** alternative splicing, immune microenvironment, ischemia–reperfusion injury, liver transplantation, RNA-binding proteins

## Abstract

**Background:**

Hepatic ischemia–reperfusion injury (HIRI) is a major cause of early allograft dysfunction after liver transplantation, but its post‐transcriptional regulation remains poorly understood. This study aimed to characterize alternative splicing (AS) events in HIRI and explore a potential association network involving RNA‐binding proteins (RBPs), AS, and immune responses.

**Methods:**

Using the GSE151648 dataset (80 paired samples from 40 patients), we identified regulated alternative splicing (RAS) events and analyzed dynamic changes in immune cell populations. An RBP‐RAS co‐expression network was constructed, and single‐cell data (GSE171539, one donor, three time points) were used to explore cell‐type‐specific expression of key RBPs in a qualitative, hypothesis‐generating capacity. RBP expression changes were validated in an independent microarray cohort (GSE12720, 20 paired biopsies) and cross‐validated with Human Protein Atlas liver proteomic data.

**Results:**

Mild IRI (IRI−) samples mainly exhibited alternative 5 ^′^/3 ^′^ splice site events, whereas severe IRI (IRI+) samples were characterized by intron retention and overlapping exons, suggesting that severe injury may involve spliceosomal dysfunction. Post‐transplantation, activated mast cells and NK cells increased, with a shift toward M0 macrophages and a decrease in M2 macrophages. Thirty immune‐related RAS events significantly correlated with immune cell proportions (|*r*| ≥ 0.5, *p* < 0.01). An RBP‐RAS network comprising 43 differentially expressed RBPs was constructed, showing coordinated co‐variation between splicing factors and immune‐associated splicing events. Single‐cell analysis further revealed cell‐type‐specific expression patterns of multiple RBPs across hepatocytes, endothelial cells, and immune populations. External validation in GSE12720 confirmed that 39 of 42 detected RBPs (93%) were consistently upregulated post‐reperfusion, and the Human Protein Atlas confirmed protein‐level expression for 25 of 43 RBPs (58%) in normal liver tissue.

**Conclusions:**

This study suggests that mild and severe HIRI are associated with distinct splicing programs and that these changes are closely associated with immune microenvironment remodeling, supporting the hypothesis of an RBP‐AS‐immune regulatory network. These findings, in which the RBP expression changes were independently validated in an external cohort, provide a basis for generating hypotheses regarding post‐transcriptional regulation in HIRI.

## 1. Introduction

End‐stage liver disease has become a major cause of patient mortality in recent years [1]. For most patients, liver transplantation remains the only curative option, but it is frequently complicated by hepatic ischemia–reperfusion injury (HIRI) [2]. HIRI is a primary contributor to early allograft dysfunction (EAD) and acute rejection [3]. The HIRI process triggers an inflammatory storm and cell death, potentially progressing to systemic inflammatory response syndrome (SIRS) [4]. Statistics indicate that approximately 20%–44% of liver transplant recipients experience EAD, significantly increasing the risk of graft failure and patient mortality [5]. Therefore, investigating the mechanisms and interventional targets of HIRI is of great importance.

Traditional studies have focused on transcriptional regulation in HIRI, implicating pathways such as hypoxia‐inducible factor 1‐alpha (HIF‐1*α*) and nuclear factor kappa‐B (NF‐*κ*B) in the inflammatory storm [6]. However, these pathways cannot explain a critical observation: why do different cell types—or even the same cell type at different time points—exhibit distinct functional phenotypes under identical ischemic stimuli? HIRI is characterized by dynamic, cell‐type‐specific responses [7], suggesting that transcriptional regulation alone cannot fully capture its complexity. This points to the need for post‐transcriptional layers of control.

Alternative splicing (AS) generates transcript diversity from a single gene and is involved in various liver diseases [8–12], but its role in HIRI remains poorly understood [13]. Recent studies have identified numerous differentially alternative spliced (DAS) genes between HIRI and sham‐operated groups, which are significantly enriched in pathways related to inflammation, oxidative stress, apoptosis, and mitochondrial dysfunction [14]. These findings indicate that AS may not be merely a concomitant phenomenon but could actively contribute to HIRI pathology. Therefore, elucidating the role of AS in HIRI is crucial for developing therapeutic strategies.

The precise regulation of AS is primarily achieved by the spliceosome [15]. RNA‐binding proteins (RBPs), as core components and regulators of the spliceosome, modulate exon inclusion or exclusion by recognizing cis‐acting elements on pre‐mRNA [16], highlighting their essential regulatory function in AS. Recent studies integrating transcriptomics and machine learning have screened 1411 candidate RBPs and identified three diagnostic markers—BTG2, CCNL1, and DNAJB1—which are primarily expressed in hepatic macrophages and T cells, with expression levels closely correlated with TNF‐*α* signaling and inflammasome activation [16]. Single‐cell sequencing analysis revealed that these RBPs are primarily expressed in hepatic macrophages and T cells, with their expression levels closely correlated with TNF‐*α* signaling pathway activation and inflammasome activation. This suggests a possible link between RBP‐mediated splicing regulation and inflammatory responses during HIRI. Despite these advances, critical gaps remain. The specific splicing factors involved in HIRI are largely unknown, the molecular mechanisms by which RBPs sense ischemic/hypoxic signals and regulate downstream targets require elucidation, and interventional strategies targeting the RBP–AS axis are lacking.

To address these gaps, we hypothesized that widespread AS alterations occur during hepatic ischemia–reperfusion and correlate with HIRI severity—with mild and severe IRI associated with distinct splicing programs—and that these changes are closely linked to immune microenvironment remodeling. Using public transcriptomic data (GSE151648, GSE171539, GSE12720), we systematically characterized RAS events, immune cell dynamics, and RBP‐AS co‐expression patterns, and evaluated the diagnostic potential of candidate RBPs. This study is computational and exploratory in nature; all findings are intended to be hypothesis‐generating and require experimental validation.

## 2. Materials and Methods

### 2.1. Data Source

Public sequence data files from GSE151648 were downloaded from the Sequence Read Archive (SRA). SRA Run files were converted to fastq format with NCBI SRA Tool fastq‐dump. The raw reads were trimmed of low‐quality bases using a FASTX‐Toolkit (v.0.0.13; http://hannonlab.cshl.edu/fastx_toolkit/). Then, the clean reads were evaluated using FastQC (http://www.bioinformatics.babraham.ac.uk/projects/fastqc). Across all 80 samples, SRA metadata (SRP265616) recorded a mean of 35.95 million reads per sample (range: 28.78–45.79 million; SE 49 bp). Gene detection was comparable across groups (mean: 23,155; range: 18,616–24,933; Table S13). IRI severity classification was based on the original study’s published histopathological scoring system [17]. Briefly, post‐reperfusion liver biopsies obtained 2 h after portal vein unclamping were assessed for neutrophilic inflammatory infiltration and hepatocyte necrosis. Patients with prominent neutrophilic infiltrates and necrotic hepatocytes were classified as severe IRI (IRI+, *n* = 23), whereas those with minimal or absent infiltration and necrosis were classified as mild IRI (IRI−, *n* = 17). All 80 samples were collected from a single‐center prospective cohort and sequenced together in a single batch [18], minimizing potential batch confounding at the data generation stage. Clinical characteristics of both cohorts are summarized in Table S9.

### 2.2. Differential Expression Analysis

HISAT2 [19] was utilized to align high‐quality reads to the human GRCh38 genome reference. Uniquely mapped reads were subsequently used to calculate fragments per kilobase of exon per million mapped fragments (FPKM) for each gene. Differential gene expression analysis was performed using the DESeq2 software package [20]. All 80 samples were re‐processed uniformly from raw FASTQ files through an identical bioinformatics pipeline comprising adapter trimming, HISAT2 alignment to the GRCh38 reference genome, and transcript quantification. Differential expression analysis was performed using DESeq2 with a paired design (~ patient + condition) to account for the paired pre‐ and post‐transplant structure of the data. DESeq2 models raw read counts, employing scaling factors to account for differences in library depth. Gene‐wise dispersion estimates were calculated and subsequently shrunk to generate more accurate dispersion estimates for modeling read counts. Finally, a negative binomial distribution model was fitted using DESeq2, and hypothesis testing was conducted using the Wald test or likelihood ratio test. DESeq2 facilitates differential expression analysis between two or more samples. Results were evaluated based on fold change (FC) and false discovery rate (FDR). The threshold for differential expression was set at |FC| ≥ 2 or ≤ 0.5, with FDR < 0.05. Differential expression significance was determined using Benjamini–Hochberg adjusted *p* values (*p*adj < 0.05). The FPKM expression matrix of 43 DE RBPs is provided in Table S3.

### 2.3. Analysis of AS Events

The AS events and regulated alternative splicing (RAS) events among different groups were defined and quantified by using the SUVA pipeline as described previously. Reads proportion of SUVA AS event (pSAR) of each AS events were calculated [21]. The frequency threshold (≥ 0.2) was selected to retain RAS events detected in at least 20% of patient‐paired comparisons within each IRI group, ensuring recurrence across patients while preserving discovery sensitivity. The pSAR threshold (≥ 50%) was applied to restrict analyses to major transcript isoforms, which are more likely to have functional consequences than minor isoforms. Both thresholds are consistent with prior SUVA‐based studies. These thresholds were fixed a priori rather than tuned to the present data. Sensitivity analyses evaluating alternative choices for both thresholds are provided in Table S10. The SUVA pipeline compares pre‐ and post‐transplant samples within each patient to identify RAS events [21]. Because each patient serves as their own biological control, potential confounding by inter‐individual variability or technical batch effects is inherently controlled in this paired analytical framework. All identified RAS events with SUVA statistics are provided in Table S1 and the pSAR matrix of high‐confidence RAS events is provided in Table S2.

### 2.4. Cell Type Quantification

The CIBERSORT algorithm [22] (version 1.03) was utilized with default parameters to estimate the proportions of immune cell types based on gene expression FPKM values. A total of 22 human immune cell phenotypes were analyzed, including five T cell types (CD8 T cells, resting memory CD4 T cells, activated memory CD4 T cells, follicular helper T cells, and regulatory T cells [Tregs]), naive and memory B cells, plasma cells, resting and activated NK cells, monocytes, macrophages (M0, M1, M2), resting and activated dendritic cells, resting and activated mast cells, eosinophils, and neutrophils. CIBERSORT‐estimated immune cell fractions for all 80 samples are provided in Table S4.

### 2.5. Co‐Expression Analysis

A catalog of 2142 human RBPs was compiled by merging data from four previous reports [23–26]. The co‐disturbed network between the expression of RBPs and splicing ratio of RAS events (pSAR ≥ 50*%*) was constructed using 80 samples from GSE151648. We calculated the Pearson’s correlation coefficients (PCCs) between them and classified their relation into three classes: positive correlated, negative correlated, and noncorrelated based on the PCCs value. Two distinct correlation thresholds were applied to reflect the different goals of each network layer. For RBP‐RAS associations, |r| ≥ 0.6 was used to ensure that only strong‐effect correlations entered network construction. For RAS‐immune cell associations, |r| ≥ 0.5 was used to preserve sufficient network connectivity for visualization and biological interpretation. Both thresholds were combined with a nominal *p* ≤ 0.01. By Cohen’s convention, |r| ≥ 0.6 and |r| ≥ 0.5 represent strong and moderate‐to‐strong effect sizes, respectively. Sensitivity analyses across alternative correlation thresholds are provided in Table S10; at *n* = 80, these thresholds are more stringent than Bonferroni‐corrected significance, providing an implicit and conservative multiple‐testing control. To further assess whether the retained edges could arise by chance, we performed a permutation test in which sample labels were randomly shuffled 1000 times and all RBP–RAS correlations were recomputed; under the null, no RBP–RAS pair reached the |r| ≥ 0.6 threshold (empirical *p* < 0.001). In addition, a bootstrap stability analysis (500 resampling replicates) confirmed that 100% of retained edges were recovered in ≥50*%* of replicates (mean edge stability 81.8%; Table S18). RBP‐RAS co‐expression network edges are listed in Table S5.

### 2.6. Single‐Cell RNA Sequencing Data Preprocessing and Analysis

Single‐cell RNA‐seq data from liver tissues collected before ischemia, at the end of cold ischemia preservation, and 2 h post‐reperfusion during liver transplantation were obtained. The unique molecular identifier (UMI) count matrix was downloaded from the GSE171539 dataset. The R package Seurat [27] (version 4.0.4) was used to convert the UMI count matrix into a Seurat object. For preprocessing, low‐quality cells were filtered out based on the following criteria: cells with UMI counts < 1000, detected genes < 500, or mitochondrial‐derived UMI count proportion > 15%. Genes detected in fewer than three cells were also excluded from downstream analyses. Following quality control, the UMI count matrix underwent log‐normalization. The top 2000 variable genes were selected, followed by principal component analysis (PCA). Cells were clustered using the Louvain algorithm and visualized with UMAP. For single‐cell marker gene identification, the FindMarkers function in Seurat applies Benjamini–Hochberg correction by default, with significance defined as *p* val adj < 0.05.

### 2.7. Functional Enrichment Analysis

To sort out functional categories of DEGs, Gene Ontology (GO) terms and KEGG pathways were identified using KOBAS 2.0 server [28]. Hypergeometric test and Benjamini–Hochberg FDR controlling procedure were used to define the enrichment of each term. Reactome (http://reactome.org) pathway profiling was also used for functional enrichment analysis of the sets of selected genes.

### 2.8. Other Statistical Analyses

PCA analysis was performed by R package factoextra (https://http://cloud.r-project.org/package=factoextra) to show the clustering of samples with the first two components. After normalizing the reads by TPM (Tags Per Million) of each gene in samples, in‐house custom scripts were used for visualization of next‐generation sequence data and genomic annotations. The pheatmap package (https://cran.r-project.org/web/packages/pheatmap/index.html) in R was used to perform the clustering based on Euclidean distance. Student’s *t*‐test was used for comparisons between two groups. For multiple hypothesis testing, the Benjamini–Hochberg FDR procedure was applied in the following analyses: differential expression (DESeq2, *p*adj < 0.05), functional enrichment (KOBAS 2.0, FDR < 0.05), and single‐cell marker gene identification (Seurat FindMarkers, *p*_val_adj < 0.05). For co‐expression network construction (RBP‐RAS and RAS‐immune correlations), we used a stringent nominal *p* value threshold (*p* ≤ 0.01) in combination with a biological effect size filter (|Pearson^‘^s r| ≥ 0.6 for RBP‐RAS; |r| ≥ 0.5 for RAS‐immune), consistent with the exploratory, hypothesis‐generating nature of this network analysis. For external validation in GSE12720, the Benjamini–Hochberg procedure was applied (limma, FDR < 0.05).

### 2.9. External Validation

The GSE12720 dataset (20 paired pre‐ischemia vs. post‐reperfusion liver) transplant biopsies, GPL570 platform) was used for independent validation. CEL files were RMA‐normalized and mapped to gene symbols using hgu133plus2.db. Paired differential expression was performed with limma (Benjamini–Hochberg FDR < 0.05). For protein‐level validation, the 43 RBPs were cross‐referenced with Human Protein Atlas (v23) liver immunohistochemistry (IHC) data.

### 2.10. In Silico Perturbation Analysis

Two orthogonal in silico perturbation analyses were performed to evaluate the robustness of the RBP–AS–immune network beyond pairwise correlation. All analyses were conducted using custom scripts in R (version 4.5.3).

A tripartite network was constructed comprising 43 differentially expressed RBPs connected to 22 immune‐associated RAS events (edges defined as |Pearsons r| ≥ 0.6, *p* ≤ 0.01, based on the co‐expression analysis in Section 2.5), with RAS events further connected to 18 immune cell types (edges defined as |Pearsons r| ≥ 0.5, *p* ≤ 0.01, based on the RAS–immune correlation analysis in Section 3.4). Network topology metrics were calculated using the igraph package (version 1.6.0) [29]. For each RBP, we computed the following: (i) network degree (number of directly connected RAS events); (ii) immune impact score, defined as the cumulative absolute correlation coefficient of connected RAS events with immune cell types; (iii) bottleneck centrality, defined as *Σ*(1/*d* *e* *g* *r* *e* *e*_*r* *a* *s*_), where *d* *e* *g* *r* *e* *e*_*r* *a* *s*_ is the total number of RBPs connected to a given RAS event, giving greater weight to RAS events with few alternative RBP connections; and (iv) a composite criticality score = degree × immune impact × bottleneck. Each RBP was systematically removed from the network using the delete_vertices function, and the number of RAS events losing their strongest connector or becoming entirely disconnected was recorded.

For each of the 43 network RBPs and four HIRI‐relevant immune cell types (mast cells activated, NK cells activated, M0 macrophages, M2 macrophages)—selected a priori based on their prominence in the CIBERSORT analysis (Section 3.3)—samples from GSE151648 (*n* = 80) were stratified into RBP‐high (top quartile of FPKM) and RBP‐low (bottom quartile) groups. The FPKM expression matrix (Table S3) and CIBERSORT‐estimated immune cell fractions (Table S4) were used as input. Effect sizes were quantified by Cohen’s d, calculated as (mean_high − mean_low)/pooled standard deviation, and statistical significance was assessed by two‐sided Mann–Whitney *U* test (wilcox.test). Multiple testing correction was applied across all 172 RBP–cell pairs using the Benjamini–Hochberg FDR procedure (*p*.adjust, method = “BH”). Significance was defined as FDR < 0.05.

To test whether individual RBP–immune cell associations were independent of broader transcriptional covariation among RBPs, partial Spearman correlation was performed using the ppcor package (version 1.1) [29] in R. For each RBP–immune cell pair, the Spearman correlation was computed while controlling for the FPKM expression values of all other 42 network RBPs as covariates (pcor.test, method = “spearman”). Multiple testing correction (Benjamini–Hochberg, FDR < 0.05) was applied across all 172 tested pairs. The complete analytical pipeline pseudocode is provided in Text S1

## 3. Results

### 3.1. Identification of Highly Conserved RAS Events Before and After Liver Transplantation

**Figure 1:**
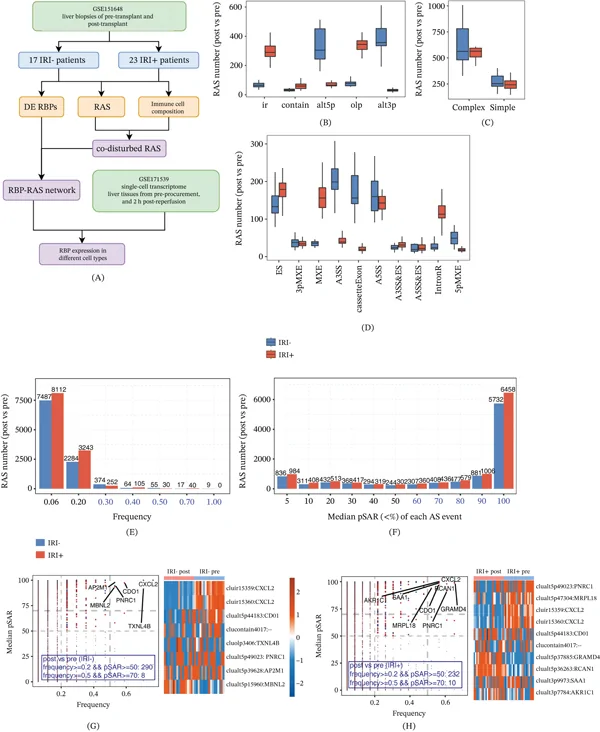
Identification of highly conserved regulated AS events (RAS) between samples before and after liver transplantation. (A) Workflow of bioinformatic analysis in this study. (B) Boxplot showing number of RAS detected by SUVA across paired liver biopsies samples of pre‐transplant and post‐transplant from 17 IRI− patients and 23 IRI+ patients, respectively. (C) Boxplot showing number of SUVA RAS events, which contains SJs involved in two or more different classical splicing events (complex) or in same classical splicing event (simple). (D) Splice junction constituting RAS events detected by SUVA was annotated to classical AS event types. The number of each classical AS event types were showed with boxplot. (E) Bar plot showing RAS frequency across 17 IRI− and 23 IRI+ patients with paired samples of pre‐transplant and post‐transplant, respectively. X axis indicates percentage of samples which detected RAS. Y axis indicates RAS number with different frequency. (F) Bar plot showing RAS number with different abundance (pSAR) of all detected RAS. (G) Scatter plot in left panel showing RAS from IRI‐ group with different frequency and pSAR. RAS which frequency ≥ 0.5 and pSAR ≥ 70*%* were labeled. Heatmap in right panel showing splicing ratio across all IRI‐ samples for RAS, which frequency ≥ 0.5 and pSAR ≥ 70*%* and corresponding genes. (H) Scatter plot in left panel showing RAS from IRI+ group with different frequency and pSAR. RAS which frequency ≥ 0.5 and pSAR ≥ 70*%* were labeled. Heatmap in right panel showing splicing ratio across all IRI+ samples for RAS, which frequency ≥ 0.5 and pSAR ≥ 70*%* and corresponding genes.

RNA‐seq data from 80 samples, including paired pre‐ and post‐transplant liver biopsies from 40 patients, were obtained from GSE151648. Using the SUVA software, differential AS events before and after transplantation were identified and analyzed separately in the IRI− (mild) and IRI+ (severe) patient groups. Frequently occurring splicing events (frequency ≥ 0.2) that constituted the major transcript isoform (pSAR ≥ 50*%*) were used for subsequent construction of the RBP–RAS regulatory network. The most highly conserved splicing events (frequency ≥ 0.5 and pSAR ≥ 70*%*) and their corresponding host genes were also displayed. These AS events, as critical post‐transcriptional regulatory mechanisms, may participate in the remodeling of gene expression regulatory networks following ischemia–reperfusion and serve as potential biomarkers and therapeutic targets for HIRI. Figure 1A presents the study workflow. Five types of RAS events were identified. The IRI− group was primarily characterized by alt5p and alt3p events, whereas the IRI+ group was predominantly associated with ir and olp events (Figure 1B).

Our analysis revealed that approximately two‐thirds of RAS events were complex splicing events, highlighting the complexity of splicing regulation following hepatic ischemia–reperfusion (Figure 1C). Mapping the SUVA‐identified splicing events to canonical AS types showed that, compared with the IRI+ group, the IRI− group exhibited a higher detection of differential AS events, such as alternative 3 ^′^ splice sites (A3SS), cassette exons, and mutually exclusive 5 ^′^ UTRs (5pMXE), while showing lower detection of mutually exclusive exons (MXE) and intron retention (IntronR) events (Figure 1D). A bar chart illustrated the detection frequency of RAS events among patients in the IRI− and IRI+ groups, with higher frequency indicating greater conservation (Figure 1E). RAS events with a frequency ≥ 0.2 were used in subsequent analyses. However, some splicing events represent minor transcripts. pSAR measures the proportion of the transcript isoform containing a specific splicing event relative to the total transcriptional level of the gene. For subsequent analyses, RAS events with pSAR ≥ 50*%* were utilized (Figure 1F).

Scatter plots and heat maps were used to display the most highly conserved differential splicing events (frequency ≥ 0.5 and pSAR ≥ 70*%*) before and after reperfusion in HIRI. These highly conserved splicing events may serve as potential biomarkers and therapeutic targets for HIRI. Additional PCA and heat map analyses of RAS events are provided in Figure S1.

### 3.2. Comparison of RAS Events Before and After Liver Transplantation in Patients With Different IRI Severities

**Figure 2:**
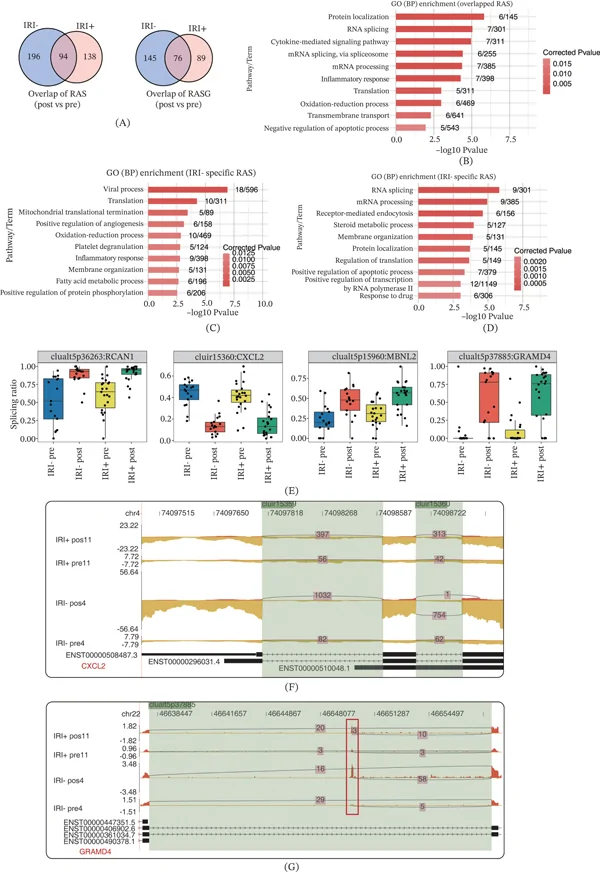
Comparison of RAS between samples before and after liver transplantation with different IRI severity. (A) Venn diagram in left panel showing the overlap of RAS (frequency ≥ 0.5 and pSASR ≥ 70*%*) detected from IRI− and IRI+ samples. Venn diagram in right panel showing the overlap of RAS host genes (RASG). B. GO biological process enrichment for host genes of overlapped RAS of IRI− and IRI+. Bars represent −log10 (corrected *p* value, Benjamini–Hochberg FDR). (C) GO biological process enrichment for host genes of IRI‐ specific RAS. Bars represent −log10 (Corrected *p* value, Benjamini–Hochberg FDR). (D) GO biological process enrichment for host genes of IRI+ specific RAS. Bars represent −log10 (corrected *p* Value, Benjamini–Hochberg FDR). (E) Boxplot showing splicing ratio profile across 80 samples of four highly conserved splicing events. Splicing events model was showed in right panel. (F) Visualization of two IR RAS events from CXCL2 in samples from different groups. Splice junctions were labelled with SJ reads number. (G) Visualization of an alt5p event from GRAMD4 in samples from different groups. Splice junctions were labelled with SJ reads number. Altered exon was marked out with red box.

To compare the differences in AS events before and after transplantation across varying severities of ischemia–reperfusion injury, we performed overlap analyses on the differential splicing events identified in the IRI− and IRI+ groups, as well as on the host genes of these events (Figure 2A). This analysis identified RAS events that were commonly and significantly altered before and after transplantation in both the IRI− and IRI+ groups. Subsequently, GO enrichment analysis was conducted on the host genes of group‐specific RAS events (Figure 2B). Genes hosting IRI− specific splicing events were primarily enriched in pathways related to viral processing, translation, angiogenesis, inflammatory response, oxidation–reduction process, and fatty acid metabolism (Figure 2C). In contrast, genes hosting IRI+ specific splicing events were mainly enriched in pathways including RNA splicing, steroid metabolism, and apoptosis (Figure 2D). The most highly conserved overlapping splicing events between the IRI− and IRI+ groups were also displayed (Figure 2E), with boxplots showing the splicing ratio (pSAR) for each event. These results indicate that, although differences exist between IRI− and IRI+, the direction of change for the most highly conserved splicing events before and after IRI is consistent.

Figure 2F illustrates two intron retention events in the CXCL2 gene. Post‐transplantation, although the overall expression of CXCL2 increased, the proportion of the transcript isoform retaining the intron decreased substantially. Figure 2G depicts an alternative 5 ^′^ splice site event in the GRAMD4 gene. Post‐transplantation, this gene showed a preference for a proximal 5 ^′^ splice site, indicating a shift in splicing pattern. These findings suggest that HIRI actively regulates AS events, and this regulation is closely associated with the severity of IRI. KEGG pathway enrichment analysis of RAS host genes is shown in Figure S2.

### 3.3. Differences in Immune Microenvironment Characteristics Between IRI− and IRI+ Samples Before and After Liver Transplantation

**Figure 3:**
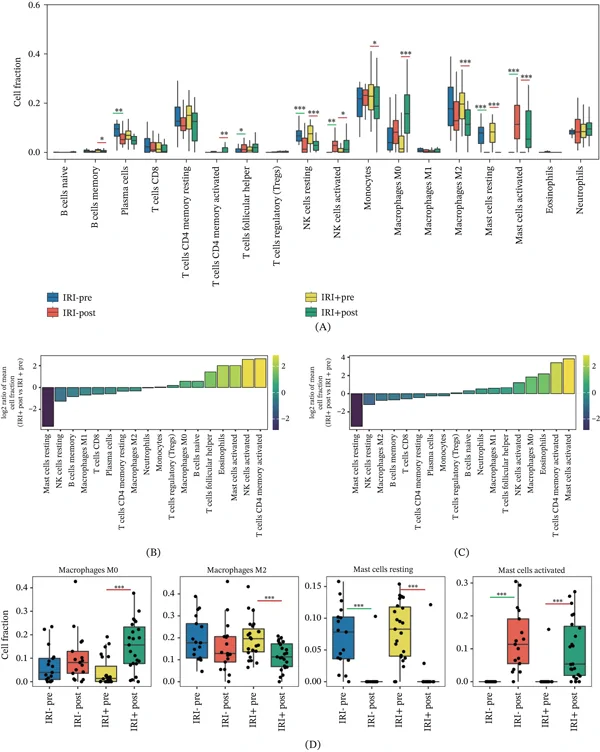
Diversity of immune microenvironment characteristics between samples before and after liver transplantation underlying IRI‐ or IRI+. (A) Boxplot showing fraction of each cell type in each group. The significant difference of cell fractions between Pre and Post samples was calculated using the Student’s t test. ∗: *p* value ≤ 0.05, ∗∗: *p* value ≤ 0.01, ∗∗∗: *p* value ≤ 0.001. (B) IRI− POST relative to PRE group rank ordered based on decreasing values of the relative frequency ratio at POST versus PRE. (C) IRI+ POST relative to PRE group rank ordered based on decreasing values of the relative frequency ratio at POST versus PRE. (D) Boxplot showing cell fraction of four cell types.

To investigate differences in the immune microenvironment between the IRI− and IRI+ groups, we applied the CIBERSORT algorithm to deconvolute the RNA‐seq data, estimating the relative proportions of 22 immune cell types across all 80 pre‐ and post‐transplant samples. Significant changes were observed in the proportions of specific cell populations. In post‐transplant samples, the proportion of resting mast cells decreased significantly, whereas that of activated mast cells increased. Mast cells are multifunctional immune cells involved in characteristic symptoms (e.g., pruritus, ocular swelling, rhinorrhea), immediate hypersensitivity, and chronic allergic responses. A similar activation trend was observed for NK cells (Figure 3A). Furthermore, compared with the IRI− group, the IRI+ group exhibited a more marked increase in M0 macrophages and a more substantial decrease in M2 macrophages. This suggests that shifts in macrophage polarization states are strongly correlated with the severity of ischemia–reperfusion injury. In Figure 3A, cell types marked with asterisks indicate significant differences between post‐ and pre‐transplant groups, as determined by separate *t*‐tests for IRI− and IRI+ patients (∗*p* ≤ 0.05; ∗∗*p* ≤ 0.01; ∗∗∗*p* < 0.001). Comparison of post‐/pre‐transplant ratios for different cell types revealed significant changes specifically in mast cells and NK cells (Figures 3B,C). Boxplots in Figure 3D further illustrate the proportional changes of four selected cell types before and after transplantation. Complete immune cell fraction profiles and additional cell type comparisons are presented in Figure S3. We note that CIBERSORT provides estimated rather than directly measured cell proportions; therefore, comparisons focus on relative differences between groups. Reassuringly, the key immune alterations identified in our analysis—mast cell activation and M0/M2 macrophage imbalance—are consistent with prior experimental studies of hepatic IRI that employed orthogonal methods. Mast cell degranulation has been demonstrated by toluidine blue staining and electron microscopy in a rat model of partial hepatic ischemia [30], and M0/M2 macrophage polarization imbalance has been confirmed by immunohistochemical CD68/CD163/CD206 staining in human liver transplantation biopsies [31]. These experimental findings provide independent support for the directionality of the CIBERSORT‐inferred trends.

### 3.4. Dynamic Changes of AS Events Associated With Immune Microenvironment Regulation in Liver Transplant Patients With Different IRI Severities

Correlation analysis was performed between the previously identified RAS events and immune cell proportions. A total of 30 RAS events were found to be significantly correlated with changes in immune cell proportions (|PCC| ≥ 0.5 with at least one cell type; *p* ≤ 0.01). GO enrichment analysis of the host genes of these 30 RAS events revealed significant enrichment in immune and inflammatory pathways, suggesting that changes in the immune microenvironment before and after liver transplantation may be influenced by the AS of these genes.

**Figure 4:**
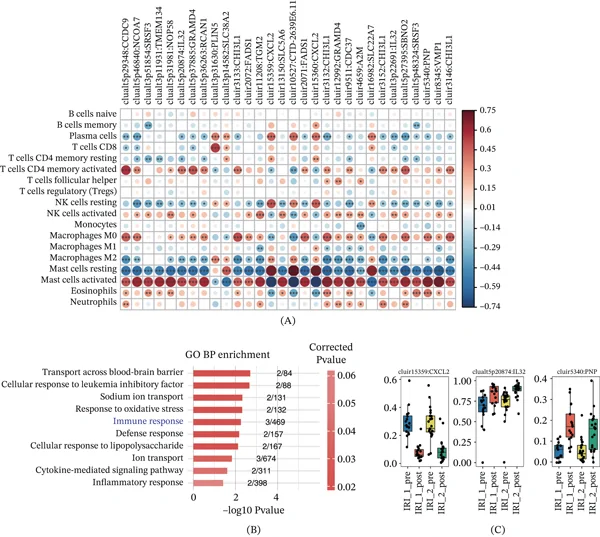
Dynamic changes of alternative splicing events associated with immune microenvironment regulation in samples before and after liver transplantation patients with varying IRI degrees. (A) Co‐expression analysis of RAS with at least one immune cell with *p* value ≤ 0.01 and Pearson^’^s correlation value ≥ 0.5 or ≤−0.5. (B) Bar plot exhibits the most enriched GO biological process results of co‐expressed RAS host genes with immune cells. −log10 (corrected *p* Value, Benjamini–Hochberg FDR) is shown for each enriched GO term. (C) Boxplot showing splicing ratio profile of three splicing events involved in immune response term from Figure 4B.

Figure 4A displays RAS events that significantly co‐vary with immune cell proportions (union of the IRI+ and IRI− groups). Color intensity indicates correlation strength (red: positive correlation; blue: negative correlation), and asterisks denote significance levels (∗*p* ≤ 0.05; ∗∗*p* ≤ 0.01; ∗∗∗*p* < 0.001). Each RAS event showed a correlation with *p* ≤ 0.01 for at least one immune cell type.

Figure 4B presents the enriched pathways for the host genes of RAS events significantly correlated with immune cell changes (from Figure 4A), primarily involving immune inflammation and ion transport pathways. Boxplots in Figure 4C illustrate the changes in splicing ratios before and after transplantation for three RAS events enriched in immune response pathways (selected from Figure 4B). The splicing ratio profiles of the 30 RAS events that co‐vary with immune cells are displayed in Figure S4. RAS–immune cell correlation statistics are provided in Table S6. The 30 immune‐associated RAS events with host genes, splicing types, and full correlation coefficients are provided in Table S7.

### 3.5. Construction of an RBP and Immune Infiltration‐Related RAS Co‐Variation Network

**Figure 5:**
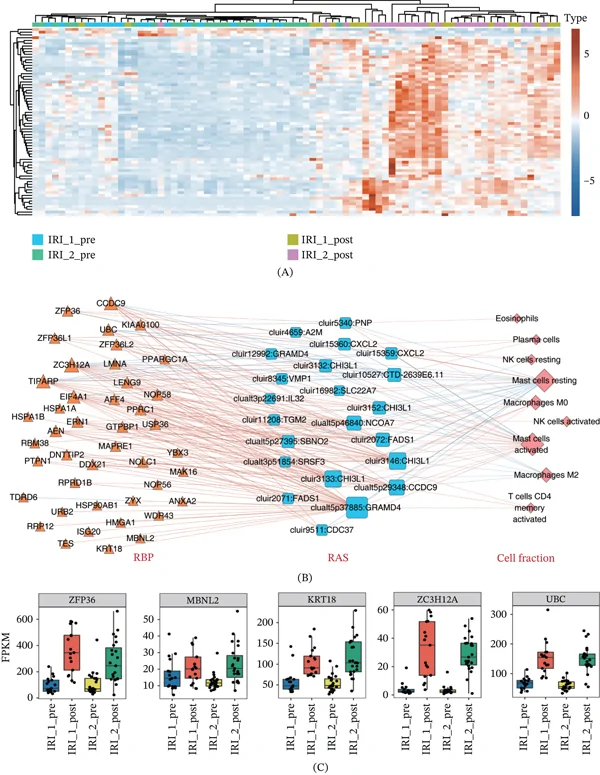
Construction of co‐disturbed network between RNA‐binding proteins (RBP) and immune‐infiltration‐associated RAS. (A) Expression heat map of all significant differentially expressed (DE) RBPs between samples before and after liver transplantation underlying IRI− or IRI+. (B) The co‐disturbed network among expression of DE RBPs and splicing ratio of RAS events (fre ≥ 0.2 and pSAR ≥ 50*%*) was constructed using 80 samples. |Pearson^’^s correlation| ≥ 0.6 and *p* value ≤ 0.01 were retained for RBP and RAS correlation. |Pearson^’^s correlation| ≥ 0.5 and *p* value ≤ 0.01 were retained for RAS and immune‐cell correlation. Triangles in left represent RBP genes. Squares in left indicate RAS. Rhombus in right indicate co‐disturbed immune cells of RAS. Node size indicates the degree of connection with other nodes. (C) Boxplot showing expression profile of five example RBPs regulators.

Differential expression analysis was performed comparing pre‐ and post‐transplant samples (separately for IRI− and IRI+ groups, taking the union of differentially expressed genes). Using |fold change| ≥ 2 and FDR ≤ 0.05 as thresholds, significantly differentially expressed RNA‐binding protein genes (DE RBPs) were identified. Most DE RBPs were up‐regulated, indicating widespread upregulation of RBPs following HIRI (Figure 5A). An RBP–RAS co‐variation regulatory network was constructed by correlating these RBPs with the 30 previously identified RAS events. This network revealed 43 RBPs that co‐varied with the RAS events (Figure 5B). These RBPs may be associated with IRI severity through their correlation with RAS events. Five RBPs of particular interest were identified, and their expression levels before and after transplantation were examined. Increased expression post‐transplantation was observed in both IRI− and IRI+ groups for these genes (Figure 5C). Expression profiles of additional RBPs from the co‐expression network are shown in Figure S5. The 43 RBPs with their FCs, network degree, and correlated RAS events are listed in Table S8. Permutation and bootstrap stability analyses confirmed that the observed network edges were highly unlikely to arise by chance (empirical *p* < 0.001; 100% edge recovery rate; Table S18).

### 3.6. Single‐Cell Transcriptomic Analysis Reveals Regulated Expression of RBPs in Heterogeneous Immune Cell Populations Before and After Liver Transplantation

**Figure 6:**
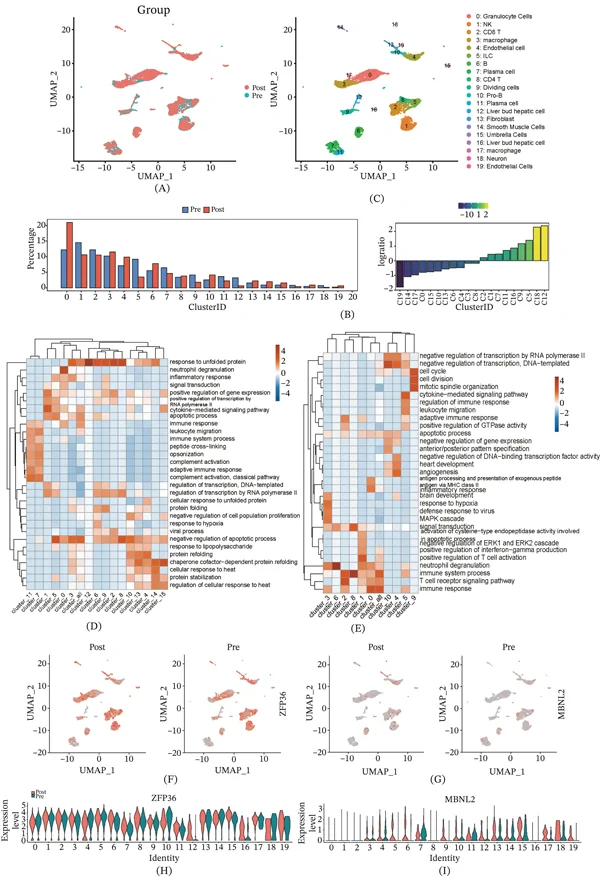
Single‐cell transcriptomic analysis delineates regulated expression of RBPs in heterogeneous immune cell populations in samples before and after liver transplantation. (A) Cellular populations identified. Cell types with cell cluster id for UMAP plot are summarized in the panel right. (B) Percentages of assigned cell types among total cells in each group were calculated. (C) Pre group relative to Post group rank ordered based on decreasing values of the relative frequency ratio at Pre versus Post. (D) GO analysis of up‐regulated genes in each cell type between Post and Pre groups. Top 3 most enriched GO terms (biological process) were selected for plotting heat map. The color indicates significance (‐log10 qvalue) of each term for each cell type. (E) GO analysis of down‐regulated genes in each cell type between Post and Pre groups. Top 3 most enriched GO terms (biological process) were selected for plotting heat map. The color indicates significance (‐log10 qvalue) of each term for each cell type. (F, G) Expression of RBP ZFP36 and MBNL2 were used to label clusters by cell identity as represented in the UMAP plot for Post and Pre groups separately. (H, I) Violin plot showing expression alternation of ZFP36 and MBNL2 between Post and Pre sample in different cell clusters.

To gain deeper insight into the regulated expression of RBPs within immune cell populations before and after liver transplantation, published single‐cell transcriptomic data from liver tissues collected pre‐transplantation and 2 h post‐reperfusion (GSE171539) were downloaded and analyzed. This dataset was derived from a single donor with liver biopsies collected at three longitudinal time points: before ischemia, at the end of cold preservation, and 2 h after reperfusion. We emphasize that given the single‐donor design, this analysis is intended as qualitative, exploratory support for cell‐type‐specific RBP expression patterns and is underpowered for formal statistical comparison between timepoints. Single‐cell data from pre‐transplant and 2 h post‐reperfusion samples were clustered, and UMAP was used to visualize the clustering results. Each cluster was annotated based on established cell type marker genes (Figure 6A). The proportion of each cluster relative to the total cell population was compared between pre‐transplant and post‐reperfusion samples. Clusters with larger proportions included C1 (granulocytes), C2 (NK cells), C3 (CD8+ T lymphocytes), C4 (macrophages), and C5 (endothelial cells) (Figure 6B). Differences in cluster proportions before and after reperfusion were visualized differently by calculating the log2 FC (post‐reperfusion/pre‐transplant). Positive bars indicate clusters increased in pre‐transplant samples, while negative bars indicate clusters increased after reperfusion. The most significantly altered clusters were C12 (hepatocytes), C18 (neuronal cells), C5 (ILC cells), C19 (endothelial cells), C14 (smooth muscle cells), and C17 (macrophages) (Figure 6C). Subsequently, GO enrichment analysis was performed on genes upregulated in each module after reperfusion compared with pre‐transplant. Enriched pathways generally aligned with the annotated cell types. For example, plasma cells were primarily enriched in pathways related to immune inflammation and stress response, consistent with plasma cell function (Figure 6D,E). The expression patterns of two RBPs from Figure 5B, ZFP36 and MBNL2, were visualized at the single‐cell level using UMAP. ZFP36 showed notable differential expression in specific cell types, such as C12 and C16. MBNL2 exhibited differential expression in several cell types, including C5, C6, C9, C12, C17, and C19. This suggests that these RBPs may exert their primary functions within these specific cell populations (Figure 6F–I). Additional single‐cell expression patterns of KRT18 and ZC3H12A are provided in Figure S6.

### 3.7. External Validation of RBP Expression

To independently validate the widespread RBP upregulation observed in GSE151648, we analyzed the GSE12720 microarray dataset (20 paired pre‐ischemia donor vs. post‐reperfusion liver transplant biopsies). Of the 43 RBPs in our co‐expression network, 42 were detected on the array, and 39 (93%) were significantly differentially expressed (FDR < 0.05). Notably, 41 of 42 (98%) were upregulated post‐reperfusion (Figure S8A–C), consistent with the RBP upregulation pattern observed in our primary analysis. Because GSE12720 is a microarray platform, it measures transcript‐level expression through exonic probes and cannot detect splice junction‐spanning reads; accordingly, this external validation pertains to RBP expression rather than to the SUVA‐defined AS events themselves. Key RBPs including ZC3H12A (log2FC = +1.80, FDR = 1.8 × 10^−8^), HSPA1B (+2.52, FDR = 1.7 × 10^−5^), ZFP36 (+1.18, FDR = 3.7 × 10^−5^), and HSP90AB1 (+1.00, FDR = 4.0 × 10^−4^) were all significantly upregulated in this independent cohort.

Protein‐level validation using the Human Protein Atlas (HPA v23) IHC data confirmed that 25 of the 43 RBPs (58%) are expressed at the protein level in normal liver tissue (Figure S7). Of the remaining 18 RBPs, eight (TIPARP, AEN, KIAA0100, ISG20, MBNL2, PPARGC1A, RRP12, and URB2) are not profiled by the HPA IHC atlas, whereas 10 (CCDC9, EIF4A1, PPRC1, ZFP36L2, DNTTIP2, MAPRE1, PTPN1, TDRD6, TES, and ZYX) are profiled but show no detectable staining in normal liver tissue, consistent with their stress‐induced, post‐reperfusion upregulation. Notably, all 18 are annotated as “Evidence at protein level” (PE1) in UniProt (Swiss‐Prot reviewed entries), confirming independent protein‐level evidence (Table S19). KRT18, HSPA1A, and HSP90AB1 showed medium‐to‐high expression in hepatocytes (Enhanced/Supported reliability), while ZFP36, ZC3H12A, and ZFP36L1 showed preferential expression in cholangiocytes (Figure S7B), consistent with their proposed roles in immune and inflammatory regulation. Detailed DE statistics are provided in Table S12 and complete HPA expression data for all 43 RBPs are provided in Table S11.

### 3.8. In Silico Perturbation Analysis Identifies RBPs With High Network Criticality and Immune‐Regulatory Potential

To move beyond pairwise correlation and evaluate the structural robustness of the RBP–AS–immune network, we performed two orthogonal in silico perturbation analyses.

#### 3.8.1. Network In Silico Knockout

Systematic removal of each RBP from the tripartite network revealed that a small subset of RBPs exerts disproportionate structural influence (Figure S9). ZC3H12A (Regnase‐1) emerged as the dominant network hub, with a criticality score of 310.0—2.7‐fold higher than the second‐ranked RBP TIPARP (115.5)—and a network degree of 16 (average = 3.9). ZC3H12A served as the single strongest connector for seven of 22 immune‐associated RAS events. TIPARP (degree = 13) and EIF4A1 (degree = 12) ranked second and third, with criticality scores of 115.5 and 91.3, respectively. Eight of 43 RBPs (19%) were identified as the unique strongest connector for at least one RAS event, indicating that a minority of RBPs contribute disproportionately to network architecture. The network exhibited moderate robustness, as no single RBP removal caused complete collapse; however, the removal of the top three hubs each resulted in substantial fragmentation of RBP–RAS connectivity.

#### 3.8.2. Virtual Expression Perturbation and Partial Correlation

Stratifying samples by RBP expression (top vs. bottom quartile) revealed that all 43 RBPs significantly perturbed at least one HIRI‐relevant immune cell type (FDR < 0.05), with mast cell activation being the most strongly affected phenotype (Figure S9)—consistent with the CIBERSORT deconvolution findings (Figure 3). ZFP36 exhibited the largest perturbation effect on mast cell activation (Cohen^’^s d = 2.77, FDR = 3.4 × 10^−7^), and this association survived the most stringent specificity test: the partial Spearman correlation between ZFP36 and activated mast cells remained significant after controlling for all other 42 network RBPs (partial r = 0.35, FDR = 0.03; Figure S9). ZC3H12A significantly perturbed all four tested immune cell types (all FDR < 0.01), consistent with its network hub status. LENG9 showed a specific association with M0 macrophages (partial r = 0.55, FDR = 2.2 × 10^−5^). ZFP36L1—for which independent experimental validation in human DCD liver grafts has been reported [32]—also showed significant perturbation of two cell types and a significant partial correlation with M0 macrophages (FDR = 0.03), providing external corroboration of the in silico approach.

#### 3.8.3. Multi‐Layer Validation

Across three independent analytical layers (network criticality, expression perturbation, and partial correlation specificity), all 14 RBPs most extensively discussed in this study were validated in at least one layer, and 10 of 14 (71%) in at least two layers (Figure S9). ZFP36 and ZFP36L1 were validated in all three layers. ZC3H12A, the most prominent RBP in our original findings, was validated in two layers (network and perturbation); its absence from the partial correlation layer is biologically expected for a network hub whose expression co‐varies with many other RBPs, making its independent statistical contribution difficult to isolate—a pattern consistent with a central integrative regulatory role.

Overall, the top candidates identified by these orthogonal in silico approaches—ZC3H12A, ZFP36, TIPARP, CCDC9, and ZFP36L1—are precisely the RBPs highlighted in our primary co‐expression network, single‐cell, and external validation analyses. This convergent evidence strengthens the hypothesis that these RBPs are biologically relevant to HIRI. Complete criticality metrics, perturbation statistics, and partial correlation results are provided in Tables S14, S15, S16, and S17.

## 4. Discussion

AS is dynamically regulated during HIRI, but its severity‐dependent patterns and immune links remain unclear. Analyzing 80 paired pre‐/post‐transplant biopsies, we observed that mild HIRI favors alternative splice site usage, whereas severe HIRI was associated with intron retention and overlapping exons—suggesting potential spliceosomal dysfunction. Integrated with immune deconvolution and RBP co‐expression networks, we identify 43 RBPs that co‐vary with immune‐associated AS events, suggesting a potential RBP–AS–immune network that correlates with mast cell activation and M0/M2 macrophage imbalance. These findings may inform future investigations into splicing regulators as therapeutic targets for HIRI.

Research on AS in tissue ischemia–reperfusion injury has largely focused on myocardial IRI, with relatively few studies in hepatic IRI. To date, the most extensively characterized AS event in IRI is CEACAM1 splicing. Under hypoxic conditions, hypoxia‐inducible factor HIF1‐*α* predominantly regulates CEACAM1 AS, which has been shown to limit cellular damage and improve liver function [33]. Specifically, the long isoform of CEACAM1 (CC1‐L) in neutrophils suppresses neutrophil extracellular trap (NET) formation via the S1PR2/S1PR3 axis through an autophagy‐dependent pathway, thereby mitigating IRI injury [33]. These findings suggest CEACAM1 AS may serve not only as a potential biomarker for donor liver quality but also as an important immune‐modulatory factor.

Beyond CEACAM1, our study identified several highly conserved splicing events (frequency ≥ 0.5, pSAR ≥ 70*%*) consistently altered across patients, suggesting they may act as key post‐transcriptional regulators in HIRI. Among these, CXCL2 exhibited two intron retention events. Although total CXCL2 expression increased after transplantation, the proportion of the intron‐retaining isoform markedly decreased. This shift may reflect a potential regulatory mechanism that could affect CXCL2 protein isoform production, a chemokine that recruits neutrophils to the injured liver and promotes IRI [34]. Moreover, the CXCL2–CXCR2 axis guides neutrophil migration during liver transplant IRI, and similar CXCL2 upregulation occurs in cardiomyocytes after ischemia–reperfusion injury [34], suggesting a conserved role for CXCL2 splicing regulation across different IRI contexts. Another conserved event involved GRAMD4, which can mimic p53 function and mediate p73‐dependent mitochondrial apoptosis [35]. Post‐transplantation, an alternative 5 ^′^ splice site shifted toward a proximal site, altering the isoform balance of GRAMD4 and potentially affecting cell death pathways during HIRI.

Notably, mild and severe IRI were associated with distinct splicing programs. The IRI− group predominantly exhibited alternative 5 ^′^/3 ^′^ splice site (alt5p/alt3p) events, whereas the IRI+ group was characterized by intron retention (ir) and overlapping exons (olp) (Figure 2A). This dichotomy suggests that mild IRI was primarily associated with altered splice site selection—which may facilitate rapid inflammatory responses—while severe IRI was characterized by impaired intron removal, suggesting potential spliceosomal dysfunction. Supporting this, genes hosting IRI‐specific splicing events were enriched in pathways related to inflammation, metabolism, and angiogenesis (Figure 2C), whereas IRI+‐specific host genes were enriched in RNA splicing, steroid metabolism, and apoptosis (Figure 2D). Collectively, these findings suggest that HIRI severity is closely associated with distinct splicing programs, pointing to divergent post‐transcriptional regulatory mechanisms. Notably, the histopathological IRI−/IRI+ classification used here represents a proximal and mechanistically appropriate endpoint for this study, distinct from downstream clinical outcomes that are confounded by donor quality, recipient status, and immunosuppression. This grading nevertheless tracks with a clinically relevant injury pathway: disulfide‐HMGB1 increases concomitantly with IRI severity and has been linked to graft dysfunction in liver transplant recipients [36].

Furthermore, the predominance of intron retention events among immune‐associated RAS events (17 of 30; 57%) raises an important functional hypothesis. Intron retention frequently introduces premature termination codons and is a well‐documented trigger of nonsense‐mediated decay (NMD) [37, 38], a pathway that degrades aberrant transcripts and thereby reduces protein output. Given that severe IRI is characterized by intron retention (Figure 1B), it is plausible—though not yet established—that severe injury shifts splicing toward NMD‐prone isoforms, thereby downregulating protein expression of immune and inflammatory genes such as CXCL2, CHI3L1, PNP, and A2M. In contrast, the alternative 5 ^′^/3 ^′^ splice site events (13 of 30; 43%) are expected in principle to generate altered protein isoforms rather than trigger NMD, as exemplified by the GRAMD4 alternative 5 ^′^ splice site event (Figure 2G), which may affect the encoded pro‐apoptotic domain. These predictions are hypotheses for future investigation and will require direct experimental validation.

PNP (Purine Nucleoside Phosphorylase), a key enzyme in the purine nucleotide salvage pathway ubiquitously expressed in cells, also warrants attention. Splicing abnormalities in the PNP gene can lead to severe T‐cell immunodeficiency, including severe combined immunodeficiency (SCID) [39], autoimmune manifestations with immunodeficiency [40], neutropenia [41], or red cell aplasia [42]. This suggests that PNP may be associated with inflammatory regulation in hepatic IRI through its role in purine metabolism and immune cell homeostasis.

Another gene of interest is IL32, a pro‐inflammatory cytokine that has received limited attention in HIRI. In cardiomyocytes, IL‐32 expression is significantly upregulated following hypoxia/reoxygenation, where it exacerbates oxidative stress, inflammation, and apoptosis by activating the NOD2/NOX2/MAPK signaling pathway, thereby worsening myocardial injury [43]. Given the similar hypoxic and inflammatory milieu in HIRI, IL32 may play an analogous role in hepatic IRI, though this remains to be directly tested.

Using CIBERSORT to deconvolute the RNA‐seq data, we estimated the relative proportions of 22 immune cell types across 80 pre‐ and post‐transplant samples. Considerable shifts in immune cell composition were observed after transplantation, indicating a remodeled immune landscape during HIRI.

Mast cells showed a particularly striking change: the proportion of activated mast cells increased while resting mast cells decreased. Although mast cells are scarcely detectable in normal liver tissue, mast cell degranulation in the gastrointestinal tract can mediate liver injury through circulating factors, as demonstrated in a rat model of partial hepatic ischemia. These findings suggest that mast cells may contribute to HIRI via remote degranulation, even without direct hepatic infiltration. NK cells exhibited a similar activation trend after transplantation. Studies indicate that NK cells can recruit macrophages, dendritic cells, and neutrophils to propagate tissue damage [44]. However, certain NK cell subsets, such as ROR*γ*t^+^IL‐22^+^NKp46^+^ cells, appear to exert protective effects and ameliorate IRI [45]. This dual role of NK cells—both pro‐inflammatory and hepatoprotective—suggests they may represent nuanced therapeutic targets in HIRI. Macrophage polarization is a critical determinant of HIRI outcomes. Compared with the IRI− group, the IRI+ group exhibited a more pronounced increase in M0 macrophages and a more substantial decrease in M2 macrophages, indicating a strong association between macrophage polarization shift and IRI severity. Under physiological conditions, resident Kupffer cells in the liver predominantly maintain an M2‐polarized state, secreting anti‐inflammatory factors such as IL‐10 and TGF‐*β* to sustain an immune‐tolerant microenvironment [46]. Our findings suggest that IRI was associated with reduced polarization toward the M2 phenotype, which may delay inflammation resolution. Mechanistically, studies in liver transplantation IRI models have linked Kupffer cell apoptosis to impaired macrophage polarization: reduced apoptosis of M1 macrophages coupled with increased apoptosis of M2 macrophages leads to a pro‐inflammatory imbalance. Thus, IRI was associated with a shift toward a pro‐inflammatory macrophage phenotype, and promoting M2 polarization may represent a promising therapeutic strategy.

The immunomodulatory role of AS is well established. For example, hnRNP M suppresses the splicing of inflammatory genes in macrophages; upon TLR4 activation, this suppression is relieved, enabling expression of factors such as IL‐1*β* [47]. In line with these observations, our co‐expression analysis between RAS events and immune cell proportions identified a significant association between AS and immune remodeling in HIRI. Furthermore, GO enrichment analysis showed that RAS host genes were primarily enriched in pathways central to IRI pathology, including inflammatory immune responses, apoptosis, and oxidation–reduction.

Furthermore, several RAS events correlated significantly with M0 and M2 macrophages, and their host genes were enriched in immune and inflammatory pathways. These findings suggest that AS of these genes was associated with HIRI and were correlated with macrophage polarization‐associated immune cell changes, though the underlying mechanisms warrant further investigation.

Beyond AS events themselves, we observed widespread upregulation of RBPs following HIRI, with most differentially expressed RBPs being upregulated. This pattern raises the possibility that RBP upregulation may represents a compensatory cellular response to stress. Indeed, the RBP ADAR1 has been shown to protect the liver from IRI by suppressing cytosolic RNA‐sensing signaling pathways [48].

RBPs were co‐expressed with AS events associated with HIRI severity, suggesting a potential role in splicing regulation during HIRI. By constructing an RBP‐RAS co‐variation network integrating the 30 immune‐associated RAS events, we identified 43 RBPs that co‐vary with these events. This network‐based observation is supported by a previous study on steatotic liver grafts, which found that aberrant splicing events were enriched in metabolic and immune‐related pathways, and RBPs were correlated with splicing changes in the hepatic immune environment by binding to aberrant splicing regulatory elements [14]. Collectively, these findings are consistent with the hypothesis that RBPs may contribute to hepatic immune and metabolic changes through their association with RAS.

RBPs also participate in the regulation of immune cell function. ZFP36, one of the key RBPs identified in our study, suppresses pro‐inflammatory cytokine production (e.g., TNF‐*α*) in macrophages [49]. Mechanistically, under stress conditions, ZFP36‐bound mRNAs are recruited to stress granules, where the translational repressor TIA‐1 prevents their translation; ZFP36 also facilitates mRNA degradation by transporting transcripts from stress granules to the cytoplasm [50–52]. These properties suggest ZFP36 may participate in the regulation of inflammatory response in HIRI.

Our findings of ZFP36 dysregulation in HIRI are independently supported by a recent experimental study. Du et al. demonstrated, using quantitative real‐time PCR (qRT‐PCR) and IHC, that ZFP36L1, a homolog of ZFP36, was significantly downregulated in human DCD liver graft biopsies after reperfusion [32]. This independent experimental evidence supports the biological relevance of the RBP–AS–immune associations identified in our analysis.

Similarly, ZC3H12A (Regnase‐1), an RNase essential for immune control, promotes degradation of multiple pro‐inflammatory cytokine mRNAs, including IL‐1, IL‐2, IL‐6, CXCL1, CXCL2, and CXCL3 [53]. The I*κ*B kinase complex regulates the stability of these cytokine‐encoding mRNAs by controlling Regnase‐1 degradation [54]. Thus, ZC3H12A may also limit excessive inflammatory responses in HIRI. Collectively, these findings suggest that RBPs may contribute to HIRI pathogenesis through their association with AS and immune‐related transcriptional changes.

Single‐cell transcriptomic analysis provided deeper insight into cell‐type‐specific changes during HIRI. Using GSE171539 (pre‐transplant vs. 2‐h post‐reperfusion), we identified 19 cell populations, with the most significantly altered clusters including hepatocytes, endothelial cells, smooth muscle cells, and macrophages. GO enrichment analysis of upregulated genes in each cluster aligned with annotated cell types (e.g., plasma cells enriched in immune and stress response pathways).

Importantly, single‐cell resolution revealed distinct expression patterns of the RBPs identified in our bulk analysis. ZFP36 showed notable differential expression in hepatocytes and proliferating cells, suggesting that its regulatory activity may be primarily exerted within these populations. In contrast, MBNL2 exhibited broader expression across multiple cell types, including endothelial cells, ILCs, hepatocytes, and macrophages, implying a more general regulatory role. These cell‐type‐specific patterns support the hypothesis that RBPs contribute to HIRI through context‐dependent splicing regulation.

The widespread RBP upregulation identified in our primary analysis was independently validated in the GSE12720 cohort [55], where 39 of 42 detected RBPs were significantly upregulated post‐reperfusion. This directional consistency across two independent datasets and platforms (RNA‐seq and microarray) strengthens the robustness of the observed RBP dysregulation in HIRI. Protein‐level evidence from the Human Protein Atlas [56] further confirmed that 25 of 43 RBPs (58%) are expressed in normal liver tissue, with KRT18, HSPA1A, and HSP90AB1 showing medium‐to‐high hepatocyte expression. ZFP36, ZC3H12A, and ZFP36L1 were preferentially detected in cholangiocytes, consistent with immune‐regulatory rather than hepatocyte‐intrinsic roles. Although 18 RBPs lack HPA IHC evidence in normal liver, all are annotated as “Evidence at protein level” (PE1) in UniProt, consistent with their stress‐induced expression; quantitative proteomics of post‐reperfusion liver biopsies would provide direct protein‐level validation under ischemia–reperfusion conditions. Collectively, these independent validations support the biological relevance of the identified RBP candidates and provide a more robust foundation for future hypothesis‐driven investigation.

Taken together, this study is explicitly hypothesis‐generating. The identified splicing events, RBP candidates, and immune associations represent a resource for formulating testable hypotheses about post‐transcriptional regulation in HIRI. Independent experimental validation—including modulation of candidate RBPs in cellular or animal models of hepatic IRI—will be essential to establish whether the associations reported here reflect causal regulatory mechanisms.

Several limitations should be acknowledged. First, as an observational bioinformatics study, causal relationships cannot be established; functional validation in vivo and in vitro is required. Second, sample sizes are modest (*n* = 40 for bulk RNA‐seq; *n* = 1 donor with three longitudinal time pointsfor single‐cell). In addition, individual‐level clinical outcome data—including EAD, graft survival, and rejection episodes—were not available in the public repository and therefore could not be correlated with the identified splicing events or RBPs; such clinical–molecular correlation represents an important direction for future studies with access to linked clinical and molecular data. In addition, the bulk RNA‐seq cohort derives from a single transplant center; while the RBP expression findings were independently validated in a separate‐center cohort (GSE12720), the AS findings remain derived from a single cohort, and their generalizability to other populations and transplant centers requires validation in independent RNA‐seq cohorts. The single‐donor design of GSE171539 precludes formal statistical inference and limits generalizability; accordingly, these analyses should be viewed as qualitative, hypothesis‐generating support for cell‐type‐specific RBP expression patterns. Third, CIBERSORT deconvolution infers immune cell proportions from bulk RNA‐seq data using the LM22 reference matrix and a support vector regression approach. These are computational estimates rather than direct measurements (e.g., flow cytometry or IHC). Their accuracy depends on how well the LM22 signature—derived primarily from peripheral blood mononuclear cells—captures the transcriptional states of tissue‐resident and infiltrating immune cells in the hepatic microenvironment. Absolute cell fraction values should therefore be interpreted with caution; relative changes between groups and time points are more informative than absolute quantification. Furthermore, CIBERSORT cannot resolve spatially organized immune structures (e.g., portal infiltrates versus lobular inflammation), which may be relevant in HIRI. Despite these limitations, CIBERSORT is a validated and widely used method for immune deconvolution of bulk transcriptomic data, and our key findings—mast cell activation and M0/M2 macrophage imbalance—are consistent with prior experimental studies of hepatic IRI that employed immunohistochemical quantification. Fourth, our RBP‐RAS network correlation threshold (|0.6|) may include false positives. Although a permutation test (1000 label shuffles) yielded zero RBP‐RAS edges under the null (empirical *p* < 0.001) with a bootstrap stability of 81.8% (Table S18), and although the |r| thresholds are more stringent than Bonferroni‐corrected significance, the analytical thresholds (frequency ≥ 0.2, pSAR ≥ 50*%*, |r| ≥ 0.6/0.5) were not formally pre‐registered. We have mitigated this by fixing the thresholds a priori from the SUVA framework and Cohen’s conventions and by providing full data transparency (Tables S1, S2, S3, S4, S5, S6, S7, S8, S9, S10, S11, S12, S13, S14, S15, S16, S17, and S18) so that the results can be reproduced under alternative thresholds. Fifth, the single‐cell dataset (GSE171539) derives from a single donor and serves only as exploratory validation; it validates RBP expression but not the splicing events themselves, as splice junction coverage in droplet‐based scRNA‐seq is insufficient for reliable isoform‐level quantification. None of the conclusions regarding the RAS events depend on the single‐cell data; these findings rest solely on the bulk RNA‐seq SUVA analysis. Sixth, we did not assess whether the identified RAS events lead to NMD or altered protein isoforms; determining these functional consequences requires splice junction genomic coordinates and transcript annotation not retained in the present analysis, and direct validation using RNA stability assays (e.g., cycloheximide treatment) or ribosome profiling will be required. Despite these limitations, the findings presented herein generate testable hypotheses regarding post‐transcriptional regulation in HIRI and provide a resource for future experimental investigation. Future studies employing RBP knockdown or overexpression in hepatocyte or Kupffer cell lines, splicing minigene reporter assays for candidate RAS events (e.g., CXCL2 intron retention, GRAMD4 alternative 5 ^′^ splice site), and RNA immunoprecipitation (RIP) or crosslinking immunoprecipitation (CLIP‐seq) of key RBPs such as ZFP36 and ZC3H12A will be needed to determine whether the correlations reported here reflect direct causal regulatory relationships.

## 5. Conclusion

In summary, this study provides evidence suggesting that AS events and RBPs are dynamically regulated during HIRI in liver transplantation. Notably, mild and severe IRI are associated with distinct splicing programs—alternative splice site usage versus intron retention/overlapping exons—suggesting progressively impaired spliceosomal function with increasing injury severity. These AS changes are closely associated with immune microenvironment remodeling, including mast cell activation and M0/M2 macrophage imbalance. Single‐cell analysis further identifies cell‐type‐specific expression patterns of key RBPs, such as ZFP36 and MBNL2, pointing to context‐dependent regulatory roles. Collectively, our findings support the hypothesis of an RBP–AS–immune regulatory network in HIRI, provide a basis for generating hypotheses regarding post‐transcriptional regulation, and nominate highly conserved splicing events and RBPs as candidates for future investigation as potential biomarkers and therapeutic targets.

**Abbreviations**
AS: alternative splicing
DAS: differentially alternative spliced
DEG: differentially expressed gene
EAD: early allograft dysfunction
FC: fold change
FDR: false discovery rate
FPKM: fragments per kilobase of exon per million mapped fragments
GO: Gene Ontology
HIF‐1*α*
: hypoxia‐inducible factor 1‐alpha
HIRI: hepatic ischemia–reperfusion injury
IRI+: severe IRI
IRI−: mild IRI
KEGG: Kyoto Encyclopedia of Genes and Genomes
NF‐*κ*B: nuclear factor kappa‐B
PCA: principal component analysis
pSAR: reads proportion of SUVA AS event
RAS: regulated alternative splicing
RBP: RNA‐binding protein
SIRS: systemic inflammatory response syndrome
TPM: tags per million
UMAP: uniform manifold approximation and projection

## Author Contributions

Hanfei Huang conceptualized the study and provided financial assistance. The study design was developed by Bincheng Gui. Bincheng Gui was responsible for collecting and analyzing the data. Bincheng Gui wrote and interpreted the manuscript. Hanfei Huang reviewed and revised the manuscript.

## Funding

The Natural Science Foundation of Yunnan Province, No. 202302AA310025; The Key Clinical Specialty Fund for Organ Transplantation in Yunnan Province, No. 300068; The Yunnan Key Laboratory of Organ Transplantation, No. 202449CE340016; Noncommunicable Chronic Diseases‐National Science and Technology Major Project of China, No.2025ZD0552100.

## Disclosure

The final version of the manuscript was read and approved by all authors.

## Ethics Statement

This study was conducted in accordance with the Declaration of Helsinki. All data were obtained from publicly available repositories (GSE151648, GSE171539, GSE12720, HPA).

## Consent

The authors have nothing to report.

## Conflicts of Interest

The authors declare no conflicts of interest.

## Supporting Information

Additional supporting information can be found online in the Supporting Information section.

## Supporting information


**Supporting Information 1** Figure S1: Identification of highly conserved regulated AS events (RAS) between samples before and after liver transplantation. Figure S2: Comparison of RAS between samples before and after liver transplantation with different IRI severity. Figure S3: Diversity of immune microenvironment characteristics between samples before and after liver transplantation underlying IRI− or IRI+. Figure S4: Dynamic changes of alternative splicing events associated with immune microenvironment regulation in samples before and after liver transplantation patients with varying IRI degrees. Figure S5: Construction of co‐disturbed network between RNA‐binding proteins (RBP) and immune‐infiltration‐associated RAS. Figure S6: Single‐cell transcriptomic analysis delineates regulated expression of RBPs in heterogeneous immune cell populations in samples before and after liver transplantation. Figure S7: Human Protein Atlas validation of 43 RBPs in normal liver tissue. Figure S8: External validation of RBP expression in GSE12720 independent cohort. Figure S9: In silico perturbation analysis of the RBP‐AS‐immune regulatory network.

**Supporting Information 2** Text S1: Complete analytical pipeline pseudocode.

**Supporting Information 3** Table S1: All identified RAS events (*n* = 10,290) with SUVA statistics.

**Supporting Information 4** Table S2: pSAR matrix of high‐confidence RAS events across 80 samples.

**Supporting Information 5** Table S3: FPKM expression matrix of 43 differentially expressed RBPs.

**Supporting Information 6** Table S4: CIBERSORT‐estimated immune cell fractions for 22 cell types.

**Supporting Information 7** Table S5: RBP‐RAS co‐expression network edge table.

**Supporting Information 8** Table S6: Correlation matrix of 30 immune‐associated RAS events with 22 immune cell types.

**Supporting Information 9** Table S7: Comprehensive table of 30 immune‐associated RAS events with host genes, splicing types, pSAR values, and immune cell correlations.

**Supporting Information 10** Table S8: Comprehensive table of 43 RBPs with expression fold‐changes and correlated RAS events.

**Supporting Information 11** Table S9: Clinical and demographic characteristics of GSE151648 and GSE171539 cohorts.

**Supporting Information 12** Table S10: Sensitivity analysis for all analytical thresholds.

**Supporting Information 13** Table S11: Human Protein Atlas protein expression levels for 43 RBPs in liver tissue.

**Supporting Information 14** Table S12: GSE12720 external validation differential expression results for 43 RBPs.

**Supporting Information 15** Table S13: Summary of the SRA‐derived per‐sample sequencing statistics (read counts, read length) and post‐alignment quality control metrics (genes detected per sample), demonstrating consistent sequencing depth and uniform gene detection across all 80 samples and four groups.

**Supporting Information 16** Table S14: Network criticality of 43 RBPs.

**Supporting Information 17** Table S15: Virtual perturbation.

**Supporting Information 18** Table S16: Partial correlation.

**Supporting Information 19** Table S17: Combined multilayer validation.

**Supporting Information 20** Table S18: Permutation bootstrap validation.

**Supporting Information 21** Table S19: UniProt protein evidence of 18 RBPs.

**Supporting Information 22**  

## Data Availability

The datasets supporting the conclusions of this article are available in public repositories. The bulk RNA‐seq data (GSE151648) and single‐cell RNA‐seq data (GSE171539) were obtained from the NCBI Gene Expression Omnibus (GEO) repository (https://www.ncbi.nlm.nih.gov/geo/). The external validation microarray dataset (GSE12720) is also available at GEO. Protein expression data were obtained from the Human Protein Atlas (v23, https://www.proteinatlas.org). All analyzed data generated during this study are included in this published article and its supporting information files.
