# Supplementary material for: Comprehensive and Combined Omics Analysis Reveals Immune–Infiltration‐Associated Alternative Splicing and RBP Regulators Contributing to Ischemia–Reperfusion Injury in Liver Transplantation
Source: Int J Genomics. 2026 Sep 21;2026:4835678. doi: 10.1155/ijog/4835678 (PMC13592132; doi:10.1155/ijog/4835678)
Supplement: Supplementary file 1 [file IJOG-2026-4835678-s016.docx]

**Supplementary Figures**

**Figure S1**. Identification of highly conserved regulated AS events (RAS) between samples before and after liver transplantation.

A. Boxplot showing all detected AS events by SUVA across paired liver biopsies samples of pre-transplant and post-transplant from 17 IRI- patients and 23 IRI+ patients, respectively.

B. Boxplot showing number of AS events contains SJs involved in two or more different classical splicing events (complex) of in same classical splicing event (simple).

C. Splice junction constituting AS events detected by SUVA was annotated to classical AS event types. And the number of each classical AS event types were showed with boxplot.

D．Principal component analysis (PCA) of splicing ratio of RAS which frequency>=0.2 and pSAR (Reads proportion of SUVA AS event) >=50%. The samples were grouped by pre and post transplantation from IRI-/IRI+ patients and the ellipse for each group is the confidence ellipse.

E．Heatmap of splicing ratio across all samples for RAS which frequency>=0.5 and pSASR >=50% and corresponding genes.

**Figure S2**. Comparison of RAS between samples before and after liver transplantation with different IRI severity. P-values shown are Benjamini-Hochberg corrected (FDR).

A.	KEGG enrichment for host genes of overlapped RAS of IRI- and IRI+.

B.	KEGG enrichment for host genes of IRI- specific RAS.

C.	KEGG enrichment for host genes of IRI+ specific RAS.

**Figure S3**. Diversity of immune microenvironment characteristics between samples before and after liver transplantation underlying IRI- or IRI+.
A. Fractions of different cells estimated by CIBERSORT in each sample.
B. Principal component analysis (PCA) based on fractions of different cells in samples before (PRE) and after (POST) liver transplantation from IRI- or IRI+ patients. The ellipse for each group is the confidence ellipse.
C-D. Boxplot showing cell fraction of 6 cell types.

**Figure S4**. Dynamic changes of alternative splicing events associated with immune microenvironment regulation in samples before and after liver transplantation patients with varying IRI degrees.
A. Boxplot showing splicing ratio profile across 80 samples of 30 co-disturbed RAS with immune cells from figure 4B.

**Figure S5**. Construction of co-disturbed network between RNA-binding proteins (RBP) and immune-infiltration-associated RAS.
A. Boxplot showing expression profile of RBPs regulators from Figure 5B.

**Figure S6**. Single-cell transcriptomic analysis delineates regulated expression of RBPs in heterogeneous immune cell populations in samples before and after liver transplantation.

A. Dotmap showing expression levels of top 3 markers in each cell type.

B. Percentages of assigned cell types within each cluster in each group were calculated.

C. Scatter plot comparing the proportions of cell populations of each cell type in two groups.

D-E. Expression of RBP KRT18 and ZC3H12A were used to label clusters by cell identity as represented in the UMAP plot for Post and Pre groups separately.

F-G. Violin plot showing expression alternation of KRT18 and ZC3H12A between Post and Pre sample in different cell clusters.

**Figure S7** Human Protein Atlas validation of 43 RBPs in normal liver tissue.

A. Protein expression heatmap of 43 RBPs across hepatocytes and cholangiocytes, ordered by total expression score. Color intensity indicates protein level: High (red), Medium (orange), Low (blue), Not detected (grey), No data (white). Right sidebar indicates HPA reliability score (Enhanced, Supported, Approved, Uncertain, Not in liver, Not in HPA).
B. Key RBP expression detail for 15 representative RBPs. KRT18, HSPA1A, and HSP90AB1 show medium-to-high hepatocyte expression (Enhanced/Supported reliability); ZFP36, ZC3H12A, and ZFP36L1 show preferential expression in cholangiocytes. Data source: Human Protein Atlas v23 (Uhlén et al.,Science 2015).

**Figure S8** External validation of RBP expression in GSE12720 independent cohort

A． Volcano plot showing differential expression results for 20 paired pre-ischemia vs. post-reperfusion liver transplant biopsies. The 42 detected RBPs are highlighted in red; top 20 significant RBPs are labeled. Dashed line indicates FDR = 0.05.
B． log2 fold change of 30 key RBPs. Bar color intensity indicates magnitude of change (red = up, blue = down). Significance levels: *P < 0.05, **P < 0.01, ***P < 0.001 (Benjamini-Hochberg FDR).
C． Summary of directional consistency: 41 of 42 RBPs (98%) were upregulated post-reperfusion, consistent with the primary GSE151648 analysis.

**Figure S9**. In silico perturbation analysis of the RBP-AS-immune regulatory network.

A Network criticality ranking of the top 18 RBPs by in silico knockout. Bar color indicates manuscript-highlighted RBPs (red) versus other network RBPs (grey). Degree (D) values indicate the number of directly connected RAS events for each RBP.

B Virtual expression perturbation effects (Cohen's d) comparing immune cell proportions between RBP-high (top quartile) and RBP-low (bottom quartile) groups. The top three effect sizes per immune cell type are shown; all displayed pairs pass FDR < 0.05. RBP names are shown on the left; immune cell types on the right, color-coded as mast cells activated (purple), NK cells activated (teal), M0 macrophages (orange), and M2 macrophages (blue).

C Partial Spearman correlation heatmap of RBP–immune cell associations that remain significant (FDR < 0.05) after controlling for the expression of all other 42 network RBPs. Manuscript-highlighted RBPs are labeled in bold red.

D Multi-layer evidence summary for 20 key RBPs across three independent analytical dimensions: network criticality (N, score > 1.0), virtual expression perturbation (P, ≥ 2 significant immune cell types, FDR < 0.05), and partial correlation specificity (C, ≥ 1 significant immune cell type, FDR < 0.05). Filled circles (●) indicate the criterion was met; open circles (○) indicate the criterion was not met.
