# Supplementary material for: Comprehensive and Combined Omics Analysis Reveals Immune–Infiltration‐Associated Alternative Splicing and RBP Regulators Contributing to Ischemia–Reperfusion Injury in Liver Transplantation
Source: Int J Genomics. 2026 Sep 21;2026:4835678. doi: 10.1155/ijog/4835678 (PMC13592132; doi:10.1155/ijog/4835678)
Supplement: Supplementary file 2 [file IJOG-2026-4835678-s019.zip › Supplementary_Enrichment_Results/genelist_GO_analysis/genelist_GO_enrichment_C_top.pdf]

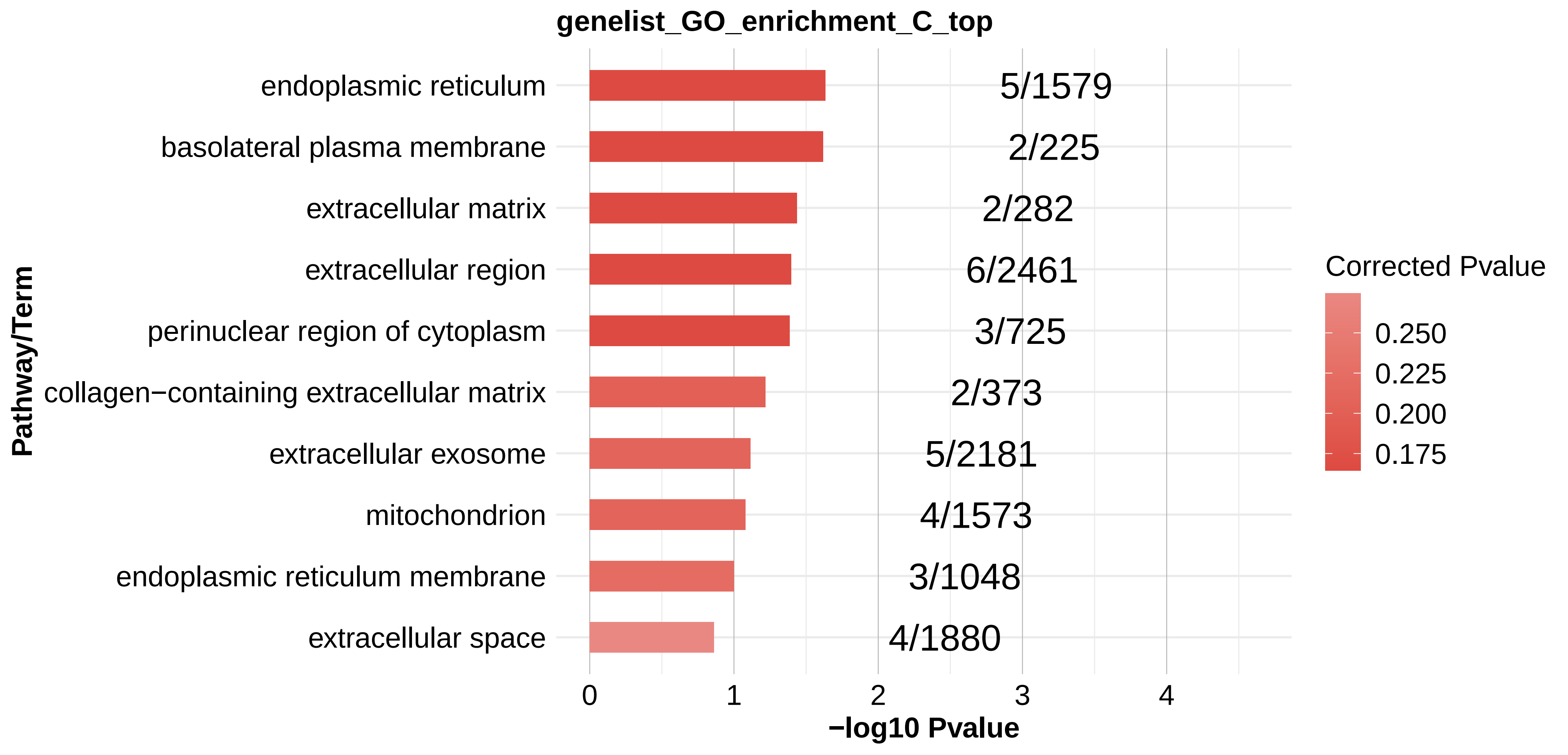
genelist_GO_enrichment_C_top
Pathway/Term
endoplasmic reticulum
basolateral plasma membrane
extracellular matrix
extracellular region
perinuclear region of cytoplasm
collagen−containing extracellular matrix
extracellular exosome
mitochondrion
endoplasmic reticulum membrane
extracellular space
5/1579
2/225
2/282
6/2461
3/725
2/373
5/2181
4/1573
3/1048
4/1880
0
1
2
3
4
−log10 Pvalue
Corrected Pvalue
0.250
0.225
0.200
0.175
