# Supplementary material for: Comprehensive and Combined Omics Analysis Reveals Immune–Infiltration‐Associated Alternative Splicing and RBP Regulators Contributing to Ischemia–Reperfusion Injury in Liver Transplantation
Source: Int J Genomics. 2026 Sep 21;2026:4835678. doi: 10.1155/ijog/4835678 (PMC13592132; doi:10.1155/ijog/4835678)
Supplement: Supplementary file 2 [file IJOG-2026-4835678-s019.zip › Supplementary_Enrichment_Results/genelist_GO_analysis/genelist_GO_enrichment_P_top.pdf]

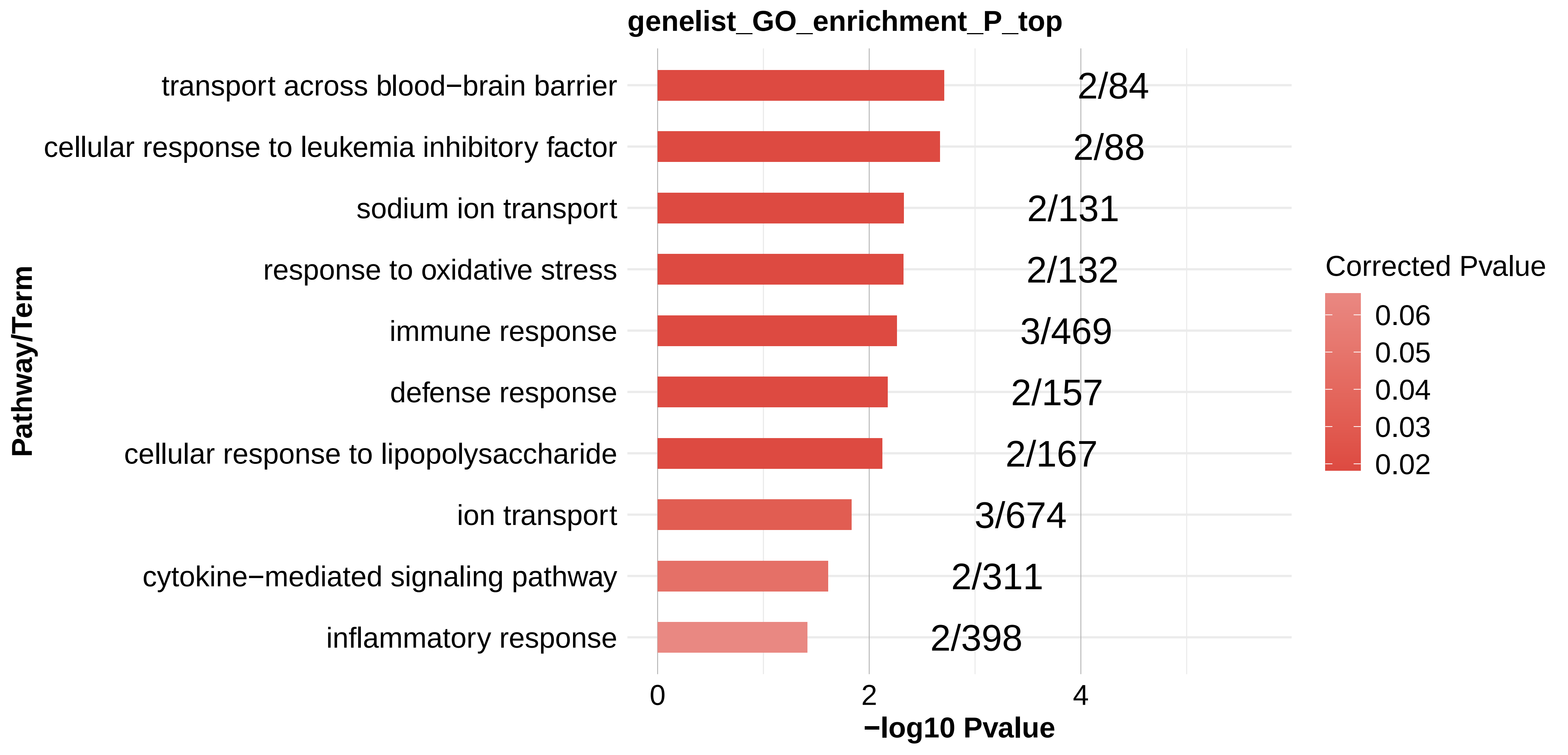
genelist_GO_enrichment_P_top
Pathway/Term
transport across blood−brain barrier
cellular response to leukemia inhibitory factor
sodium ion transport
response to oxidative stress
immune response
defense response
cellular response to lipopolysaccharide
ion transport
cytokine−mediated signaling pathway
inflammatory response
2/84
2/88
2/131
2/132
3/469
2/157
2/167
3/674
2/311
2/398
0
2
4
−log10 Pvalue
Corrected Pvalue
0.06
0.05
0.04
0.03
0.02
