# Supplementary material for: Comprehensive and Combined Omics Analysis Reveals Immune–Infiltration‐Associated Alternative Splicing and RBP Regulators Contributing to Ischemia–Reperfusion Injury in Liver Transplantation
Source: Int J Genomics. 2026 Sep 21;2026:4835678. doi: 10.1155/ijog/4835678 (PMC13592132; doi:10.1155/ijog/4835678)
Supplement: Supplementary file 2 [file IJOG-2026-4835678-s019.zip › Supplementary_Enrichment_Results/genelist_GO_analysis/genelist_GO_enrichment_web_C.html]

GO enrichment analyze


# GO enrichment analyze result


| Term | Database | ID | Input number (20) | Background number (18576) | P-Value | Corrected P-Value | Input | Hyperlink |
| --- | --- | --- | --- | --- | --- | --- | --- | --- |
| endoplasmic reticulum | Gene Ontology | GO:0005783 | 5 (25.00%) | 1579 (8.50%) | 0.0232153909253 | 0.164639610994 | FADS1  TGM2  VMP1  CHI3L1  GRAMD4 | web link |
| basolateral plasma membrane | Gene Ontology | GO:0016323 | 2 (10.00%) | 225 (1.21%) | 0.0240439482406 | 0.164639610994 | SLC22A7  SLC5A6 | web link |
| extracellular matrix | Gene Ontology | GO:0031012 | 2 (10.00%) | 282 (1.52%) | 0.036450735424 | 0.164639610994 | TGM2  CHI3L1 | web link |
| extracellular region | Gene Ontology | GO:0005576 | 6 (30.00%) | 2461 (13.25%) | 0.0400235006009 | 0.164639610994 | TGM2  CXCL2  PNP  IL32  CHI3L1  A2M | web link |
| perinuclear region of cytoplasm | Gene Ontology | GO:0048471 | 3 (15.00%) | 725 (3.90%) | 0.0411599027486 | 0.164639610994 | TGM2  CHI3L1  TMEM134 | web link |
| collagen-containing extracellular matrix | Gene Ontology | GO:0062023 | 2 (10.00%) | 373 (2.01%) | 0.0602321318263 | 0.200773772754 | TGM2  A2M | web link |
| extracellular exosome | Gene Ontology | GO:0070062 | 5 (25.00%) | 2181 (11.74%) | 0.0766208567711 | 0.208271043321 | PNP  TGM2  A2M  CHI3L1  CDC37 | web link |
| mitochondrion | Gene Ontology | GO:0005739 | 4 (20.00%) | 1573 (8.47%) | 0.0833084173283 | 0.208271043321 | FADS1  TGM2  PLIN5  GRAMD4 | web link |
| endoplasmic reticulum membrane | Gene Ontology | GO:0005789 | 3 (15.00%) | 1048 (5.64%) | 0.0998944672182 | 0.221987704929 | FADS1  VMP1  GRAMD4 | web link |
| extracellular space | Gene Ontology | GO:0005615 | 4 (20.00%) | 1880 (10.12%) | 0.13720974774 | 0.274419495481 | CXCL2  IL32  A2M  CHI3L1 | web link |
| cytosol | Gene Ontology | GO:0005829 | 8 (40.00%) | 5296 (28.51%) | 0.18435067094 | 0.312070328965 | TGM2  NOP58  TMEM134  PNP  CDC37  IL32  PLIN5  A2M | web link |
| integral component of plasma membrane | Gene Ontology | GO:0005887 | 3 (15.00%) | 1398 (7.53%) | 0.187242197379 | 0.312070328965 | SLC22A7  SLC5A6  SLC38A2 | web link |
| nucleolus | Gene Ontology | GO:0005730 | 2 (10.00%) | 848 (4.57%) | 0.231426412004 | 0.356040633852 | VMP1  NOP58 | web link |
| intracellular membrane-bounded organelle | Gene Ontology | GO:0043231 | 2 (10.00%) | 998 (5.37%) | 0.292308062446 | 0.400272347984 | FADS1  PLIN5 | web link |
| cytoplasm | Gene Ontology | GO:0005737 | 9 (45.00%) | 6872 (36.99%) | 0.300204260988 | 0.400272347984 | TGM2  SRSF3  TMEM134  SLC38A2  RCAN1  PNP  CDC37  CHI3L1  PLIN5 | web link |
| integral component of membrane | Gene Ontology | GO:0016021 | 7 (35.00%) | 5472 (29.46%) | 0.37124082044 | 0.46405102555 | VMP1  TMEM134  SLC38A2  SLC22A7  SLC5A6  GRAMD4  FADS1 | web link |
| nucleus | Gene Ontology | GO:0005634 | 8 (40.00%) | 6548 (35.25%) | 0.408172246578 | 0.479304288541 | SBNO2  TGM2  NOP58  SRSF3  RCAN1  PNP  FADS1  NCOA7 | web link |
| membrane | Gene Ontology | GO:0016020 | 10 (50.00%) | 8478 (45.64%) | 0.431373859687 | 0.479304288541 | VMP1  NOP58  TMEM134  SLC38A2  TGM2  SLC22A7  IL32  SLC5A6  GRAMD4  FADS1 | web link |
| plasma membrane | Gene Ontology | GO:0005886 | 5 (25.00%) | 5167 (27.82%) | 0.69182664153 | 0.728238570032 | VMP1  SLC22A7  TGM2  SLC5A6  SLC38A2 | web link |
| nucleoplasm | Gene Ontology | GO:0005654 | 2 (10.00%) | 3824 (20.59%) | 0.938540758497 | 0.938540758497 | NOP58  SRSF3 | web link |
