# Supplementary material for: Comprehensive and Combined Omics Analysis Reveals Immune–Infiltration‐Associated Alternative Splicing and RBP Regulators Contributing to Ischemia–Reperfusion Injury in Liver Transplantation
Source: Int J Genomics. 2026 Sep 21;2026:4835678. doi: 10.1155/ijog/4835678 (PMC13592132; doi:10.1155/ijog/4835678)
Supplement: Supplementary file 2 [file IJOG-2026-4835678-s019.zip › Supplementary_Enrichment_Results/genelist_GO_analysis/genelist_GO_enrichment_web_F.html]

GO enrichment analyze


# GO enrichment analyze result


| Term | Database | ID | Input number (19) | Background number (18303) | P-Value | Corrected P-Value | Input | Hyperlink |
| --- | --- | --- | --- | --- | --- | --- | --- | --- |
| protein binding | Gene Ontology | GO:0005515 | 19 (100.00%) | 13627 (74.45%) | 0.00366680782262 | 0.0266720124867 | TGM2  PLIN5  NCOA7  NOP58  TMEM134  SLC38A2  RCAN1  PNP  CHI3L1  VMP1  CXCL2  CDC37  SLC22A7  IL32  SLC5A6  GRAMD4  CCDC9  A2M  SRSF3 | web link |
| symporter activity | Gene Ontology | GO:0015293 | 2 (10.53%) | 119 (0.65%) | 0.00666800312168 | 0.0266720124867 | SLC5A6  SLC38A2 | web link |
| transmembrane transporter activity | Gene Ontology | GO:0022857 | 2 (10.53%) | 206 (1.13%) | 0.0190075201137 | 0.0482432884066 | SLC22A7  SLC5A6 | web link |
| cytokine activity | Gene Ontology | GO:0005125 | 2 (10.53%) | 234 (1.28%) | 0.0241216442033 | 0.0482432884066 | CXCL2  IL32 | web link |
| identical protein binding | Gene Ontology | GO:0042802 | 4 (21.05%) | 1628 (8.89%) | 0.082303790829 | 0.131686065326 | RCAN1  PNP  TGM2  PLIN5 | web link |
| nucleic acid binding | Gene Ontology | GO:0003676 | 2 (10.53%) | 585 (3.20%) | 0.12205515456 | 0.16274020608 | RCAN1  SRSF3 | web link |
| RNA binding | Gene Ontology | GO:0003723 | 3 (15.79%) | 1527 (8.34%) | 0.207782923285 | 0.23746619804 | CCDC9  NOP58  SRSF3 | web link |
| transferase activity | Gene Ontology | GO:0016740 | 2 (10.53%) | 1849 (10.10%) | 0.585662249187 | 0.585662249187 | PNP  TGM2 | web link |
