# Supplementary material for: Comprehensive and Combined Omics Analysis Reveals Immune–Infiltration‐Associated Alternative Splicing and RBP Regulators Contributing to Ischemia–Reperfusion Injury in Liver Transplantation
Source: Int J Genomics. 2026 Sep 21;2026:4835678. doi: 10.1155/ijog/4835678 (PMC13592132; doi:10.1155/ijog/4835678)
Supplement: Supplementary file 2 [file IJOG-2026-4835678-s019.zip › Supplementary_Enrichment_Results/genelist_GO_analysis/genelist_GO_enrichment_web_P.html]

GO enrichment analyze


# GO enrichment analyze result


| Term | Database | ID | Input number (14) | Background number (17636) | P-Value | Corrected P-Value | Input | Hyperlink |
| --- | --- | --- | --- | --- | --- | --- | --- | --- |
| transport across blood-brain barrier | Gene Ontology | GO:0150104 | 2 (14.29%) | 84 (0.48%) | 0.00196549955345 | 0.0182795035291 | SLC5A6  SLC38A2 | web link |
| cellular response to leukemia inhibitory factor | Gene Ontology | GO:1990830 | 2 (14.29%) | 88 (0.50%) | 0.00215441906616 | 0.0182795035291 | SBNO2  SRSF3 | web link |
| sodium ion transport | Gene Ontology | GO:0006814 | 2 (14.29%) | 131 (0.74%) | 0.00469983257772 | 0.0182795035291 | SLC5A6  SLC38A2 | web link |
| response to oxidative stress | Gene Ontology | GO:0006979 | 2 (14.29%) | 132 (0.75%) | 0.00476997692374 | 0.0182795035291 | RCAN1  NCOA7 | web link |
| immune response | Gene Ontology | GO:0006955 | 3 (21.43%) | 469 (2.66%) | 0.00546650027274 | 0.0182795035291 | PNP  CXCL2  IL32 | web link |
| defense response | Gene Ontology | GO:0006952 | 2 (14.29%) | 157 (0.89%) | 0.00668004089627 | 0.0182795035291 | CXCL2  IL32 | web link |
| cellular response to lipopolysaccharide | Gene Ontology | GO:0071222 | 2 (14.29%) | 167 (0.95%) | 0.00752685439434 | 0.0182795035291 | SBNO2  CXCL2 | web link |
| ion transport | Gene Ontology | GO:0006811 | 3 (21.43%) | 674 (3.82%) | 0.0147591804562 | 0.0313632584693 | SLC22A7  SLC5A6  SLC38A2 | web link |
| cytokine-mediated signaling pathway | Gene Ontology | GO:0019221 | 2 (14.29%) | 311 (1.76%) | 0.0245266882092 | 0.0463281888396 | CXCL2  IL32 | web link |
| inflammatory response | Gene Ontology | GO:0006954 | 2 (14.29%) | 398 (2.26%) | 0.038649603912 | 0.0657043266505 | CXCL2  CHI3L1 | web link |
| neutrophil degranulation | Gene Ontology | GO:0043312 | 2 (14.29%) | 480 (2.72%) | 0.0542012192192 | 0.0837655206115 | PNP  CHI3L1 | web link |
| positive regulation of gene expression | Gene Ontology | GO:0010628 | 2 (14.29%) | 509 (2.89%) | 0.0601655478672 | 0.0852345261452 | IL32  SLC38A2 | web link |
| transmembrane transport | Gene Ontology | GO:0055085 | 2 (14.29%) | 641 (3.63%) | 0.0899621261876 | 0.114241483352 | SLC22A7  SLC5A6 | web link |
| cell adhesion | Gene Ontology | GO:0007155 | 2 (14.29%) | 658 (3.73%) | 0.0940812215843 | 0.114241483352 | VMP1  IL32 | web link |
| apoptotic process | Gene Ontology | GO:0006915 | 2 (14.29%) | 692 (3.92%) | 0.10248958821 | 0.116154866638 | CHI3L1  GRAMD4 | web link |
| positive regulation of transcription by RNA polymerase II | Gene Ontology | GO:0045944 | 2 (14.29%) | 1149 (6.52%) | 0.230673400013 | 0.245090487514 | SBNO2  NCOA7 | web link |
| regulation of transcription, DNA-templated | Gene Ontology | GO:0006355 | 2 (14.29%) | 1422 (8.06%) | 0.313345767958 | 0.313345767958 | FADS1  SBNO2 | web link |
