# Supplementary material for: Comprehensive and Combined Omics Analysis Reveals Immune–Infiltration‐Associated Alternative Splicing and RBP Regulators Contributing to Ischemia–Reperfusion Injury in Liver Transplantation
Source: Int J Genomics. 2026 Sep 21;2026:4835678. doi: 10.1155/ijog/4835678 (PMC13592132; doi:10.1155/ijog/4835678)
Supplement: Supplementary file 2 [file IJOG-2026-4835678-s019.zip › Supplementary_Enrichment_Results/genelist_GO_analysis/Rplots.pdf]

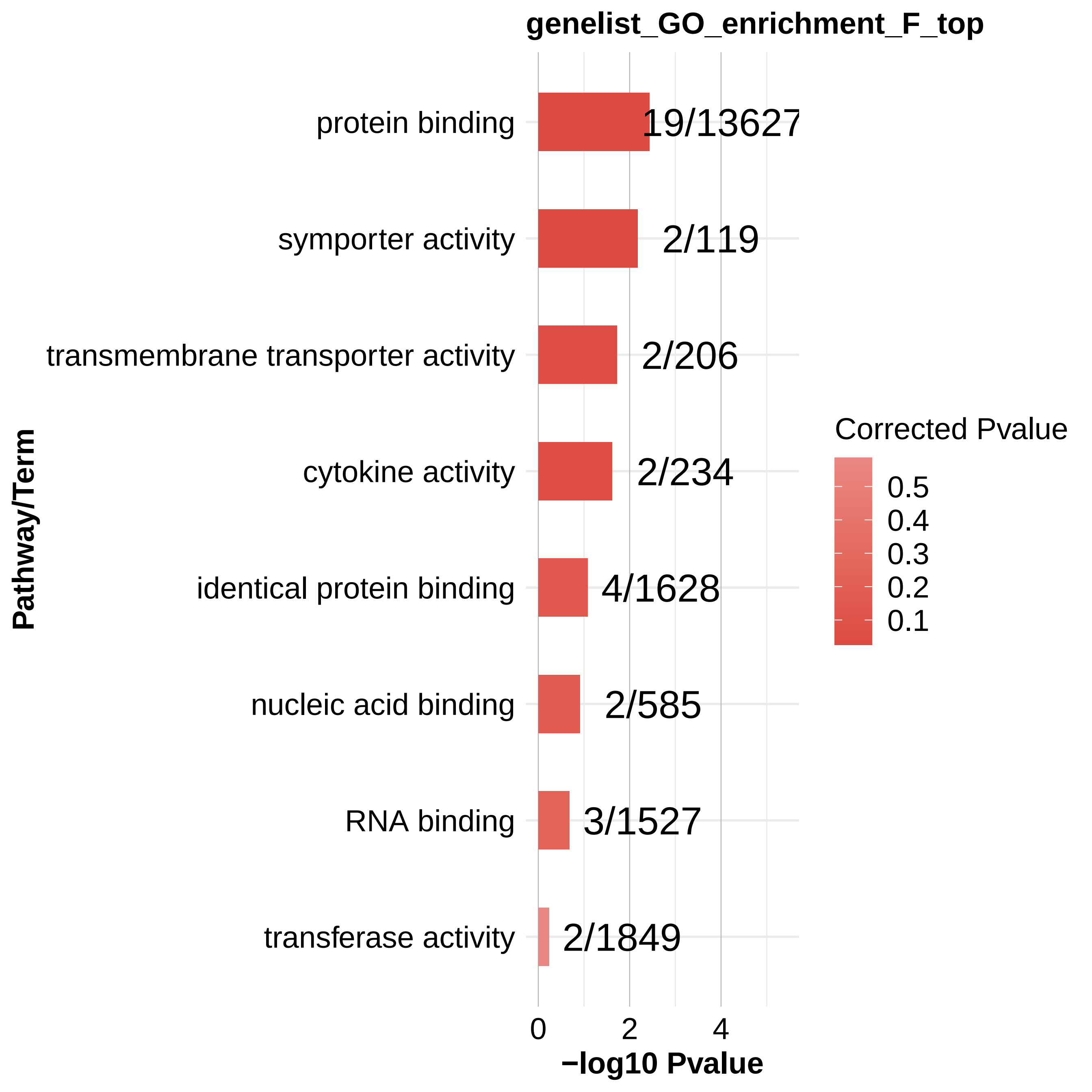

genelist_GO_enrichment_F_top
protein binding
symporter activity
transmembrane transporter activity
cytokine activity
identical protein binding
nucleic acid binding
RNA binding
transferase activity
19/13627
2/119
2/206
2/234
4/1628
2/585
3/1527
2/1849
Pathway/Term
0
2
4
−log10 Pvalue
Corrected Pvalue
0.5
0.4
0.3
0.2
0.1
