# Supplementary material for: Comprehensive and Combined Omics Analysis Reveals Immune–Infiltration‐Associated Alternative Splicing and RBP Regulators Contributing to Ischemia–Reperfusion Injury in Liver Transplantation
Source: Int J Genomics. 2026 Sep 21;2026:4835678. doi: 10.1155/ijog/4835678 (PMC13592132; doi:10.1155/ijog/4835678)
Supplement: Supplementary file 2 [file IJOG-2026-4835678-s019.zip › Supplementary_Enrichment_Results/genelist_KEGG_analysis/genelist_KEGG_pathway.html]

KEGG pathway enrichment analyze result


# KEGG pathway enrichment analyze result


| Term | Database | ID | Input number (15) | Background number (7854) | P-Value | Corrected P-Value | Input | Hyperlink | img |
| --- | --- | --- | --- | --- | --- | --- | --- | --- | --- |
| Vitamin digestion and absorption | KEGG PATHWAY | hsa04977 | 1 (6.67%) | 24 (0.31%) | 0.0449081074908 | 0.293356045734 | SLC5A6 | web link | no local link |
| Kaposi sarcoma-associated herpesvirus infection | KEGG PATHWAY | hsa05167 | 2 (13.33%) | 186 (2.37%) | 0.0478497129188 | 0.293356045734 | RCAN1  CXCL2 | web link | no local link |
| Biosynthesis of unsaturated fatty acids | KEGG PATHWAY | hsa01040 | 1 (6.67%) | 27 (0.34%) | 0.0503873311713 | 0.293356045734 | FADS1 | web link | no local link |
| Nicotinate and nicotinamide metabolism | KEGG PATHWAY | hsa00760 | 1 (6.67%) | 30 (0.38%) | 0.0558372056777 | 0.293356045734 | PNP | web link | no local link |
| Legionellosis | KEGG PATHWAY | hsa05134 | 1 (6.67%) | 55 (0.70%) | 0.100130867251 | 0.293356045734 | CXCL2 | web link | no local link |
| Fatty acid metabolism | KEGG PATHWAY | hsa01212 | 1 (6.67%) | 57 (0.73%) | 0.103589238904 | 0.293356045734 | FADS1 | web link | no local link |
| Pyrimidine metabolism | KEGG PATHWAY | hsa00240 | 1 (6.67%) | 57 (0.73%) | 0.103589238904 | 0.293356045734 | PNP | web link | no local link |
| Cytokine-cytokine receptor interaction | KEGG PATHWAY | hsa04060 | 2 (13.33%) | 294 (3.74%) | 0.106475410895 | 0.293356045734 | CXCL2  IL32 | web link | no local link |
| Epithelial cell signaling in Helicobacter pylori infection | KEGG PATHWAY | hsa05120 | 1 (6.67%) | 70 (0.89%) | 0.125768043952 | 0.293356045734 | CXCL2 | web link | no local link |
| Bile secretion | KEGG PATHWAY | hsa04976 | 1 (6.67%) | 72 (0.92%) | 0.12913435565 | 0.293356045734 | SLC22A7 | web link | no local link |
| PPAR signaling pathway | KEGG PATHWAY | hsa03320 | 1 (6.67%) | 76 (0.97%) | 0.135830715696 | 0.293356045734 | PLIN5 | web link | no local link |
| Complement and coagulation cascades | KEGG PATHWAY | hsa04610 | 1 (6.67%) | 79 (1.01%) | 0.140821414088 | 0.293356045734 | A2M | web link | no local link |
| Salmonella infection | KEGG PATHWAY | hsa05132 | 1 (6.67%) | 83 (1.06%) | 0.147433840557 | 0.293356045734 | CXCL2 | web link | no local link |
| GABAergic synapse | KEGG PATHWAY | hsa04727 | 1 (6.67%) | 89 (1.13%) | 0.157263475053 | 0.293356045734 | SLC38A2 | web link | no local link |
| Protein digestion and absorption | KEGG PATHWAY | hsa04974 | 1 (6.67%) | 90 (1.15%) | 0.158891427129 | 0.293356045734 | SLC38A2 | web link | no local link |
| IL-17 signaling pathway | KEGG PATHWAY | hsa04657 | 1 (6.67%) | 93 (1.18%) | 0.163757689932 | 0.293356045734 | CXCL2 | web link | no local link |
| Viral protein interaction with cytokine and cytokine receptor | KEGG PATHWAY | hsa04061 | 1 (6.67%) | 100 (1.27%) | 0.175010303247 | 0.293356045734 | CXCL2 | web link | no local link |
| NF-kappa B signaling pathway | KEGG PATHWAY | hsa04064 | 1 (6.67%) | 100 (1.27%) | 0.175010303247 | 0.293356045734 | CXCL2 | web link | no local link |
| Ribosome biogenesis in eukaryotes | KEGG PATHWAY | hsa03008 | 1 (6.67%) | 102 (1.30%) | 0.178199282483 | 0.293356045734 | NOP58 | web link | no local link |
| TNF signaling pathway | KEGG PATHWAY | hsa04668 | 1 (6.67%) | 112 (1.43%) | 0.193972326844 | 0.293356045734 | CXCL2 | web link | no local link |
| Glutamatergic synapse | KEGG PATHWAY | hsa04724 | 1 (6.67%) | 114 (1.45%) | 0.197092833696 | 0.293356045734 | SLC38A2 | web link | no local link |
| Thyroid hormone signaling pathway | KEGG PATHWAY | hsa04919 | 1 (6.67%) | 119 (1.52%) | 0.20484484042 | 0.293356045734 | RCAN1 | web link | no local link |
| Autophagy - animal | KEGG PATHWAY | hsa04140 | 1 (6.67%) | 128 (1.63%) | 0.218622697257 | 0.293356045734 | VMP1 | web link | no local link |
| Purine metabolism | KEGG PATHWAY | hsa00230 | 1 (6.67%) | 130 (1.66%) | 0.221654030127 | 0.293356045734 | PNP | web link | no local link |
| Spliceosome | KEGG PATHWAY | hsa03040 | 1 (6.67%) | 135 (1.72%) | 0.22918441073 | 0.293356045734 | SRSF3 | web link | no local link |
| Oxytocin signaling pathway | KEGG PATHWAY | hsa04921 | 1 (6.67%) | 153 (1.95%) | 0.255734478107 | 0.314750126901 | RCAN1 | web link | no local link |
| NOD-like receptor signaling pathway | KEGG PATHWAY | hsa04621 | 1 (6.67%) | 178 (2.27%) | 0.291195883644 | 0.343954867136 | CXCL2 | web link | no local link |
| Chemokine signaling pathway | KEGG PATHWAY | hsa04062 | 1 (6.67%) | 190 (2.42%) | 0.307651321397 | 0.343954867136 | CXCL2 | web link | no local link |
| Huntington disease | KEGG PATHWAY | hsa05016 | 1 (6.67%) | 193 (2.46%) | 0.311709098342 | 0.343954867136 | TGM2 | web link | no local link |
| PI3K-Akt signaling pathway | KEGG PATHWAY | hsa04151 | 1 (6.67%) | 354 (4.51%) | 0.499641704583 | 0.532951151555 | CDC37 | web link | no local link |
| Herpes simplex virus 1 infection | KEGG PATHWAY | hsa05168 | 1 (6.67%) | 492 (6.26%) | 0.621395971874 | 0.641441003225 | SRSF3 | web link | no local link |
| Metabolic pathways | KEGG PATHWAY | hsa01100 | 2 (13.33%) | 1432 (18.23%) | 0.788120227393 | 0.788120227393 | FADS1  PNP | web link | no local link |
