# Supplementary material for: Comprehensive and Combined Omics Analysis Reveals Immune–Infiltration‐Associated Alternative Splicing and RBP Regulators Contributing to Ischemia–Reperfusion Injury in Liver Transplantation
Source: Int J Genomics. 2026 Sep 21;2026:4835678. doi: 10.1155/ijog/4835678 (PMC13592132; doi:10.1155/ijog/4835678)
Supplement: Supplementary file 2 [file IJOG-2026-4835678-s019.zip › Supplementary_Enrichment_Results/genelist_KEGG_analysis/genelist_KEGG_top.pdf]

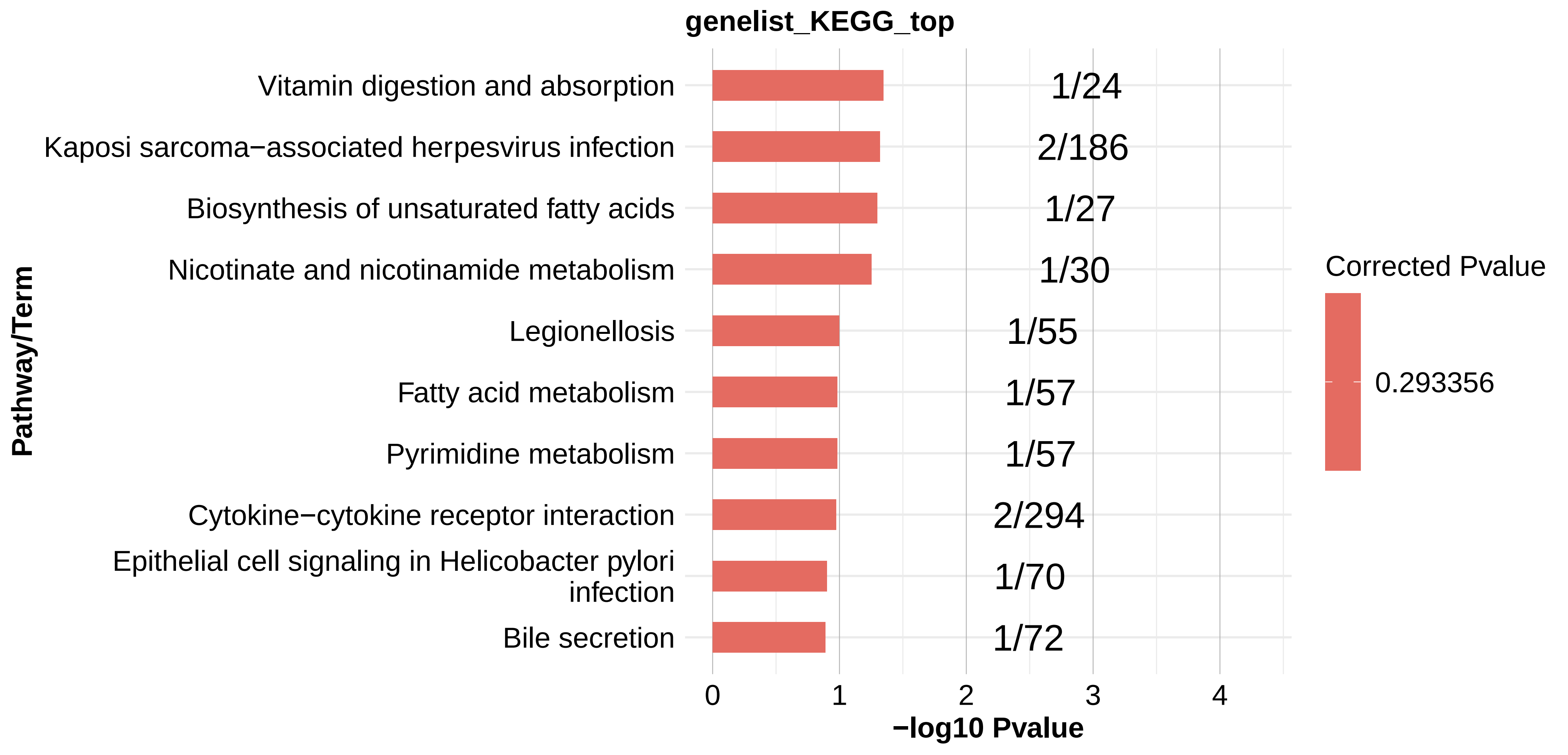
genelist_KEGG_top
Pathway/Term
Vitamin digestion and absorption
Kaposi sarcoma−associated herpesvirus infection
Biosynthesis of unsaturated fatty acids
Nicotinate and nicotinamide metabolism
Legionellosis
Fatty acid metabolism
Pyrimidine metabolism
Cytokine−cytokine receptor interaction
Epithelial cell signaling in Helicobacter pylori infection
Bile secretion
1/24
2/186
1/27
1/30
1/55
1/57
1/57
2/294
1/70
1/72
0
1
2
3
4
−log10 Pvalue
Corrected Pvalue
0.293356
