# Supplementary material for: Comprehensive and Combined Omics Analysis Reveals Immune–Infiltration‐Associated Alternative Splicing and RBP Regulators Contributing to Ischemia–Reperfusion Injury in Liver Transplantation
Source: Int J Genomics. 2026 Sep 21;2026:4835678. doi: 10.1155/ijog/4835678 (PMC13592132; doi:10.1155/ijog/4835678)
Supplement: Supplementary file 2 [file IJOG-2026-4835678-s019.zip › Supplementary_Enrichment_Results/IRI_1_spec.txt.anno.gene_GO_analysis/IRI_1_spec.txt.anno.gene_GO_enrichment_C_top.pdf]

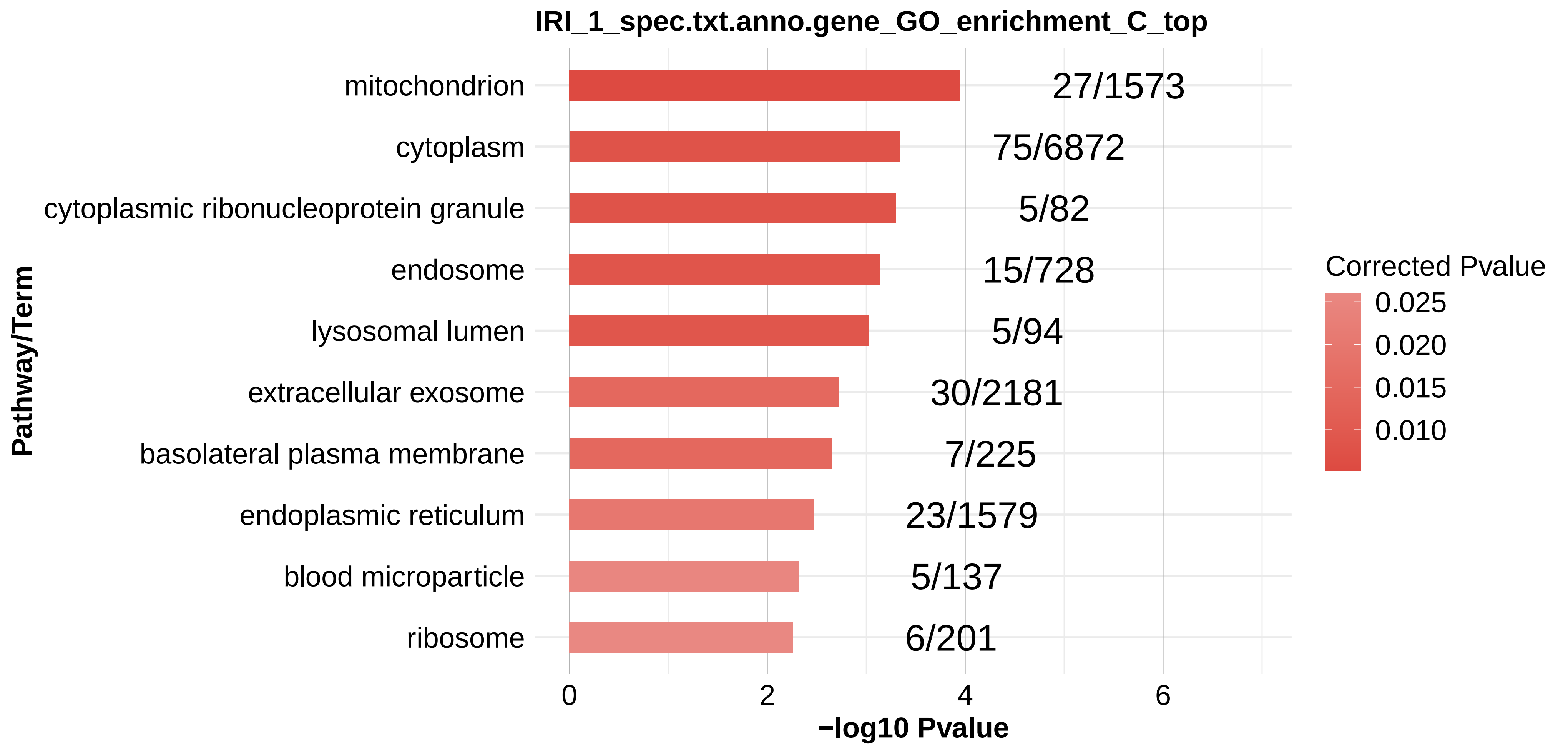

IRI_1_spec.txt.anno.gene_GO_enrichment_C_top
Pathway/Term
mitochondrion
cytoplasm
cytoplasmic ribonucleoprotein granule
endosome
lysosomal lumen
extracellular exosome
basolateral plasma membrane
endoplasmic reticulum
blood microparticle
ribosome
27/1573
75/6872
5/82
15/728
5/94
30/2181
7/225
23/1579
5/137
6/201
0
2
4
6
−log10 Pvalue
Corrected Pvalue
0.025
0.020
0.015
0.010
