# Supplementary material for: Comprehensive and Combined Omics Analysis Reveals Immune–Infiltration‐Associated Alternative Splicing and RBP Regulators Contributing to Ischemia–Reperfusion Injury in Liver Transplantation
Source: Int J Genomics. 2026 Sep 21;2026:4835678. doi: 10.1155/ijog/4835678 (PMC13592132; doi:10.1155/ijog/4835678)
Supplement: Supplementary file 2 [file IJOG-2026-4835678-s019.zip › Supplementary_Enrichment_Results/IRI_1_spec.txt.anno.gene_GO_analysis/IRI_1_spec.txt.anno.gene_GO_enrichment_P_top.pdf]

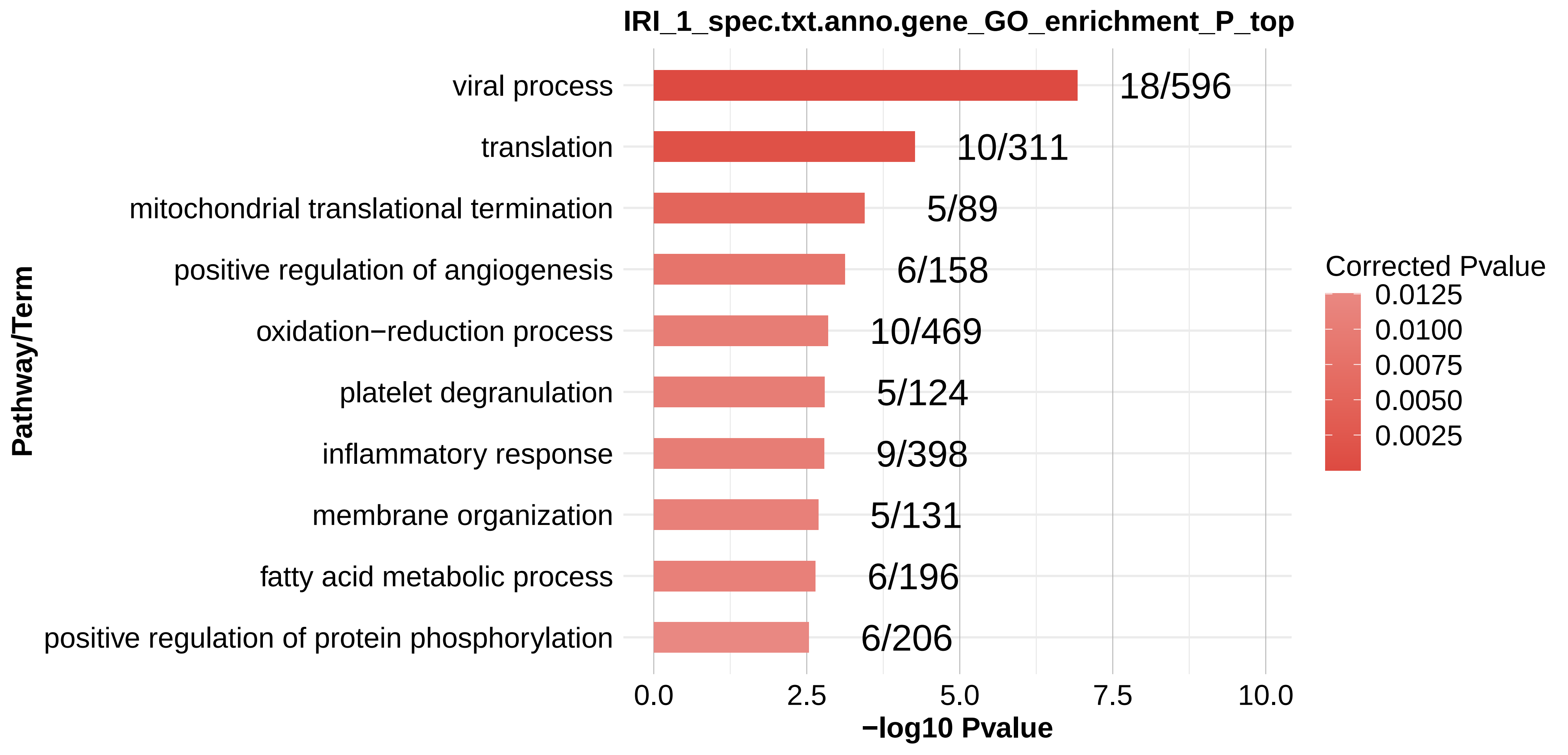

IRI_1_spec.txt.anno.gene_GO_enrichment_P_top
Pathway/Term
viral process
translation
mitochondrial translational termination
positive regulation of angiogenesis
oxidation−reduction process
platelet degranulation
inflammatory response
membrane organization
fatty acid metabolic process
positive regulation of protein phosphorylation
18/596
10/311
5/89
6/158
10/469
5/124
9/398
5/131
6/196
6/206
0.0
2.5
5.0
7.5
10.0
−log10 Pvalue
Corrected Pvalue
0.0125
0.0100
0.0075
0.0050
0.0025
