# Supplementary material for: Comprehensive and Combined Omics Analysis Reveals Immune–Infiltration‐Associated Alternative Splicing and RBP Regulators Contributing to Ischemia–Reperfusion Injury in Liver Transplantation
Source: Int J Genomics. 2026 Sep 21;2026:4835678. doi: 10.1155/ijog/4835678 (PMC13592132; doi:10.1155/ijog/4835678)
Supplement: Supplementary file 2 [file IJOG-2026-4835678-s019.zip › Supplementary_Enrichment_Results/IRI_1_spec.txt.anno.gene_GO_analysis/IRI_1_spec.txt.anno.gene_GO_enrichment_web_C.html]

GO enrichment analyze


# GO enrichment analyze result


| Term | Database | ID | Input number (148) | Background number (18569) | P-Value | Corrected P-Value | Input | Hyperlink |
| --- | --- | --- | --- | --- | --- | --- | --- | --- |
| mitochondrion | Gene Ontology | GO:0005739 | 27 (18.24%) | 1573 (8.47%) | 0.000111901453765 | 0.00525936832696 | TGM2  HSPA9  CYP2D6  C1orf43  MRPS11  SLC25A18  FKBP8  STAT3  MFF  AASS  MRPL55  MPST  PITRM1  ENY2  IFI27  MRPL11  TARS2  GRAMD4  BPHL  FADS1  MTFR1L  MRRF  DHODH  GLYATL1  MRPL42  NEU4  ECHDC2 | web link |
| cytoplasm | Gene Ontology | GO:0005737 | 75 (50.68%) | 6872 (37.01%) | 0.000452921208878 | 0.00784692679472 | USP8  LONP2  TGM2  ABLIM3  TMEM134  ZNF207  HSPA9  TPD52L1  GNE  KAT5  ZFAND5  AKR1A1  RNH1  CHI3L1  DNM2  CYP2A7  ZC3H12A  KRT8  CTH  AKR1C2  XBP1  GPS1  PVR  CDV3  HYAL1  CYP2D6  AASS  NFE2L2  CENATAC  HNRNPU  CDC37  CAST  CSNK1D  NMD3  MED15  MBNL3  TUT7  BLNK  IFITM3  AFM  TFR2  TARS2  HAL  NXF1  DPH1  EPN1  TSC22D3  GMPPA  NPHP3-ACAD11  PER1  MPST  ATN1  NUMB  CTBP1  STAT3  MLX  SULT1A2  HTATIP2  RCC1  CSNK1A1  EIF2AK1  GADD45G  DHODH  ACOX1  CCNY  A1CF  DDX5  CYP4F12  EMP2  KPNA1  FAM20A  NEU4  BAIAP2  ATF6  EWSR1 | web link |
| cytoplasmic ribonucleoprotein granule | Gene Ontology | GO:0036464 | 5 (3.38%) | 82 (0.44%) | 0.000500867667748 | 0.00784692679472 | NFKBIZ  ZC3H12A  CHTOP  HNRNPU  BLNK | web link |
| endosome | Gene Ontology | GO:0005768 | 15 (10.14%) | 728 (3.92%) | 0.000719609645179 | 0.00845541333086 | IFITM3  MRC1  USP8  GCGR  SLC39A14  WASHC3  EPN1  GOLIM4  SMPD1  NUMB  DNM2  APOA5  TMEM175  IL15RA  RAB17 | web link |
| lysosomal lumen | Gene Ontology | GO:0043202 | 5 (3.38%) | 94 (0.51%) | 0.000933577671182 | 0.00877563010911 | NEU4  HYAL1  SMPD1  CHID1  ACP2 | web link |
| extracellular exosome | Gene Ontology | GO:0070062 | 30 (20.27%) | 2181 (11.75%) | 0.00190608833405 | 0.0148108979412 | TGM2  ACP2  HSPA9  QSOX1  PROS1  AKR1A1  SMPD1  RNH1  CHI3L1  DNM2  KRT8  CTH  A2M  SERPINA3  CDC37  MPST  LSR  RAB17  AFM  SCAMP3  GMPPA  FAM20A  HYAL1  MASP2  DDX5  BAIAP2  CHID1  TTC38  RALB  RPS2 | web link |
| basolateral plasma membrane | Gene Ontology | GO:0016323 | 7 (4.73%) | 225 (1.21%) | 0.00220587841678 | 0.0148108979412 | NUMB  SLC39A14  SLC22A7  RAB17  SLC5A6  ABCA8  ABCC3 | web link |
| endoplasmic reticulum | Gene Ontology | GO:0005783 | 23 (15.54%) | 1579 (8.50%) | 0.00341147308822 | 0.0200424043933 | TGM2  IFI27  PCSK6  BCHE  CHI3L1  ZC3H12A  FKBP8  XBP1  CYP2D6  CYP4F12  CAST  GRAMD4  RPS24  CYP2A7  MIA3  FADS1  FAM20A  FNDC4  NEU4  ATF6  IL15RA  A1CF  ABCA8 | web link |
| blood microparticle | Gene Ontology | GO:0072562 | 5 (3.38%) | 137 (0.74%) | 0.00483956140498 | 0.0252732651149 | AFM  BCHE  SERPINA3  PROS1  A2M | web link |
| ribosome | Gene Ontology | GO:0005840 | 6 (4.05%) | 201 (1.08%) | 0.00552439378622 | 0.0259646507953 | MRPL11  RPS24  MRPL55  MRPL42  RPS2  MRPS11 | web link |
| glutamatergic synapse | Gene Ontology | GO:0098978 | 8 (5.41%) | 349 (1.88%) | 0.00696689493957 | 0.0292707054293 | USP8  STAT3  ABLIM3  NUMB  CTBP1  KPNA1  DNM2  BAIAP2 | web link |
| early endosome | Gene Ontology | GO:0005769 | 7 (4.73%) | 285 (1.53%) | 0.00795275795301 | 0.0292707054293 | USP8  WASHC3  CD4  RAB17  NUMB  APOA5  PICALM | web link |
| lysosome | Gene Ontology | GO:0005764 | 9 (6.08%) | 434 (2.34%) | 0.00809615256556 | 0.0292707054293 | IFITM3  ACP2  SLC39A14  SLC17A4  HYAL1  SMPD1  NEU4  CHID1  TMEM175 | web link |
| mitochondrial membrane | Gene Ontology | GO:0031966 | 5 (3.38%) | 172 (0.93%) | 0.0122994574653 | 0.0412910357763 | NEU4  IFI27  GRAMD4  FKBP8  DNM2 | web link |
| nuclear speck | Gene Ontology | GO:0016607 | 8 (5.41%) | 410 (2.21%) | 0.0171287091082 | 0.0536699552056 | NXF1  CHTOP  HNRNPU  SNRPA1  NFKBIZ  SNRNP70  RBM39  CSNK1A1 | web link |
| nucleoplasm | Gene Ontology | GO:0005654 | 41 (27.70%) | 3824 (20.59%) | 0.0231787249769 | 0.0680875046197 | HNRNPU  RPS2  ZC3H12A  KRT8  NCOR2  XBP1  GPS1  STAT3  NFE2L2  SNRPA1  ZCCHC9  MED15  ENY2  MBNL3  RBM39  TUT7  ZNF207  RPS24  NXF1  DPH1  CHTOP  PER1  CSNK1D  ATN1  BAIAP2  NMD3  MLX  NFIC  RCC1  NFIA  SNRNP70  DHODH  KAT5  A1CF  DDX5  PWWP3A  KPNA1  CTBP1  ATF6  EWSR1  RNH1 | web link |
| apical plasma membrane | Gene Ontology | GO:0016324 | 7 (4.73%) | 359 (1.93%) | 0.0251404241077 | 0.0695058784155 | SLC39A14  CYP4F12  SLC17A4  AKR1A1  EMP2  SLC5A6  RAB17 | web link |
| mitochondrial inner membrane | Gene Ontology | GO:0005743 | 8 (5.41%) | 462 (2.49%) | 0.0319332219972 | 0.0833811907704 | MRPL11  STAT3  MRPL42  DHODH  MRPL55  MRPS11  SLC25A18  NEU4 | web link |
| intracellular membrane-bounded organelle | Gene Ontology | GO:0043231 | 13 (8.78%) | 998 (5.37%) | 0.0556831226865 | 0.137742461382 | NEU4  SCAMP3  CYP2D6  CYP4F12  QSOX1  EPN1  AASS  CYP2A7  PICALM  MIA3  FADS1  ABCA8  AGFG1 | web link |
| perinuclear region of cytoplasm | Gene Ontology | GO:0048471 | 10 (6.76%) | 725 (3.90%) | 0.0648247813529 | 0.152338236179 | IFITM3  TGM2  TMEM134  TPD52L1  KAT5  CSNK1D  ATN1  CHI3L1  DNM2  PICALM | web link |
| postsynaptic density | Gene Ontology | GO:0014069 | 5 (3.38%) | 280 (1.51%) | 0.0736880146514 | 0.16126823051 | USP8  BAIAP2  STAT3  KPNA1  DNM2 | web link |
| endoplasmic reticulum membrane | Gene Ontology | GO:0005789 | 13 (8.78%) | 1048 (5.64%) | 0.0754872568347 | 0.16126823051 | CYP2D6  CYP4F12  IFI27  PROS1  CD4  NEU4  CYP2A7  MIA3  GRAMD4  FADS1  ATF6  IL15RA  XBP1 | web link |
| endosome membrane | Gene Ontology | GO:0010008 | 5 (3.38%) | 288 (1.55%) | 0.0808659927894 | 0.165247898309 | USP8  MRC1  GOLIM4  TMEM175  NUMB | web link |
| mitochondrial matrix | Gene Ontology | GO:0005759 | 6 (4.05%) | 383 (2.06%) | 0.0863776202092 | 0.16915617291 | TARS2  MRRF  AASS  HSPA9  MPST  PITRM1 | web link |
| endoplasmic reticulum lumen | Gene Ontology | GO:0005788 | 5 (3.38%) | 305 (1.64%) | 0.0973100959928 | 0.182942980466 | CD4  APOA5  QSOX1  BCHE  MIA3 | web link |
| focal adhesion | Gene Ontology | GO:0005925 | 6 (4.05%) | 419 (2.26%) | 0.11876583453 | 0.20696589535 | TGM2  PVR  HSPA9  RPS2  NUMB  DNM2 | web link |
| cell surface | Gene Ontology | GO:0009986 | 8 (5.41%) | 615 (3.31%) | 0.118895301584 | 0.20696589535 | MRC1  PVR  PCSK6  HNRNPU  CD4  EMP2  IL15RA  PICALM | web link |
| cytosol | Gene Ontology | GO:0005829 | 49 (33.11%) | 5296 (28.52%) | 0.125880908767 | 0.210152670062 | USP8  RBCK1  TGM2  TMEM134  GNE  RPS24  AKR1A1  RNH1  DNM2  KRT8  FKBP8  A2M  AKR1C2  XBP1  GPS1  RPS2  STAT3  AASS  NFE2L2  CSNK1D  CDC37  CAST  MPST  EMP2  TUT7  BLNK  LONP2  GSPT1  HAL  NXF1  TSC22D3  EPN1  SULT1A2  PER1  CTH  CDV3  MLX  HTATIP2  DPH1  CSNK1A1  DHODH  ACOX1  DDX5  PWWP3A  KPNA1  BAIAP2  ATF6  PICALM  AGFG1 | web link |
| centrosome | Gene Ontology | GO:0005813 | 7 (4.73%) | 528 (2.84%) | 0.129668668761 | 0.210152670062 | CSNK1A1  NFE2L2  CENATAC  HNRNPU  HSPB11  CSNK1D  DNM2 | web link |
| nucleus | Gene Ontology | GO:0005634 | 59 (39.86%) | 6548 (35.26%) | 0.138135812681 | 0.2164127732 | USP8  TGM2  CCNY  IFI27  HNRNPU  NFKBIZ  RPS2  DNM2  ZC3H12A  KRT8  NCOR2  XBP1  GPS1  SERPINA3  STAT3  AASS  NFE2L2  SNRPA1  GADD45G  ZCCHC9  MED15  ENY2  EMP2  MBNL3  RBM39  HSPA9  LONP2  ZNF207  RPS24  NXF1  RCC1  TSC22D3  CHTOP  EPN1  PER1  CSNK1D  ATN1  NUMB  FADS1  NMD3  MLX  HTATIP2  NFIC  DPH1  NFIA  SNRNP70  CSNK1A1  KAT5  EWSR1  DDX5  PWWP3A  KPNA1  CTBP1  CHID1  ATF6  IL15RA  A1CF  PICALM  AGFG1 | web link |
| membrane | Gene Ontology | GO:0016020 | 74 (50.00%) | 8478 (45.66%) | 0.162987939121 | 0.245252398868 | USP8  LONP2  TGM2  NUMB  TMEM134  FNDC4  IFI27  SLC25A18  OCEL1  RPS24  CD4  C1orf43  BCHE  RPS2  SLC5A6  DNM2  ZC3H12A  TMEM175  FKBP8  NCOR2  XBP1  MRC1  PVR  NMD3  ACP2  CYP2D6  DDX5  CYP4F12  HNRNPU  CAST  CSNK1D  ST7  LSR  MED15  GRAMD4  ADGRA3  SMIM19  RAB17  BLNK  IFITM3  TFR2  QSOX1  SCAMP3  PCSK6  EPN1  GOLIM4  CYP2A7  MIA3  BAIAP2  FADS1  GCGR  MFF  HTATIP2  CSNK1A1  SLC39A14  SLC17A4  DHODH  ACOX1  CCNY  EWSR1  MRPL42  AKR1A1  SLC22A7  EMP2  ABCA8  GGT5  NEU4  SMPD1  CHID1  ATF6  IL15RA  RALB  PICALM  ABCC3 | web link |
| extracellular space | Gene Ontology | GO:0005615 | 19 (12.84%) | 1880 (10.12%) | 0.166980356676 | 0.245252398868 | AFM  PVR  SERPINA3  FNDC4  FAM20A  HYAL1  QSOX1  PROS1  PCSK6  THPO  SMPD1  AKR1A1  BCHE  MASP2  CHI3L1  APOA5  CHID1  IL15RA  A2M | web link |
| cytoplasmic vesicle | Gene Ontology | GO:0031410 | 9 (6.08%) | 798 (4.30%) | 0.187132298669 | 0.266521758711 | TFR2  MFF  HYAL1  EMP2  NUMB  DNM2  IL15RA  PICALM  AGFG1 | web link |
| Golgi membrane | Gene Ontology | GO:0000139 | 7 (4.73%) | 620 (3.34%) | 0.226052808375 | 0.312484764519 | QSOX1  PROS1  GOLIM4  EMP2  DNM2  ATF6  IL15RA | web link |
| protein-containing complex | Gene Ontology | GO:0032991 | 8 (5.41%) | 740 (3.99%) | 0.238395234177 | 0.320130743038 | IFITM3  RCC1  MFF  FKBP8  HNRNPU  DNM2  ZC3H12A  RBM39 | web link |
| Golgi apparatus | Gene Ontology | GO:0005794 | 13 (8.78%) | 1366 (7.36%) | 0.293774551027 | 0.383538997174 | QSOX1  NFE2L2  FAM20A  BAIAP2  CSNK1D  GOLIM4  C1orf43  EMP2  DNM2  APOA5  ATF6  IL15RA  PICALM | web link |
| chromosome | Gene Ontology | GO:0005694 | 6 (4.05%) | 578 (3.11%) | 0.314021131906 | 0.398891708096 | RCC1  TGM2  CSNK1A1  ZNF207  HNRNPU  CFDP1 | web link |
| nucleolus | Gene Ontology | GO:0005730 | 8 (5.41%) | 848 (4.57%) | 0.364346474845 | 0.450639060993 | ZNF207  NMD3  HSPA9  CHTOP  KAT5  DDX5  ZCCHC9  EWSR1 | web link |
| chromatin | Gene Ontology | GO:0000785 | 9 (6.08%) | 980 (5.28%) | 0.380173997382 | 0.458158407102 | NFIC  RCC1  NFIA  STAT3  NFE2L2  TGM2  MLX  ATF6  NCOR2 | web link |
| integral component of membrane | Gene Ontology | GO:0016021 | 44 (29.73%) | 5472 (29.47%) | 0.503299249346 | 0.591376617981 | ACP2  FNDC4  SMIM19  SLC25A18  CYP2D6  CD4  C1orf43  SMPD1  SLC5A6  OCEL1  TMEM175  FKBP8  XBP1  MRC1  PVR  TMEM134  MFF  CYP4F12  HNRNPU  ST7  LSR  GRAMD4  IFI27  IFITM3  TFR2  QSOX1  SCAMP3  GOLIM4  CYP2A7  MIA3  GGT5  FADS1  GCGR  SLC39A14  SLC17A4  DHODH  MRPL42  ADGRA3  SLC22A7  EMP2  ATF6  IL15RA  ABCA8  ABCC3 | web link |
| extracellular region | Gene Ontology | GO:0005576 | 19 (12.84%) | 2461 (13.25%) | 0.596235520272 | 0.683489498848 | AFM  TGM2  PVR  FNDC4  PCSK6  HYAL1  QSOX1  PROS1  MASP2  THPO  BCHE  SERPINA3  SMPD1  CHI3L1  FAM20A  APOA5  CHID1  IL15RA  A2M | web link |
| plasma membrane | Gene Ontology | GO:0005886 | 40 (27.03%) | 5167 (27.83%) | 0.616943403565 | 0.690389046846 | USP8  TGM2  PCSK6  CD4  AKR1A1  SMPD1  SLC5A6  DNM2  MRC1  PVR  CDV3  SLC39A14  STAT3  FNDC4  NFE2L2  CSNK1D  LSR  RAB17  BLNK  IFITM3  TFR2  PROS1  EPN1  NUMB  GGT5  GCGR  SLC17A4  CCNY  RALB  MRPL42  ADGRA3  SLC22A7  EMP2  PICALM  NEU4  BAIAP2  IL15RA  EWSR1  ABCA8  ABCC3 | web link |
| cell junction | Gene Ontology | GO:0030054 | 6 (4.05%) | 922 (4.97%) | 0.749523806315 | 0.819246951088 | DPH1  NFIA  MFF  MPST  ATN1  DNM2 | web link |
| synapse | Gene Ontology | GO:0045202 | 5 (3.38%) | 806 (4.34%) | 0.774992454092 | 0.827832848689 | MPST  AKR1A1  MFF  ABLIM3  DNM2 | web link |
| cytoskeleton | Gene Ontology | GO:0005856 | 9 (6.08%) | 1406 (7.57%) | 0.797418188002 | 0.832858996358 | ZNF207  CSNK1A1  CENATAC  HNRNPU  NPHP3-ACAD11  ZC3H12A  CSNK1D  DNM2  BAIAP2 | web link |
| integral component of plasma membrane | Gene Ontology | GO:0005887 | 8 (5.41%) | 1398 (7.53%) | 0.876540625427 | 0.895595856414 | MRC1  TFR2  SLC39A14  SLC17A4  CD4  SLC22A7  SLC5A6  GCGR | web link |
| cell projection | Gene Ontology | GO:0042995 | 6 (4.05%) | 1142 (6.15%) | 0.898711289703 | 0.898711289703 | CSNK1A1  HSPB11  DNM2  NEU4  BAIAP2  RAB17 | web link |
