# Supplementary material for: Comprehensive and Combined Omics Analysis Reveals Immune–Infiltration‐Associated Alternative Splicing and RBP Regulators Contributing to Ischemia–Reperfusion Injury in Liver Transplantation
Source: Int J Genomics. 2026 Sep 21;2026:4835678. doi: 10.1155/ijog/4835678 (PMC13592132; doi:10.1155/ijog/4835678)
Supplement: Supplementary file 2 [file IJOG-2026-4835678-s019.zip › Supplementary_Enrichment_Results/IRI_1_spec.txt.anno.gene_GO_analysis/IRI_1_spec.txt.anno.gene_GO_enrichment_web_F.html]

GO enrichment analyze


# GO enrichment analyze result


| Term | Database | ID | Input number (145) | Background number (18273) | P-Value | Corrected P-Value | Input | Hyperlink |
| --- | --- | --- | --- | --- | --- | --- | --- | --- |
| RNA binding | Gene Ontology | GO:0003723 | 27 (18.62%) | 1527 (8.36%) | 6.18153029488e-05 | 0.00210172030026 | MRPL11  HSPA9  RPS24  SNRPA1  RPS2  MRPS11  ZC3H12A  NMD3  MRPL42  HNRNPU  CAST  ZCCHC9  SNRNP70  RBM39  TUT7  ZNF207  GSPT1  NXF1  PRRC2B  CHTOP  ANKHD1-EIF4EBP3  MBNL3  RSRC2  EWSR1  DDX5  A1CF  AGFG1 | web link |
| enzyme binding | Gene Ontology | GO:0019899 | 10 (6.90%) | 395 (2.16%) | 0.00123348260049 | 0.0168738027942 | LONP2  HSPA9  CD4  DDX5  BCHE  RPS2  DNM2  APOA5  A2M  BLNK | web link |
| structural constituent of ribosome | Gene Ontology | GO:0003735 | 6 (4.14%) | 162 (0.89%) | 0.00186801807575 | 0.0168738027942 | MRPL11  RPS24  MRPL55  MRPL42  RPS2  MRPS11 | web link |
| protein binding | Gene Ontology | GO:0005515 | 123 (84.83%) | 13627 (74.57%) | 0.0019851532699 | 0.0168738027942 | WASHC3  TGM2  HSPA9  PCSK6  ZFAND5  CHI3L1  SLC25A18  TMEM175  FKBP8  GPS1  ACP2  SLC39A14  FNDC4  NFE2L2  GADD45G  MPST  PITRM1  MBNL3  IFI27  TUT7  BLNK  IFITM3  ZNF207  PRRC2B  TSC22D3  CHTOP  PER1  ABLIM3  ANKHD1-EIF4EBP3  FAM120AOS  HTATIP2  MRRF  DHODH  KAT5  MASP2  DDX5  KPNA1  CTBP1  AFM  PROS1  GSPT1  CCNY  NFKBIZ  AKR1A1  BCHE  RNH1  DNM2  APOA5  MFF  A2M  GRAMD4  RBM39  MRPL11  TFR2  TARS2  NXF1  MRC1  EPN1  NPHP3-ACAD11  CSNK1D  MIA3  CSNK1A1  CYP4F12  ADGRA3  SLC22A7  PWWP3A  RBCK1  BAIAP2  TTC38  IL15RA  RALB  AGFG1  TMEM134  SMIM19  SNRPA1  CD4  SMPD1  RPS2  KRT8  CTH  NCOR2  PVR  STAT3  CENATAC  HNRNPU  CDC37  CAST  ENY2  SNRNP70  RAB17  RCC1  GMPPA  SULT1A2  BPHL  TPD52L1  EIF2AK1  FAM20A  RSRC2  EMP2  NEU4  USP8  SLC5A6  MRPS11  GNE  ZC3H12A  XBP1  SERPINA3  ST7  MED15  LONP2  ATN1  NUMB  MLX  MTFR1L  NFIC  DPH1  NFIA  A1CF  HSPB11  CHID1  ATF6  EWSR1  PICALM | web link |
| oxidoreductase activity | Gene Ontology | GO:0016491 | 12 (8.28%) | 602 (3.29%) | 0.0030981260912 | 0.0210672574201 | ACOX1  CYP2D6  CYP4F12  DHODH  QSOX1  AASS  AKR1A1  CYP2A7  FADS1  CTBP1  HTATIP2  AKR1C2 | web link |
| protein kinase binding | Gene Ontology | GO:0019901 | 9 (6.21%) | 479 (2.62%) | 0.0143089888066 | 0.0804665729127 | STAT3  CCNY  CDC37  CD4  EMP2  DNM2  XBP1  IL15RA  BLNK | web link |
| transcription corepressor activity | Gene Ontology | GO:0003714 | 5 (3.45%) | 188 (1.03%) | 0.0171423560761 | 0.0804665729127 | AASS  CTBP1  ATN1  NCOR2  HNRNPU | web link |
| transcription factor binding | Gene Ontology | GO:0008134 | 7 (4.83%) | 340 (1.86%) | 0.0189333112736 | 0.0804665729127 | NFIA  STAT3  NFE2L2  HYAL1  PER1  CTBP1  MLX | web link |
| catalytic activity | Gene Ontology | GO:0003824 | 8 (5.52%) | 432 (2.36%) | 0.0220628999338 | 0.0827279610139 | HAL  HYAL1  AASS  DHODH  BCHE  GNE  CTH  ECHDC2 | web link |
| transmembrane transporter activity | Gene Ontology | GO:0022857 | 5 (3.45%) | 206 (1.13%) | 0.0243317532394 | 0.0827279610139 | SLC17A4  SLC22A7  ABCC3  SLC5A6  SLC25A18 | web link |
| mRNA binding | Gene Ontology | GO:0003729 | 5 (3.45%) | 213 (1.17%) | 0.0275821353528 | 0.0852538729085 | ZC3H12A  NXF1  SNRNP70  A1CF  RPS2 | web link |
| ubiquitin protein ligase binding | Gene Ontology | GO:0031625 | 6 (4.14%) | 298 (1.63%) | 0.0317436573825 | 0.0899403625838 | SCAMP3  HSPA9  PER1  ATF6  RALB  XBP1 | web link |
| cadherin binding | Gene Ontology | GO:0045296 | 6 (4.14%) | 316 (1.73%) | 0.0404092132242 | 0.105685634586 | USP8  CAST  CSNK1D  RPS2  NUMB  PICALM | web link |
| nucleic acid binding | Gene Ontology | GO:0003676 | 9 (6.21%) | 585 (3.20%) | 0.0437433249168 | 0.106233789084 | NXF1  EWSR1  DDX5  ZCCHC9  ANKHD1-EIF4EBP3  SNRNP70  RBM39  A1CF  TUT7 | web link |
| transcription regulatory region sequence-specific DNA binding | Gene Ontology | GO:0000976 | 5 (3.45%) | 248 (1.36%) | 0.0478930586892 | 0.108557599696 | NFE2L2  PER1  ATF6  STAT3  XBP1 | web link |
| chromatin binding | Gene Ontology | GO:0003682 | 7 (4.83%) | 455 (2.49%) | 0.0708508096498 | 0.150557970506 | RCC1  NFIA  HNRNPU  CTBP1  ENY2  ZC3H12A  NCOR2 | web link |
| protein homodimerization activity | Gene Ontology | GO:0042803 | 9 (6.21%) | 680 (3.72%) | 0.0921819001044 | 0.184363800209 | TARS2  STAT3  MFF  EIF2AK1  ACOX1  CD4  TPD52L1  MLX  XBP1 | web link |
| peptidase activity | Gene Ontology | GO:0008233 | 7 (4.83%) | 521 (2.85%) | 0.121275154053 | 0.226651855098 | LONP2  USP8  TGM2  GGT5  PCSK6  MASP2  PITRM1 | web link |
| hydrolase activity | Gene Ontology | GO:0016787 | 18 (12.41%) | 1700 (9.30%) | 0.126658389613 | 0.226651855098 | LONP2  USP8  TGM2  GNE  ACP2  PITRM1  HYAL1  DNM2  PCSK6  MASP2  ZC3H12A  DDX5  BCHE  BPHL  GGT5  NEU4  SMPD1  RALB | web link |
| ATPase activity | Gene Ontology | GO:0016887 | 5 (3.45%) | 349 (1.91%) | 0.144985276077 | 0.238867776687 | LONP2  DDX5  HSPA9  ABCA8  ABCC3 | web link |
| identical protein binding | Gene Ontology | GO:0042802 | 17 (11.72%) | 1628 (8.91%) | 0.147535979718 | 0.238867776687 | TGM2  STAT3  MFF  CTH  IFI27  BAIAP2  HNRNPU  MASP2  CD4  MPST  BCHE  RBCK1  TPD52L1  CTBP1  ATF6  FKBP8  EWSR1 | web link |
| GTP binding | Gene Ontology | GO:0005525 | 5 (3.45%) | 376 (2.06%) | 0.179519482744 | 0.277439200605 | GSPT1  TGM2  RALB  RAB17  DNM2 | web link |
| DNA-binding transcription activator activity, RNA polymerase II-specific | Gene Ontology | GO:0001228 | 5 (3.45%) | 446 (2.44%) | 0.280487013317 | 0.40086625218 | NFIC  NFE2L2  NFIA  ATF6  STAT3 | web link |
| DNA-binding transcription factor activity | Gene Ontology | GO:0003700 | 7 (4.83%) | 670 (3.67%) | 0.282964413304 | 0.40086625218 | NFIC  NFIA  STAT3  NFE2L2  MLX  ATF6  XBP1 | web link |
| nucleotide binding | Gene Ontology | GO:0000166 | 16 (11.03%) | 1828 (10.00%) | 0.378332117249 | 0.514531679459 | LONP2  TGM2  TARS2  CSNK1A1  EIF2AK1  HSPA9  GNE  GSPT1  HNRNPU  DDX5  CSNK1D  RAB17  DNM2  RALB  ABCA8  ABCC3 | web link |
| sequence-specific double-stranded DNA binding | Gene Ontology | GO:1990837 | 5 (3.45%) | 551 (3.02%) | 0.444853636582 | 0.581731678607 | NFIC  MLX  ATF6  HNRNPU  XBP1 | web link |
| zinc ion binding | Gene Ontology | GO:0008270 | 7 (4.83%) | 822 (4.50%) | 0.479246479838 | 0.603495567204 | AFM  ZCCHC9  CD4  ZFAND5  SMPD1  PITRM1  TUT7 | web link |
| transferase activity | Gene Ontology | GO:0016740 | 15 (10.34%) | 1849 (10.12%) | 0.504430414998 | 0.612522646784 | DPH1  TGM2  CSNK1A1  EIF2AK1  GLYATL1  GMPPA  KAT5  SULT1A2  MPST  CSNK1D  GGT5  FGGY  RBCK1  GNE  TUT7 | web link |
| RNA polymerase II cis-regulatory region sequence-specific DNA binding | Gene Ontology | GO:0000978 | 9 (6.21%) | 1169 (6.40%) | 0.586183857806 | 0.687250040186 | NFIC  NFIA  STAT3  NFE2L2  HNRNPU  PER1  MLX  ATF6  XBP1 | web link |
| ATP binding | Gene Ontology | GO:0005524 | 11 (7.59%) | 1476 (8.08%) | 0.630621810715 | 0.706316592129 | LONP2  TARS2  CSNK1A1  EIF2AK1  HSPA9  HNRNPU  DDX5  CSNK1D  GNE  ABCA8  ABCC3 | web link |
| kinase activity | Gene Ontology | GO:0016301 | 5 (3.45%) | 690 (3.78%) | 0.643994539883 | 0.706316592129 | EIF2AK1  CSNK1D  GNE  CSNK1A1  FGGY | web link |
| DNA binding | Gene Ontology | GO:0003677 | 15 (10.34%) | 2236 (12.24%) | 0.792541484524 | 0.842075327306 | NFIC  RCC1  NFIA  SERPINA3  STAT3  ZNF207  NFE2L2  HNRNPU  ATF6  ZFAND5  ZC3H12A  MLX  XBP1  NCOR2  AGFG1 | web link |
| DNA-binding transcription factor activity, RNA polymerase II-specific | Gene Ontology | GO:0000981 | 7 (4.83%) | 1231 (6.74%) | 0.864531171077 | 0.890729085352 | NFIC  NFIA  STAT3  NFE2L2  MLX  ATF6  XBP1 | web link |
| metal ion binding | Gene Ontology | GO:0046872 | 21 (14.48%) | 3785 (20.71%) | 0.978816184642 | 0.978816184642 | ZNF207  TGM2  RBCK1  CYP2D6  CYP4F12  FKBP8  PITRM1  KAT5  MASP2  ZC3H12A  HSPB11  ZCCHC9  SMPD1  ABLIM3  GNE  CYP2A7  MBNL3  ZFAND5  EWSR1  TUT7  AGFG1 | web link |
