# Supplementary material for: Comprehensive and Combined Omics Analysis Reveals Immune–Infiltration‐Associated Alternative Splicing and RBP Regulators Contributing to Ischemia–Reperfusion Injury in Liver Transplantation
Source: Int J Genomics. 2026 Sep 21;2026:4835678. doi: 10.1155/ijog/4835678 (PMC13592132; doi:10.1155/ijog/4835678)
Supplement: Supplementary file 2 [file IJOG-2026-4835678-s019.zip › Supplementary_Enrichment_Results/IRI_1_spec.txt.anno.gene_GO_analysis/IRI_1_spec.txt.anno.gene_GO_enrichment_web_P.html]

GO enrichment analyze


# GO enrichment analyze result


| Term | Database | ID | Input number (120) | Background number (17574) | P-Value | Corrected P-Value | Input | Hyperlink |
| --- | --- | --- | --- | --- | --- | --- | --- | --- |
| viral process | Gene Ontology | GO:0016032 | 18 (15.00%) | 596 (3.39%) | 1.18493685974e-07 | 5.0952284969e-06 | RBCK1  PVR  STAT3  NXF1  TMEM134  MRC1  NFE2L2  IFI27  KAT5  HNRNPU  CD4  KPNA1  ZC3H12A  CTBP1  KRT8  FKBP8  HTATIP2  RCC1 | web link |
| translation | Gene Ontology | GO:0006412 | 10 (8.33%) | 311 (1.77%) | 5.43141077405e-05 | 0.00116775331642 | MRPL11  GSPT1  TARS2  MRRF  MRPL42  RPS24  MRPL55  RPS2  MRPS11  GGT5 | web link |
| mitochondrial translational termination | Gene Ontology | GO:0070126 | 5 (4.17%) | 89 (0.51%) | 0.000358661853496 | 0.00514081990011 | MRPL42  MRPL11  MRRF  MRPS11  MRPL55 | web link |
| positive regulation of angiogenesis | Gene Ontology | GO:0045766 | 6 (5.00%) | 158 (0.90%) | 0.000754874682775 | 0.00811490283983 | STAT3  NFE2L2  HYAL1  CHI3L1  ZC3H12A  XBP1 | web link |
| oxidation-reduction process | Gene Ontology | GO:0055114 | 10 (8.33%) | 469 (2.67%) | 0.00141151789278 | 0.0100785743217 | ACOX1  CYP2D6  CYP4F12  DHODH  QSOX1  AASS  AKR1A1  CYP2A7  CTBP1  AKR1C2 | web link |
| platelet degranulation | Gene Ontology | GO:0002576 | 5 (4.17%) | 124 (0.71%) | 0.00161023747054 | 0.0100785743217 | CHID1  QSOX1  PROS1  A2M  SERPINA3 | web link |
| inflammatory response | Gene Ontology | GO:0006954 | 9 (7.50%) | 398 (2.26%) | 0.00164069814539 | 0.0100785743217 | SERPINA3  STAT3  NFE2L2  HYAL1  NFKBIZ  CHI3L1  GGT5  ZC3H12A  BLNK | web link |
| membrane organization | Gene Ontology | GO:0061024 | 5 (4.17%) | 131 (0.75%) | 0.00204961872319 | 0.0108660936806 | CD4  AGFG1  EPN1  PICALM  DNM2 | web link |
| fatty acid metabolic process | Gene Ontology | GO:0006631 | 6 (5.00%) | 196 (1.12%) | 0.00227429867733 | 0.0108660936806 | CYP2D6  CYP4F12  ACOX1  GGT5  FADS1  ECHDC2 | web link |
| positive regulation of protein phosphorylation | Gene Ontology | GO:0001934 | 6 (5.00%) | 206 (1.17%) | 0.00291288134929 | 0.0125253898019 | FAM20A  THPO  CD4  CSNK1D  RALB  XBP1 | web link |
| protein transport | Gene Ontology | GO:0015031 | 11 (9.17%) | 667 (3.80%) | 0.00595637052507 | 0.0211075581798 | WASHC3  NMD3  SCAMP3  AFM  HSPB11  NXF1  MIA3  ENY2  KPNA1  RAB17  XBP1 | web link |
| endocytosis | Gene Ontology | GO:0006897 | 6 (5.00%) | 240 (1.37%) | 0.00611064412084 | 0.0211075581798 | MRC1  EPN1  PICALM  MIA3  DNM2  RAB17 | web link |
| protein folding | Gene Ontology | GO:0006457 | 5 (4.17%) | 171 (0.97%) | 0.00638135479855 | 0.0211075581798 | HSPA9  QSOX1  FKBP8  ATF6  CDC37 | web link |
| negative regulation of transcription by RNA polymerase II | Gene Ontology | GO:0000122 | 13 (10.83%) | 880 (5.01%) | 0.00707967285592 | 0.021744709486 | NFIC  STAT3  AASS  IFI27  HNRNPU  KAT5  DDX5  PER1  ATN1  CTBP1  MLX  NCOR2  XBP1 | web link |
| metabolic process | Gene Ontology | GO:0008152 | 5 (4.17%) | 190 (1.08%) | 0.00982104621041 | 0.0281536658032 | NEU4  AASS  HYAL1  SMPD1  GNE | web link |
| positive regulation of apoptotic process | Gene Ontology | GO:0043065 | 7 (5.83%) | 379 (2.16%) | 0.0152902302792 | 0.0410924938754 | TGM2  DHODH  GADD45G  SMPD1  RBCK1  DNM2  ATF6 | web link |
| mRNA processing | Gene Ontology | GO:0006397 | 7 (5.83%) | 385 (2.19%) | 0.0165265525926 | 0.0418024565578 | SNRNP70  HNRNPU  SNRPA1  DDX5  MBNL3  RBM39  A1CF | web link |
| cell division | Gene Ontology | GO:0051301 | 7 (5.83%) | 412 (2.34%) | 0.0229762774936 | 0.0527023791716 | RCC1  CSNK1A1  ZNF207  HNRNPU  CCNY  CDC37  RALB | web link |
| lipid metabolic process | Gene Ontology | GO:0006629 | 9 (7.50%) | 607 (3.45%) | 0.0232870977735 | 0.0527023791716 | CYP2D6  CYP4F12  ACOX1  SULT1A2  NEU4  FADS1  ECHDC2  AKR1C2  XBP1 | web link |
| positive regulation of transcription by RNA polymerase II | Gene Ontology | GO:0045944 | 14 (11.67%) | 1149 (6.54%) | 0.0248835161156 | 0.0534995596485 | NFIC  NFIA  TFR2  STAT3  NFE2L2  KAT5  HNRNPU  PER1  ABLIM3  ZC3H12A  MLX  ATF6  HTATIP2  XBP1 | web link |
| carbohydrate metabolic process | Gene Ontology | GO:0005975 | 5 (4.17%) | 247 (1.41%) | 0.0272671111152 | 0.0558326560931 | NEU4  HYAL1  CHID1  CHI3L1  FGGY | web link |
| apoptotic process | Gene Ontology | GO:0006915 | 9 (7.50%) | 692 (3.94%) | 0.047455524016 | 0.0927539787586 | RALB  IFI27  GADD45G  CHI3L1  GRAMD4  ZC3H12A  FKBP8  HTATIP2  XBP1 | web link |
| RNA splicing | Gene Ontology | GO:0008380 | 5 (4.17%) | 301 (1.71%) | 0.0555510154347 | 0.103856246248 | DDX5  MBNL3  RBM39  HNRNPU  SNRPA1 | web link |
| response to drug | Gene Ontology | GO:0042493 | 5 (4.17%) | 306 (1.74%) | 0.0587939256202 | 0.105339116736 | STAT3  BCHE  SMPD1  DHODH  NFE2L2 | web link |
| proteolysis | Gene Ontology | GO:0006508 | 8 (6.67%) | 632 (3.60%) | 0.0678680322138 | 0.116733015408 | USP8  LONP2  TFR2  GGT5  PCSK6  TGM2  MASP2  PITRM1 | web link |
| spermatogenesis | Gene Ontology | GO:0007283 | 6 (5.00%) | 475 (2.70%) | 0.106884085549 | 0.176769833792 | ACOX1  SNRPA1  HSPB11  GGT5  DNM2  AGFG1 | web link |
| positive regulation of transcription, DNA-templated | Gene Ontology | GO:0045893 | 8 (6.67%) | 735 (4.18%) | 0.130121680075 | 0.207230823822 | STAT3  NFE2L2  KAT5  CD4  RBCK1  DNM2  ENY2  PICALM | web link |
| transmembrane transport | Gene Ontology | GO:0055085 | 7 (5.83%) | 641 (3.65%) | 0.149075831049 | 0.228937883397 | SLC39A14  SLC17A4  SLC22A7  SLC5A6  SLC25A18  ABCA8  ABCC3 | web link |
| cell cycle | Gene Ontology | GO:0007049 | 7 (5.83%) | 661 (3.76%) | 0.166296067472 | 0.24657692763 | USP8  RCC1  CSNK1A1  ZNF207  CCNY  HNRNPU  RALB | web link |
| positive regulation of cell population proliferation | Gene Ontology | GO:0008284 | 6 (5.00%) | 552 (3.14%) | 0.17637556457 | 0.246833883137 | STAT3  CHTOP  THPO  EMP2  AKR1C2  XBP1 | web link |
| ion transport | Gene Ontology | GO:0006811 | 7 (5.83%) | 674 (3.84%) | 0.177950008773 | 0.246833883137 | SLC39A14  SLC22A7  SLC17A4  ATP6V1G2-DDX39B  SLC5A6  SLC25A18  TMEM175 | web link |
| negative regulation of transcription, DNA-templated | Gene Ontology | GO:0045892 | 6 (5.00%) | 601 (3.42%) | 0.228241595681 | 0.306699644196 | NFIC  KAT5  PER1  CTBP1  MLX  NCOR2 | web link |
| cell differentiation | Gene Ontology | GO:0030154 | 9 (7.50%) | 1003 (5.71%) | 0.246469566967 | 0.320249854943 | PRRC2B  HNRNPU  GADD45G  HSPB11  CTBP1  ZC3H12A  XBP1  HTATIP2  AGFG1 | web link |
| regulation of transcription, DNA-templated | Gene Ontology | GO:0006355 | 12 (10.00%) | 1422 (8.09%) | 0.264039098252 | 0.320249854943 | NFIC  NFIA  STAT3  NFE2L2  KAT5  MED15  DNM2  FADS1  MLX  ATF6  EWSR1  XBP1 | web link |
| multicellular organism development | Gene Ontology | GO:0007275 | 10 (8.33%) | 1157 (6.58%) | 0.265874260619 | 0.320249854943 | XBP1  GADD45G  HNRNPU  THPO  CFDP1  NUMB  ZC3H12A  MBNL3  HTATIP2  AGFG1 | web link |
| positive regulation of gene expression | Gene Ontology | GO:0010628 | 5 (4.17%) | 509 (2.90%) | 0.268116157627 | 0.320249854943 | ZC3H12A  GCGR  STAT3  NFE2L2  BLNK | web link |
| negative regulation of apoptotic process | Gene Ontology | GO:0043066 | 5 (4.17%) | 543 (3.09%) | 0.313149224069 | 0.363930179323 | FKBP8  HSPA9  CTH  HTATIP2  XBP1 | web link |
| phosphorylation | Gene Ontology | GO:0016310 | 6 (5.00%) | 697 (3.97%) | 0.340833505515 | 0.385680019399 | STAT3  CSNK1A1  EIF2AK1  CSNK1D  FGGY  GNE | web link |
| protein phosphorylation | Gene Ontology | GO:0006468 | 5 (4.17%) | 610 (3.47%) | 0.404052319762 | 0.445493583327 | EIF2AK1  CTBP1  CSNK1D  CSNK1A1  FAM20A | web link |
| regulation of transcription by RNA polymerase II | Gene Ontology | GO:0006357 | 12 (10.00%) | 1641 (9.34%) | 0.446394837037 | 0.479874449815 | NFIC  NFIA  STAT3  NFE2L2  TSC22D3  KAT5  DDX5  MED15  ENY2  MLX  ATF6  HTATIP2 | web link |
| cell adhesion | Gene Ontology | GO:0007155 | 5 (4.17%) | 658 (3.74%) | 0.468651716764 | 0.491512776118 | CD4  PVR  EMP2  CFDP1  GNE | web link |
| immune system process | Gene Ontology | GO:0002376 | 6 (5.00%) | 818 (4.65%) | 0.488370935536 | 0.499998814953 | IFITM3  IFI27  MASP2  CD4  ZC3H12A  CHID1 | web link |
| signal transduction | Gene Ontology | GO:0007165 | 10 (8.33%) | 2064 (11.74%) | 0.909783872204 | 0.909783872204 | STAT3  CSNK1A1  THPO  CD4  ADGRA3  SMPD1  DNM2  GCGR  ATF6  RALB | web link |
