# Supplementary material for: Comprehensive and Combined Omics Analysis Reveals Immune–Infiltration‐Associated Alternative Splicing and RBP Regulators Contributing to Ischemia–Reperfusion Injury in Liver Transplantation
Source: Int J Genomics. 2026 Sep 21;2026:4835678. doi: 10.1155/ijog/4835678 (PMC13592132; doi:10.1155/ijog/4835678)
Supplement: Supplementary file 2 [file IJOG-2026-4835678-s019.zip › Supplementary_Enrichment_Results/IRI_1_spec.txt.anno.gene_GO_analysis/Rplots.pdf]

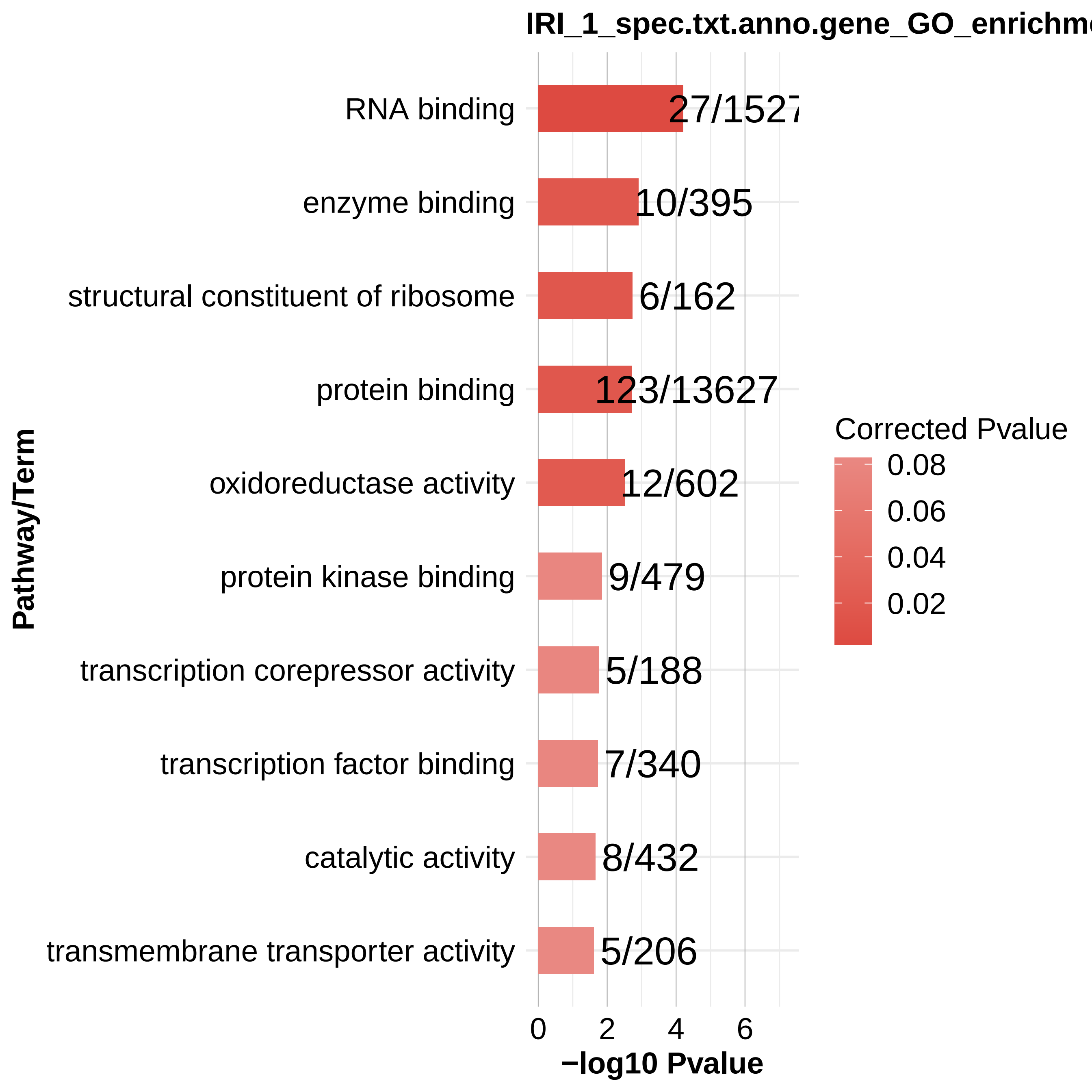

IRI_1_spec.txt.anno.gene_GO_enrichm
RNA binding
enzyme binding
structural constituent of ribosome
protein binding
oxidoreductase activity
protein kinase binding
transcription corepressor activity
transcription factor binding
catalytic activity
transmembrane transporter activity
27/1527
10/395
6/162
123/13627
12/602
9/479
5/188
7/340
8/432
5/206
Pathway/Term
0
2
4
6
−log10 Pvalue
Corrected Pvalue
0.08
0.06
0.04
0.02
