# Supplementary material for: Comprehensive and Combined Omics Analysis Reveals Immune–Infiltration‐Associated Alternative Splicing and RBP Regulators Contributing to Ischemia–Reperfusion Injury in Liver Transplantation
Source: Int J Genomics. 2026 Sep 21;2026:4835678. doi: 10.1155/ijog/4835678 (PMC13592132; doi:10.1155/ijog/4835678)
Supplement: Supplementary file 2 [file IJOG-2026-4835678-s019.zip › Supplementary_Enrichment_Results/IRI_2_spec.txt.anno.gene_GO_analysis/IRI_2_spec.txt.anno.gene_GO_enrichment_C_top.pdf]

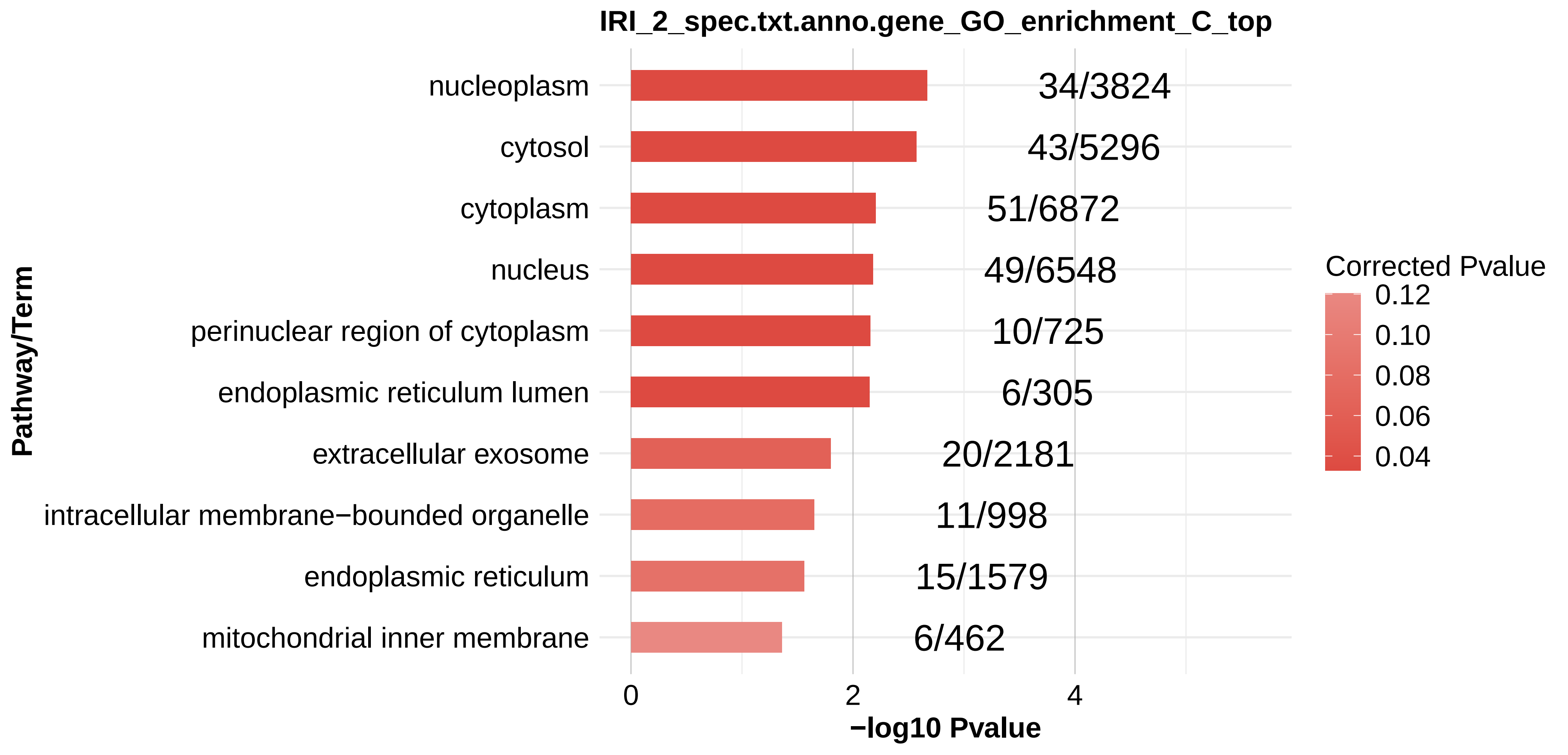

IRI_2_spec.txt.anno.gene_GO_enrichment_C_top
nucleoplasm
cytosol
cytoplasm
nucleus
perinuclear region of cytoplasm
endoplasmic reticulum lumen
extracellular exosome
intracellular membrane−bounded organelle
endoplasmic reticulum
mitochondrial inner membrane
34/3824
43/5296
51/6872
49/6548
10/725
6/305
20/2181
11/998
15/1579
6/462
Pathway/Term
0
2
4
−log10 Pvalue
Corrected Pvalue
0.12
0.10
0.08
0.06
0.04
