# Supplementary material for: Comprehensive and Combined Omics Analysis Reveals Immune–Infiltration‐Associated Alternative Splicing and RBP Regulators Contributing to Ischemia–Reperfusion Injury in Liver Transplantation
Source: Int J Genomics. 2026 Sep 21;2026:4835678. doi: 10.1155/ijog/4835678 (PMC13592132; doi:10.1155/ijog/4835678)
Supplement: Supplementary file 2 [file IJOG-2026-4835678-s019.zip › Supplementary_Enrichment_Results/IRI_2_spec.txt.anno.gene_GO_analysis/IRI_2_spec.txt.anno.gene_GO_enrichment_F_top.pdf]

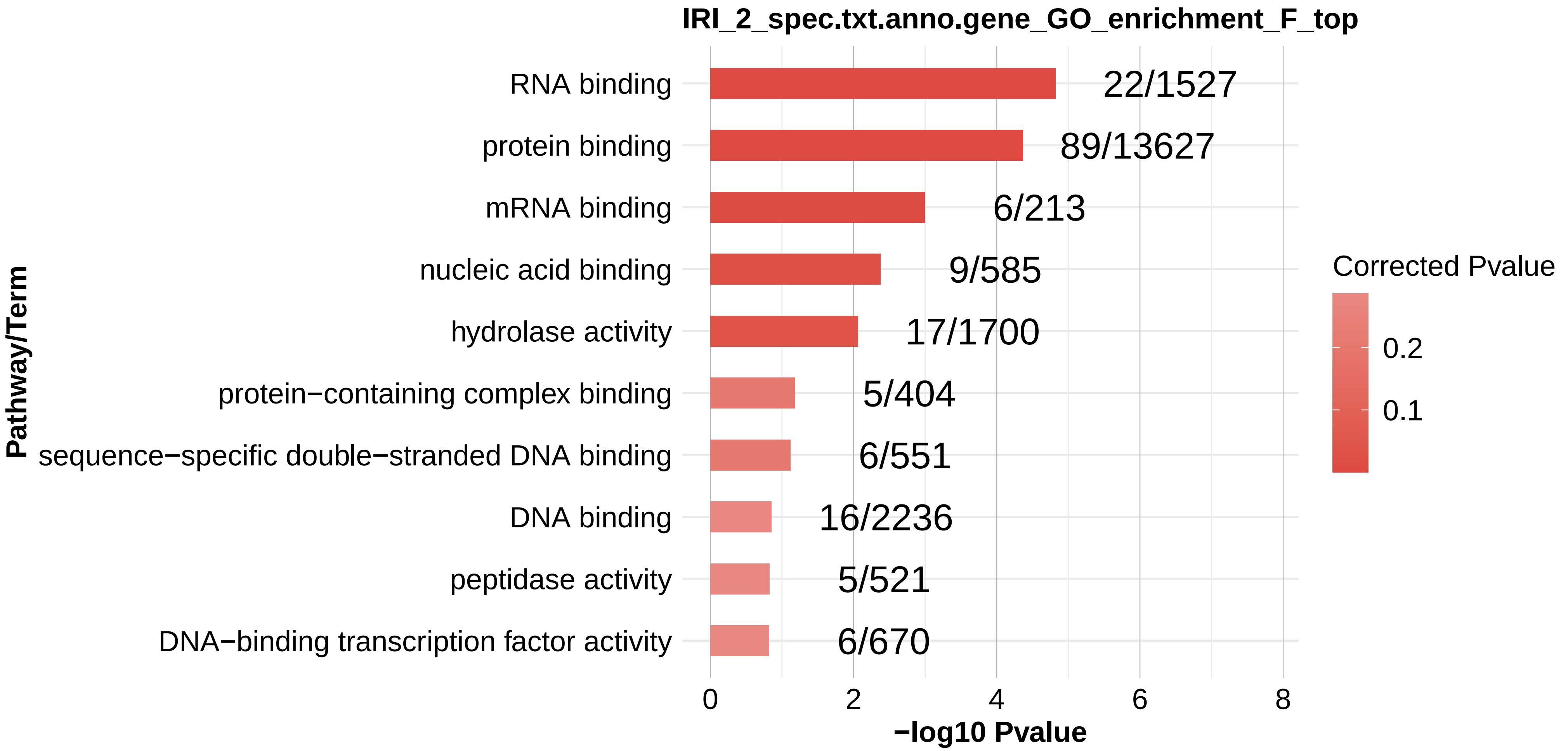

IRI_2_spec.txt.anno.gene_GO_enrichment_F_top
Pathway/Term
RNA binding
protein binding
mRNA binding
nucleic acid binding
hydrolase activity
protein−containing complex binding
sequence−specific double−stranded DNA binding
DNA binding
peptidase activity
DNA−binding transcription factor activity
22/1527
89/13627
6/213
9/585
17/1700
5/404
6/551
16/2236
5/521
6/670
0
2
4
6
8
−log10 Pvalue
Corrected Pvalue
0.2
0.1
