# Supplementary material for: Comprehensive and Combined Omics Analysis Reveals Immune–Infiltration‐Associated Alternative Splicing and RBP Regulators Contributing to Ischemia–Reperfusion Injury in Liver Transplantation
Source: Int J Genomics. 2026 Sep 21;2026:4835678. doi: 10.1155/ijog/4835678 (PMC13592132; doi:10.1155/ijog/4835678)
Supplement: Supplementary file 2 [file IJOG-2026-4835678-s019.zip › Supplementary_Enrichment_Results/IRI_2_spec.txt.anno.gene_GO_analysis/IRI_2_spec.txt.anno.gene_GO_enrichment_P_top.pdf]

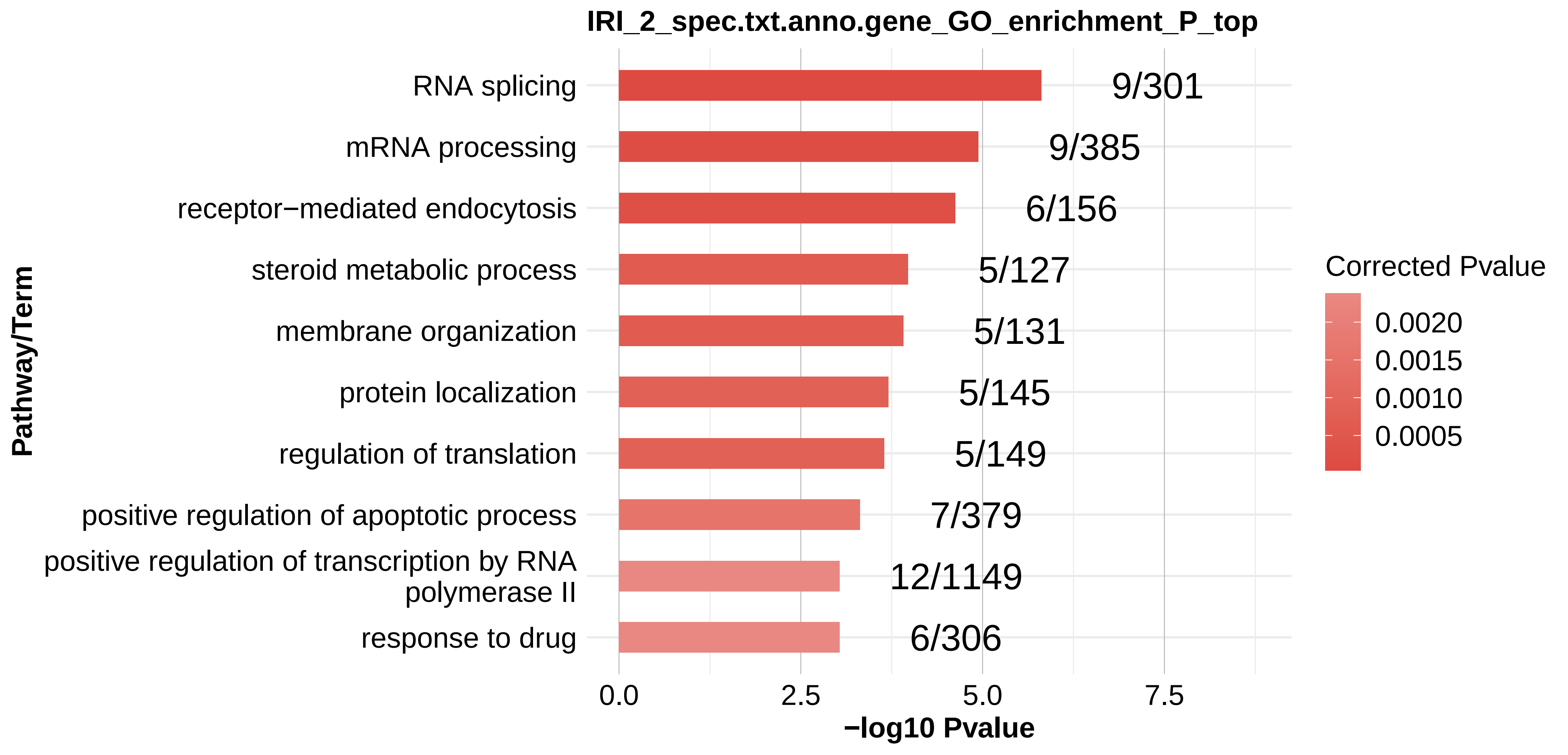
IRI_2_spec.txt.anno.gene_GO_enrichment_P_top
RNA splicing
mRNA processing
receptor−mediated endocytosis
steroid metabolic process
membrane organization
protein localization
regulation of translation
positive regulation of apoptotic process
positive regulation of transcription by RNA polymerase II
response to drug
9/301
9/385
6/156
5/127
5/131
5/145
5/149
7/379
12/1149
6/306
Pathway/Term
0.0
2.5
5.0
7.5
−log10 Pvalue
Corrected Pvalue
0.0020
0.0015
0.0010
0.0005
