# Supplementary material for: Comprehensive and Combined Omics Analysis Reveals Immune–Infiltration‐Associated Alternative Splicing and RBP Regulators Contributing to Ischemia–Reperfusion Injury in Liver Transplantation
Source: Int J Genomics. 2026 Sep 21;2026:4835678. doi: 10.1155/ijog/4835678 (PMC13592132; doi:10.1155/ijog/4835678)
Supplement: Supplementary file 2 [file IJOG-2026-4835678-s019.zip › Supplementary_Enrichment_Results/IRI_2_spec.txt.anno.gene_GO_analysis/IRI_2_spec.txt.anno.gene_GO_enrichment_web_C.html]

GO enrichment analyze


# GO enrichment analyze result


| Term | Database | ID | Input number (103) | Background number (18569) | P-Value | Corrected P-Value | Input | Hyperlink |
| --- | --- | --- | --- | --- | --- | --- | --- | --- |
| nucleoplasm | Gene Ontology | GO:0005654 | 34 (33.01%) | 3824 (20.59%) | 0.00214777227679 | 0.0329816310405 | MKNK2  UBE2D3  DEK  HNRNPH1  TXNL4B  ARL4A  GPS1  SRSF3  NFE2L1  BCLAF1  USF2  KDM2A  MBNL2  RBM39  TRA2B  NABP1  DDX3X  PARP9  CCDC59  HNRNPA1  SLC13A5  HNRNPAB  PER1  CNDP2  NFIC  PLSCR1  MAX  DHODH  RBPMS  SREBF1  RHBDD2  UBC  BCL6  PPP4C | web link |
| cytosol | Gene Ontology | GO:0005829 | 43 (41.75%) | 5296 (28.52%) | 0.00267984779883 | 0.0329816310405 | SYNPO  HSD17B14  RAB4A  UBE2D3  GPBP1  HNRNPH1  DNM2  SEC16A  SORBS3  TXNL4B  ARL4A  GPS1  APOB  EIF4G1  PPP4C  REPS1  ZSWIM8  NABP1  TGFBR2  DDX3X  PARP9  TRIM26  EPN1  SLC13A5  PER1  EIF4G2  GPT  LPIN2  ACMSD  CNDP2  CRAT  PLSCR1  PNP  DHODH  SDS  IL32  RBPMS  SREBF1  MIDN  UBC  FLNA  NFE2L1  EIF4A2 | web link |
| cytoplasm | Gene Ontology | GO:0005737 | 51 (49.51%) | 6872 (37.01%) | 0.00623000060727 | 0.0329816310405 | SYNPO  HSD17B14  RAB4A  MKNK2  GPBP1  SHROOM1  CHI3L1  DNM2  SEC16A  SORBS3  SORBS2  MAX  CDK2AP2  ARL4A  GPS1  LPIN2  EIF4G1  SRSF3  KANK1  PPP4C  UBC  BCLAF1  AKAP8L  HNRNPAB  MBNL2  ZSWIM8  TGFBR2  DDX3X  PARP9  ZBED6  TRIM26  HNRNPA1  EPN1  MTUS1  PER1  CLK1  GPT  APOB  ACMSD  CNDP2  MIDN  SDS  PNP  SCARB1  PLSCR1  RBPMS  SREBF1  EIF4G2  FLNA  SYBU  DHODH | web link |
| nucleus | Gene Ontology | GO:0005634 | 49 (47.57%) | 6548 (35.26%) | 0.00660940061555 | 0.0329816310405 | SYNPO  MKNK2  UBE2D3  GPBP1  DEK  HNRNPH1  DNM2  TXNL4B  SORBS2  SORBS3  CDK2AP2  ARL4A  GPS1  LPIN2  SRSF3  KANK1  NFE2L1  UBC  BCLAF1  USF2  AKAP8L  HNRNPAB  MBNL2  RBM39  TRA2B  NABP1  NFIC  DDX3X  PARP9  ZBED6  CCDC59  TRIM26  HNRNPA1  EPN1  MTUS1  PER1  FLNA  MIDN  SBNO2  PLSCR1  KDM2A  MAX  EIF4G1  RBPMS  SREBF1  PNP  CLK1  BCL6  PPP4C | web link |
| perinuclear region of cytoplasm | Gene Ontology | GO:0048471 | 10 (9.71%) | 725 (3.90%) | 0.00696808497172 | 0.0329816310405 | RAB4A  PLSCR1  RHBDD2  CHI3L1  DNM2  SEC16A  FLNA  SORBS2  TRA2B  EIF4A2 | web link |
| endoplasmic reticulum lumen | Gene Ontology | GO:0005788 | 6 (5.83%) | 305 (1.64%) | 0.00706749236583 | 0.0329816310405 | SIL1  APOB  F7  VWA1  COL27A1  CNPY3 | web link |
| extracellular exosome | Gene Ontology | GO:0070062 | 20 (19.42%) | 2181 (11.75%) | 0.0158943304597 | 0.063577321839 | HNRNPA1  APOB  CUTA  PLSCR1  PNP  SCARB1  DNM2  CFB  UBE2D3  SAA1  VWA1  DDX3X  GPT  SMPD1  CHI3L1  UBC  RAB4A  FLNA  CNDP2  FURIN | web link |
| intracellular membrane-bounded organelle | Gene Ontology | GO:0043231 | 11 (10.68%) | 998 (5.37%) | 0.0224388537974 | 0.0785359882908 | APOB  SPCS2  RAB4A  SCARB1  EPN1  USF2  GPBP1  SREBF1  SYBU  SEC16A  CYP3A5 | web link |
| endoplasmic reticulum | Gene Ontology | GO:0005783 | 15 (14.56%) | 1579 (8.50%) | 0.0274983468406 | 0.0855504123929 | CRAT  SIL1  VMP1  ZDHHC4  LPIN2  SPCS2  TLCD4  NFE2L1  SREBF1  CNPY3  CHI3L1  SEC16A  APOB  CYP3A5  FURIN | web link |
| mitochondrial inner membrane | Gene Ontology | GO:0005743 | 6 (5.83%) | 462 (2.49%) | 0.0436930872106 | 0.120244585283 | CRAT  DHODH  MRPL55  UQCRH  SFXN1  DNAJC19 | web link |
| microtubule | Gene Ontology | GO:0005874 | 5 (4.85%) | 354 (1.91%) | 0.0472389442184 | 0.120244585283 | MTUS1  SHROOM1  SYBU  CDK2AP2  DNM2 | web link |
| Golgi membrane | Gene Ontology | GO:0000139 | 7 (6.80%) | 620 (3.34%) | 0.0569754690984 | 0.13294276123 | ZDHHC4  SYBU  SREBF1  RHBDD2  DNM2  SEC16A  FURIN | web link |
| endoplasmic reticulum membrane | Gene Ontology | GO:0005789 | 10 (9.71%) | 1048 (5.64%) | 0.0653661990234 | 0.140788736358 | VMP1  APOB  ZDHHC4  SPCS2  NFE2L1  LPIN2  SREBF1  UBC  SEC16A  CYP3A5 | web link |
| nuclear speck | Gene Ontology | GO:0016607 | 5 (4.85%) | 410 (2.21%) | 0.0779982108 | 0.1559964216 | AKAP8L  RBM39  BCLAF1  SRSF3  FNBP4 | web link |
| nucleolus | Gene Ontology | GO:0005730 | 8 (7.77%) | 848 (4.57%) | 0.0987831910723 | 0.184395290002 | MIDN  VMP1  ZBED6  PLSCR1  MTUS1  FLNA  BCL6  ARL4A | web link |
| endosome | Gene Ontology | GO:0005768 | 7 (6.80%) | 728 (3.92%) | 0.109904424485 | 0.192332742848 | RAB4A  EPN1  UBE2D3  SMPD1  DNM2  GCGR  FURIN | web link |
| extracellular space | Gene Ontology | GO:0005615 | 14 (13.59%) | 1880 (10.12%) | 0.156625791733 | 0.257971892267 | SIL1  APOB  LIME1  F7  CFB  VWA1  MTUS1  COL27A1  IL32  CHI3L1  UBC  GPT  SMPD1  TNFAIP2 | web link |
| plasma membrane | Gene Ontology | GO:0005886 | 33 (32.04%) | 5167 (27.83%) | 0.197359390992 | 0.307003497098 | VMP1  RAB4A  FURIN  UBE2D3  GPBP1  SMPD1  SLC5A6  DNM2  SORBS2  IL1R1  ARL4A  PLXNB1  APOB  ADIPOR2  PPP4C  REPS1  PALM2AKAP2  TGFBR2  DDX3X  UBC  EPN1  SLC13A5  MTUS1  IL1RAP  GCGR  ZDHHC4  LIME1  F7  SCARB1  PLSCR1  CFB  KANK1  FLNA | web link |
| Golgi apparatus | Gene Ontology | GO:0005794 | 10 (9.71%) | 1366 (7.36%) | 0.225502958494 | 0.310263138104 | ZDHHC4  PLSCR1  DNM2  MTUS1  SREBF1  RHBDD2  SYBU  SEC16A  BCL6  FURIN | web link |
| protein-containing complex | Gene Ontology | GO:0032991 | 6 (5.83%) | 740 (3.99%) | 0.228047369921 | 0.310263138104 | PARP9  SREBF1  DNM2  RBM39  TRA2B  DNAJC19 | web link |
| membrane | Gene Ontology | GO:0016020 | 51 (49.51%) | 8478 (45.66%) | 0.245022103309 | 0.310263138104 | VMP1  CUTA  SPCS2  RAB4A  SFXN1  UBE2D3  PGAM5  HNRNPH1  SLC5A6  DNM2  SEC16A  SORBS2  IL1R1  ARL4A  PLXNB1  ADIPOR2  TLCD4  NFE2L1  REPS1  LPIN2  FNDC3B  FURIN  DNAJC19  TGFBR2  HNRNPA1  DDX3X  PARP9  UQCRH  SYBU  EPN1  SLC13A5  MTUS1  IL32  FLNA  GCGR  CYP3A5  ACMSD  ZDHHC4  CRAT  PALM2AKAP2  LIME1  IL1RAP  PLSCR1  EIF4G2  DHODH  SCARB1  SREBF1  RHBDD2  KANK1  SMPD1  EIF4G1 | web link |
| cytoskeleton | Gene Ontology | GO:0005856 | 10 (9.71%) | 1406 (7.57%) | 0.252444768759 | 0.310263138104 | SYNPO  PPP4C  KANK1  DNM2  DDX3X  MTUS1  SHROOM1  SYBU  FLNA  SORBS3 | web link |
| mitochondrion | Gene Ontology | GO:0005739 | 11 (10.68%) | 1573 (8.47%) | 0.254859006299 | 0.310263138104 | CRAT  PARP9  MRPL55  DHODH  SDS  MTUS1  PGAM5  UQCRH  SFXN1  CMC1  DNAJC19 | web link |
| extracellular region | Gene Ontology | GO:0005576 | 16 (15.53%) | 2461 (13.25%) | 0.286478308314 | 0.334224693033 | APOB  DDX3X  F7  PNP  IL32  SAA1  VWA1  COL27A1  PLXNB1  SMPD1  CHI3L1  IL1RAP  FLNA  CFB  IL1R1  FURIN | web link |
| chromatin | Gene Ontology | GO:0000785 | 7 (6.80%) | 980 (5.28%) | 0.301193431774 | 0.337336643587 | NFIC  ZBED6  MAX  NFE2L1  USF2  AKAP8L  SREBF1 | web link |
| cell projection | Gene Ontology | GO:0042995 | 7 (6.80%) | 1142 (6.15%) | 0.449059739229 | 0.483602796093 | SYNPO  MAX  KANK1  DDX3X  DNM2  FLNA  SORBS2 | web link |
| integral component of plasma membrane | Gene Ontology | GO:0005887 | 8 (7.77%) | 1398 (7.53%) | 0.516811108479 | 0.535952260644 | TGFBR2  PLXNB1  PLSCR1  SCARB1  SLC5A6  IL1RAP  GCGR  IL1R1 | web link |
| integral component of membrane | Gene Ontology | GO:0016021 | 30 (29.13%) | 5472 (29.47%) | 0.567627374352 | 0.567627374352 | VMP1  CUTA  SPCS2  PGAM5  SMPD1  SLC5A6  SFXN1  IL1R1  FURIN  PLXNB1  ADIPOR2  TLCD4  NFE2L1  FNDC3B  DNAJC19  TGFBR2  DDX3X  SLC13A5  IL1RAP  GCGR  CYP3A5  ACMSD  LIME1  ZDHHC4  PLSCR1  DHODH  SREBF1  RHBDD2  SYBU  SCARB1 | web link |
