# Supplementary material for: Comprehensive and Combined Omics Analysis Reveals Immune–Infiltration‐Associated Alternative Splicing and RBP Regulators Contributing to Ischemia–Reperfusion Injury in Liver Transplantation
Source: Int J Genomics. 2026 Sep 21;2026:4835678. doi: 10.1155/ijog/4835678 (PMC13592132; doi:10.1155/ijog/4835678)
Supplement: Supplementary file 2 [file IJOG-2026-4835678-s019.zip › Supplementary_Enrichment_Results/IRI_2_spec.txt.anno.gene_GO_analysis/IRI_2_spec.txt.anno.gene_GO_enrichment_web_F.html]

GO enrichment analyze


# GO enrichment analyze result


| Term | Database | ID | Input number (98) | Background number (18273) | P-Value | Corrected P-Value | Input | Hyperlink |
| --- | --- | --- | --- | --- | --- | --- | --- | --- |
| RNA binding | Gene Ontology | GO:0003723 | 22 (22.45%) | 1527 (8.36%) | 1.49817858226e-05 | 0.000284653930629 | GPBP1  DEK  HNRNPH1  SORBS2  SRSF3  BCLAF1  AKAP8L  HNRNPAB  FNDC3B  RBM39  TRA2B  NABP1  DDX3X  CCDC59  HNRNPA1  MBNL2  EIF4G2  EIF4G1  RBPMS  UBC  FLNA  EIF4A2 | web link |
| protein binding | Gene Ontology | GO:0005515 | 89 (90.82%) | 13627 (74.57%) | 4.28257905972e-05 | 0.000406845010674 | SYNPO  TLCD4  RAB4A  CLK1  CHI3L1  CDK2AP2  FURIN  GPS1  PPP4C  BCLAF1  CNPY3  KDM2A  MBNL2  NABP1  DDX3X  TRIM26  VWA1  MTUS1  PER1  CYP3A5  MAX  EIF4G1  RHBDD2  UBC  SMPD1  DHODH  UQCRH  HSD17B14  UBE2D3  DEK  SFXN1  SEC16A  IL1R1  ARL4A  PLXNB1  APOB  SRSF3  FNDC3B  PALM2AKAP2  RBM39  PARP9  CCDC59  EPN1  ZDHHC4  PLSCR1  RBPMS  TNFAIP2  FLNA  NFE2L1  EIF4A2  SCARB1  FNBP4  MKNK2  GPBP1  PGAM5  HNRNPH1  ADIPOR2  REPS1  USF2  AKAP8L  HNRNPAB  TGFBR2  HNRNPA1  SLC13A5  SIL1  LIME1  F7  SREBF1  VMP1  CUTA  EIF4G2  DNM2  SLC5A6  SORBS3  SORBS2  TXNL4B  TRA2B  DNAJC19  KANK1  IL32  IL1RAP  ACMSD  NFIC  SDS  PNP  CFB  MIDN  SYBU  BCL6 | web link |
| mRNA binding | Gene Ontology | GO:0003729 | 6 (6.12%) | 213 (1.17%) | 0.00100980199463 | 0.00639541263267 | DDX3X  EIF4G2  EIF4G1  RBPMS  HNRNPAB  TRA2B | web link |
| nucleic acid binding | Gene Ontology | GO:0003676 | 9 (9.18%) | 585 (3.20%) | 0.00417285071031 | 0.019821040874 | DDX3X  SRSF3  HNRNPA1  RBPMS  HNRNPH1  HNRNPAB  RBM39  TRA2B  EIF4A2 | web link |
| hydrolase activity | Gene Ontology | GO:0016787 | 17 (17.35%) | 1700 (9.30%) | 0.0086285357872 | 0.0327884359914 | F7  DDX3X  SPCS2  RAB4A  IL1RAP  PPP4C  PLSCR1  CFB  PGAM5  SMPD1  IL1R1  DNM2  LPIN2  FURIN  ACMSD  CNDP2  EIF4A2 | web link |
| protein-containing complex binding | Gene Ontology | GO:0044877 | 5 (5.10%) | 404 (2.21%) | 0.0662663462055 | 0.205880182875 | PGAM5  FLNA  SREBF1  DNM2  MAX | web link |
| sequence-specific double-stranded DNA binding | Gene Ontology | GO:1990837 | 6 (6.12%) | 551 (3.02%) | 0.0758505936907 | 0.205880182875 | NFIC  MAX  USF2  SREBF1  MBNL2  BCL6 | web link |
| DNA binding | Gene Ontology | GO:0003677 | 16 (16.33%) | 2236 (12.24%) | 0.139948380514 | 0.286470279148 | NFIC  BCL6  DDX3X  ZBED6  PLSCR1  MAX  TRIM26  BCLAF1  USF2  AKAP8L  DEK  KDM2A  SREBF1  GPBP1  NFE2L1  NABP1 | web link |
| peptidase activity | Gene Ontology | GO:0008233 | 5 (5.10%) | 521 (2.85%) | 0.148164590641 | 0.286470279148 | F7  CFB  SPCS2  CNDP2  FURIN | web link |
| DNA-binding transcription factor activity | Gene Ontology | GO:0003700 | 6 (6.12%) | 670 (3.67%) | 0.150773831131 | 0.286470279148 | NFIC  MAX  NFE2L1  USF2  SREBF1  BCL6 | web link |
| identical protein binding | Gene Ontology | GO:0042802 | 11 (11.22%) | 1628 (8.91%) | 0.255200337468 | 0.440800582899 | BCL6  HSD17B14  PNP  SCARB1  HNRNPA1  VWA1  HNRNPH1  MAX  SORBS2  EIF4G1  TRA2B | web link |
| zinc ion binding | Gene Ontology | GO:0008270 | 6 (6.12%) | 822 (4.50%) | 0.278864371556 | 0.441535254964 | PLSCR1  TRIM26  SMPD1  KDM2A  ZSWIM8  ACMSD | web link |
| kinase activity | Gene Ontology | GO:0016301 | 5 (5.10%) | 690 (3.78%) | 0.311557070089 | 0.455352640899 | MKNK2  CLK1  AKAP8L  CDK2AP2  TGFBR2 | web link |
| RNA polymerase II cis-regulatory region sequence-specific DNA binding | Gene Ontology | GO:0000978 | 7 (7.14%) | 1169 (6.40%) | 0.438375887161 | 0.594938704004 | NFIC  MAX  NFE2L1  USF2  PER1  SREBF1  BCL6 | web link |
| transferase activity | Gene Ontology | GO:0016740 | 10 (10.20%) | 1849 (10.12%) | 0.538061708218 | 0.681544830409 | CRAT  TGFBR2  PARP9  MKNK2  PNP  TRIM26  UBE2D3  GPT  CLK1  ZDHHC4 | web link |
| DNA-binding transcription factor activity, RNA polymerase II-specific | Gene Ontology | GO:0000981 | 6 (6.12%) | 1231 (6.74%) | 0.654168240105 | 0.686987481286 | NFIC  ZBED6  MAX  NFE2L1  USF2  SREBF1 | web link |
| nucleotide binding | Gene Ontology | GO:0000166 | 9 (9.18%) | 1828 (10.00%) | 0.656420157587 | 0.686987481286 | TGFBR2  DDX3X  RAB4A  MKNK2  UBE2D3  DNM2  CLK1  ARL4A  EIF4A2 | web link |
| metal ion binding | Gene Ontology | GO:0046872 | 19 (19.39%) | 3785 (20.71%) | 0.666337683187 | 0.686987481286 | TGFBR2  CYP3A5  ADIPOR2  ZBED6  MKNK2  TRIM26  REPS1  AKAP8L  COL27A1  PPP4C  SMPD1  KDM2A  CMC1  MBNL2  BCL6  ZSWIM8  ACMSD  CNDP2  FURIN | web link |
| ATP binding | Gene Ontology | GO:0005524 | 7 (7.14%) | 1476 (8.08%) | 0.686987481286 | 0.686987481286 | TGFBR2  DDX3X  MKNK2  EIF4G1  UBE2D3  CLK1  EIF4A2 | web link |
