# Supplementary material for: Comprehensive and Combined Omics Analysis Reveals Immune–Infiltration‐Associated Alternative Splicing and RBP Regulators Contributing to Ischemia–Reperfusion Injury in Liver Transplantation
Source: Int J Genomics. 2026 Sep 21;2026:4835678. doi: 10.1155/ijog/4835678 (PMC13592132; doi:10.1155/ijog/4835678)
Supplement: Supplementary file 2 [file IJOG-2026-4835678-s019.zip › Supplementary_Enrichment_Results/IRI_2_spec.txt.anno.gene_GO_analysis/IRI_2_spec.txt.anno.gene_GO_enrichment_web_P.html]

GO enrichment analyze


# GO enrichment analyze result


| Term | Database | ID | Input number (65) | Background number (17574) | P-Value | Corrected P-Value | Input | Hyperlink |
| --- | --- | --- | --- | --- | --- | --- | --- | --- |
| RNA splicing | Gene Ontology | GO:0008380 | 9 (13.85%) | 301 (1.71%) | 1.55201934859e-06 | 4.03525030634e-05 | SRSF3  HNRNPA1  BCLAF1  AKAP8L  HNRNPH1  TXNL4B  RBM39  MBNL2  TRA2B | web link |
| mRNA processing | Gene Ontology | GO:0006397 | 9 (13.85%) | 385 (2.19%) | 1.14538381513e-05 | 0.000148899895966 | SRSF3  HNRNPA1  BCLAF1  AKAP8L  HNRNPH1  MBNL2  RBM39  TXNL4B  TRA2B | web link |
| receptor-mediated endocytosis | Gene Ontology | GO:0006898 | 6 (9.23%) | 156 (0.89%) | 2.38226932758e-05 | 0.000206463341724 | TGFBR2  APOB  SCARB1  REPS1  SAA1  DNM2 | web link |
| steroid metabolic process | Gene Ontology | GO:0008202 | 5 (7.69%) | 127 (0.72%) | 0.000106316218151 | 0.000639854765347 | NFE2L1  CYP3A5  APOB  HSD17B14  SREBF1 | web link |
| membrane organization | Gene Ontology | GO:0061024 | 5 (7.69%) | 131 (0.75%) | 0.000123048993336 | 0.000639854765347 | APOB  EPN1  DNM2  REPS1  UBC | web link |
| protein localization | Gene Ontology | GO:0008104 | 5 (7.69%) | 145 (0.83%) | 0.000197966153098 | 0.00083455259535 | CRAT  BCL6  CUTA  UBE2D3  UBC | web link |
| regulation of translation | Gene Ontology | GO:0006417 | 5 (7.69%) | 149 (0.85%) | 0.00022468723721 | 0.00083455259535 | MKNK2  EIF4G2  EIF4G1  DDX3X  BCLAF1 | web link |
| positive regulation of apoptotic process | Gene Ontology | GO:0043065 | 7 (10.77%) | 379 (2.16%) | 0.000487496187786 | 0.00158436261031 | DDX3X  DHODH  DNM2  BCLAF1  SMPD1  UBC  BCL6 | web link |
| positive regulation of transcription by RNA polymerase II | Gene Ontology | GO:0045944 | 12 (18.46%) | 1149 (6.54%) | 0.000918923431599 | 0.0023780129019 | NFIC  SBNO2  LPIN2  PLSCR1  MAX  BCLAF1  USF2  AKAP8L  PER1  SREBF1  UBC  DDX3X | web link |
| response to drug | Gene Ontology | GO:0042493 | 6 (9.23%) | 306 (1.74%) | 0.000925295121064 | 0.0023780129019 | TGFBR2  ADIPOR2  PNP  DHODH  SREBF1  SMPD1 | web link |
| cytokine-mediated signaling pathway | Gene Ontology | GO:0019221 | 6 (9.23%) | 311 (1.77%) | 0.00100608238157 | 0.0023780129019 | UBC  SAA1  IL32  IL1RAP  BCL6  IL1R1 | web link |
| negative regulation of transcription by RNA polymerase II | Gene Ontology | GO:0000122 | 10 (15.38%) | 880 (5.01%) | 0.00137790251433 | 0.00298545544772 | NFIC  PARP9  ZBED6  MAX  UBE2D3  PER1  SREBF1  UBC  SORBS3  BCL6 | web link |
| viral process | Gene Ontology | GO:0016032 | 8 (12.31%) | 596 (3.39%) | 0.00153609112505 | 0.0030721822501 | DDX3X  PLSCR1  EIF4G1  HNRNPA1  AKAP8L  EIF4A2  SCARB1  FURIN | web link |
| lipid metabolic process | Gene Ontology | GO:0006629 | 8 (12.31%) | 607 (3.45%) | 0.00172372857596 | 0.0032012102125 | CRAT  APOB  HSD17B14  ADIPOR2  NFE2L1  SREBF1  LPIN2  CYP3A5 | web link |
| mRNA splicing, via spliceosome | Gene Ontology | GO:0000398 | 5 (7.69%) | 255 (1.45%) | 0.00251465347127 | 0.00435873268353 | TXNL4B  HNRNPA1  HNRNPH1  SRSF3  TRA2B | web link |
| innate immune response | Gene Ontology | GO:0045087 | 7 (10.77%) | 598 (3.40%) | 0.00643658066079 | 0.0101091201404 | DDX3X  PARP9  TRIM26  CFB  SAA1  CNPY3  IL1RAP | web link |
| negative regulation of transcription, DNA-templated | Gene Ontology | GO:0045892 | 7 (10.77%) | 601 (3.42%) | 0.00660980932254 | 0.0101091201404 | NFIC  SBNO2  ZBED6  EIF4G2  BCLAF1  PER1  BCL6 | web link |
| immune system process | Gene Ontology | GO:0002376 | 8 (12.31%) | 818 (4.65%) | 0.0102907265609 | 0.0148643828101 | DDX3X  PARP9  TRIM26  CFB  CNPY3  IL1RAP  BCL6  LIME1 | web link |
| apoptotic process | Gene Ontology | GO:0006915 | 7 (10.77%) | 692 (3.94%) | 0.0137086675947 | 0.0187592293401 | TGFBR2  DDX3X  PLSCR1  MKNK2  BCLAF1  UBE2D3  CHI3L1 | web link |
| positive regulation of transcription, DNA-templated | Gene Ontology | GO:0045893 | 7 (10.77%) | 735 (4.18%) | 0.0185069734467 | 0.0240590654807 | PARP9  RBPMS  USF2  GPBP1  SREBF1  DNM2  HNRNPAB | web link |
| positive regulation of gene expression | Gene Ontology | GO:0010628 | 5 (7.69%) | 509 (2.90%) | 0.0399061731887 | 0.0494076429955 | IL32  GCGR  APOB  DDX3X  PLSCR1 | web link |
| regulation of transcription by RNA polymerase II | Gene Ontology | GO:0006357 | 10 (15.38%) | 1641 (9.34%) | 0.0781745154787 | 0.0923880637475 | NFIC  LIME1  ZBED6  MAX  NFE2L1  SREBF1  USF2  DEK  KDM2A  BCL6 | web link |
| proteolysis | Gene Ontology | GO:0006508 | 5 (7.69%) | 632 (3.60%) | 0.0841474172583 | 0.0951231673355 | FURIN  CFB  SPCS2  CNDP2  F7 | web link |
| phosphorylation | Gene Ontology | GO:0016310 | 5 (7.69%) | 697 (3.97%) | 0.115035623751 | 0.12462192573 | MKNK2  AKAP8L  TGFBR2  CDK2AP2  CLK1 | web link |
| regulation of transcription, DNA-templated | Gene Ontology | GO:0006355 | 7 (10.77%) | 1422 (8.09%) | 0.271441790325 | 0.282299461938 | NFIC  SBNO2  MAX  NFE2L1  GPBP1  SREBF1  DNM2 | web link |
| signal transduction | Gene Ontology | GO:0007165 | 7 (10.77%) | 2064 (11.74%) | 0.653994224731 | 0.653994224731 | PLXNB1  IL1RAP  DEK  SMPD1  DNM2  GCGR  IL1R1 | web link |
