# Supplementary material for: Comprehensive and Combined Omics Analysis Reveals Immune–Infiltration‐Associated Alternative Splicing and RBP Regulators Contributing to Ischemia–Reperfusion Injury in Liver Transplantation
Source: Int J Genomics. 2026 Sep 21;2026:4835678. doi: 10.1155/ijog/4835678 (PMC13592132; doi:10.1155/ijog/4835678)
Supplement: Supplementary file 2 [file IJOG-2026-4835678-s019.zip › Supplementary_Enrichment_Results/IRI_2_spec.txt.anno.gene_GO_analysis/Rplots.pdf]

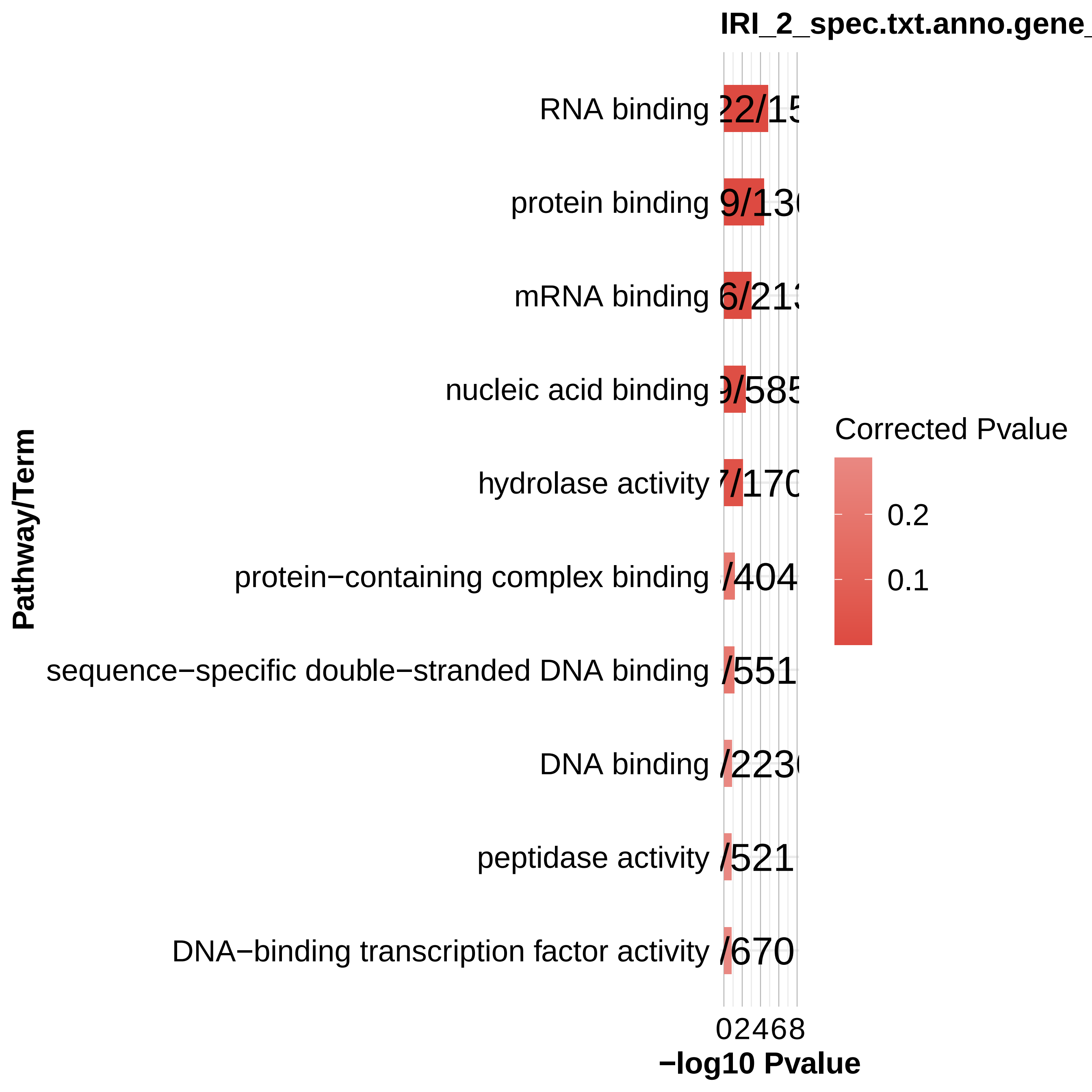

IRI_2_spec.txt.anno.gene_
RNA binding
22/15
protein binding
9/130
mRNA binding
6/213
nucleic acid binding
9/585
hydrolase activity
7/170
protein−containing complex binding
/404
sequence−specific double−stranded DNA binding
/551
DNA binding
/2230
peptidase activity
/521
DNA−binding transcription factor activity
/670
Corrected Pvalue
0.2
0.1
Pathway/Term
0
2
4
6
8
−log10 Pvalue
