# Supplementary material for: Comprehensive and Combined Omics Analysis Reveals Immune–Infiltration‐Associated Alternative Splicing and RBP Regulators Contributing to Ischemia–Reperfusion Injury in Liver Transplantation
Source: Int J Genomics. 2026 Sep 21;2026:4835678. doi: 10.1155/ijog/4835678 (PMC13592132; doi:10.1155/ijog/4835678)
Supplement: Supplementary file 2 [file IJOG-2026-4835678-s019.zip › Supplementary_Enrichment_Results/olp.txt.anno.gene_GO_analysis/olp.txt.anno.gene_GO_enrichment_C_top.pdf]

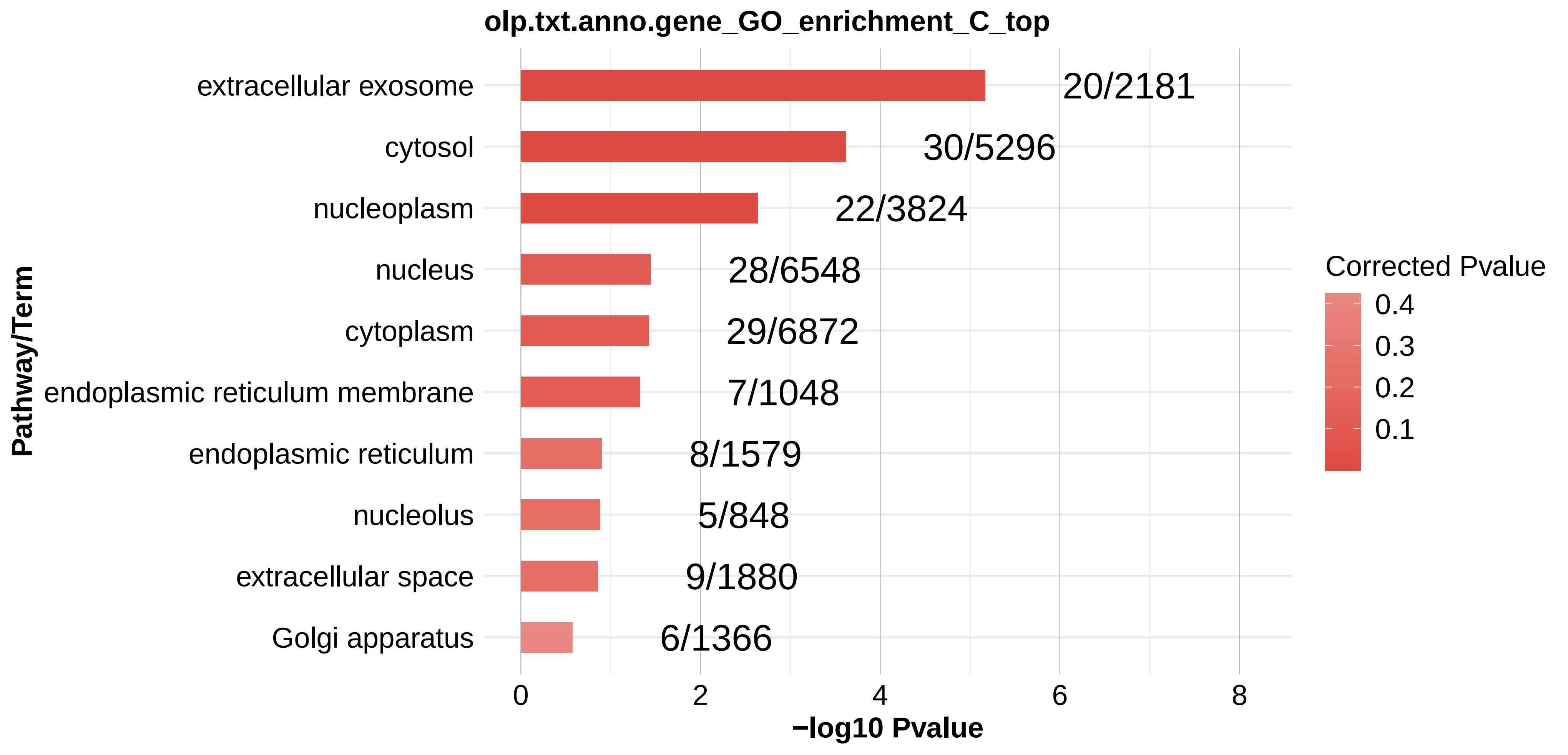
olp.txt.anno.gene_GO_enrichment_C_top
Pathway/Term
extracellular exosome
cytosol
nucleoplasm
nucleus
cytoplasm
endoplasmic reticulum membrane
endoplasmic reticulum
nucleolus
extracellular space
Golgi apparatus
20/2181
30/5296
22/3824
28/6548
29/6872
7/1048
8/1579
5/848
9/1880
6/1366
0
2
4
6
8
−log10 Pvalue
Corrected Pvalue
0.4
0.3
0.2
0.1
