# Supplementary material for: Comprehensive and Combined Omics Analysis Reveals Immune–Infiltration‐Associated Alternative Splicing and RBP Regulators Contributing to Ischemia–Reperfusion Injury in Liver Transplantation
Source: Int J Genomics. 2026 Sep 21;2026:4835678. doi: 10.1155/ijog/4835678 (PMC13592132; doi:10.1155/ijog/4835678)
Supplement: Supplementary file 2 [file IJOG-2026-4835678-s019.zip › Supplementary_Enrichment_Results/olp.txt.anno.gene_GO_analysis/olp.txt.anno.gene_GO_enrichment_P_top.pdf]

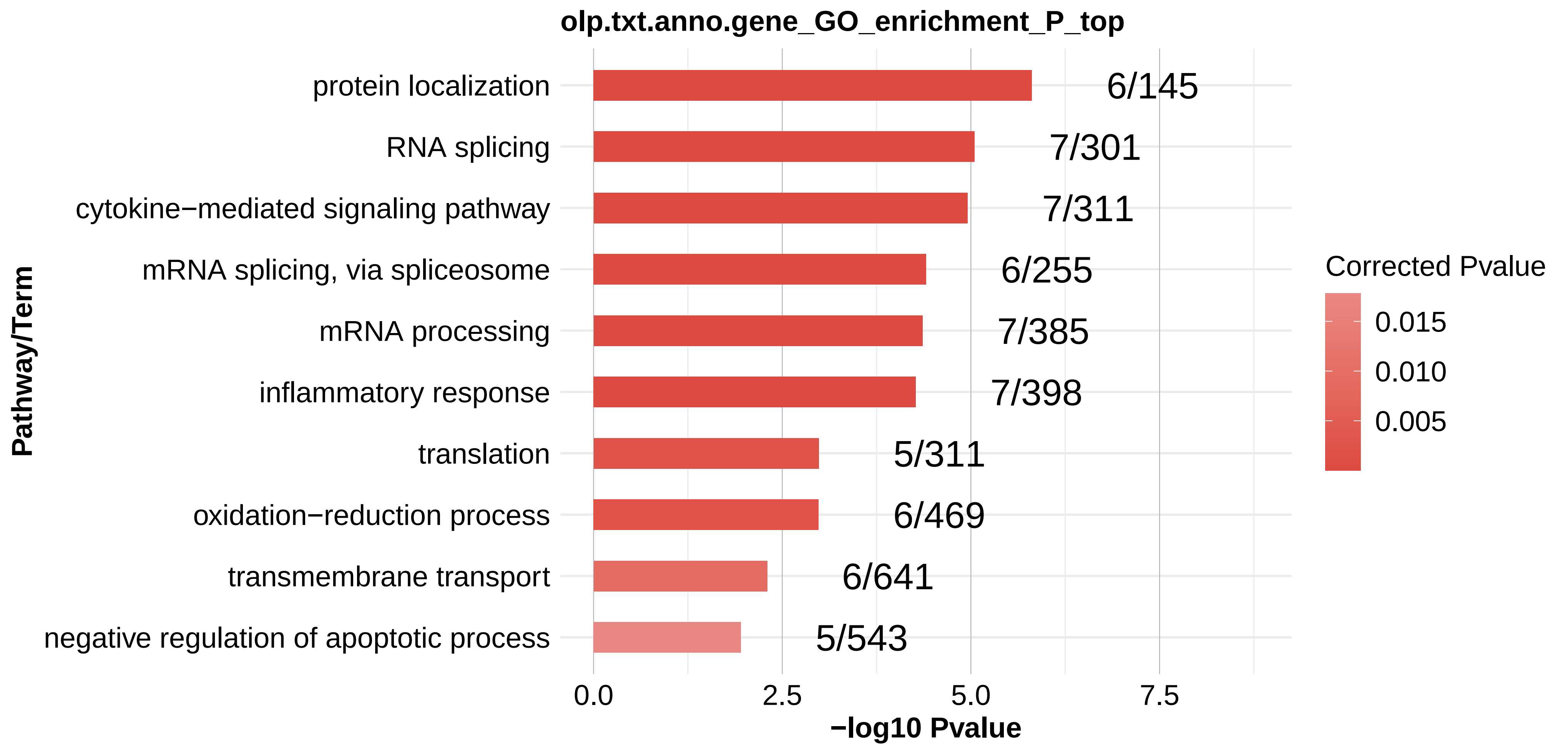
olp.txt.anno.gene_GO_enrichment_P_top
protein localization
RNA splicing
cytokine−mediated signaling pathway
mRNA splicing, via spliceosome
mRNA processing
inflammatory response
translation
oxidation−reduction process
transmembrane transport
negative regulation of apoptotic process
6/145
7/301
7/311
6/255
7/385
7/398
5/311
6/469
6/641
5/543
Pathway/Term
0.0
2.5
5.0
7.5
−log10 Pvalue
Corrected Pvalue
0.015
0.010
0.005
