# Supplementary material for: Comprehensive and Combined Omics Analysis Reveals Immune–Infiltration‐Associated Alternative Splicing and RBP Regulators Contributing to Ischemia–Reperfusion Injury in Liver Transplantation
Source: Int J Genomics. 2026 Sep 21;2026:4835678. doi: 10.1155/ijog/4835678 (PMC13592132; doi:10.1155/ijog/4835678)
Supplement: Supplementary file 2 [file IJOG-2026-4835678-s019.zip › Supplementary_Enrichment_Results/olp.txt.anno.gene_GO_analysis/olp.txt.anno.gene_GO_enrichment_web_C.html]

GO enrichment analyze


# GO enrichment analyze result


| Term | Database | ID | Input number (59) | Background number (18569) | P-Value | Corrected P-Value | Input | Hyperlink |
| --- | --- | --- | --- | --- | --- | --- | --- | --- |
| extracellular exosome | Gene Ontology | GO:0070062 | 20 (33.90%) | 2181 (11.75%) | 6.74858883942e-06 | 0.000107977421431 | ST3GAL1  EIF3I  SRSF7  KRT8  CHI3L1  IL1RN  H2AC6  HP  PLD3  UBE2D3  SAA1  AP2M1  DDX5  FASN  UBB  UBC  BAIAP2  F11  AKR1C1  UGP2 | web link |
| cytosol | Gene Ontology | GO:0005829 | 30 (50.85%) | 5296 (28.52%) | 0.000241145204642 | 0.00192916163714 | NFKBIA  CDO1  ELOC  TXNL4B  PLIN5  AKR1C1  UGP2  RARA  EIF3I  KRT8  NOP58  ANAPC16  NUDC  ARHGEF10L  AP2M1  FASN  HNRNPF  HDLBP  UBE2D3  IL32  ACMSD  EIF4G1  BAAT  SIGMAR1  DDX5  UBB  UBC  BAIAP2  HAO2  EEF1D | web link |
| nucleoplasm | Gene Ontology | GO:0005654 | 22 (37.29%) | 3824 (20.59%) | 0.00231449919205 | 0.0123439956909 | NFKBIA  UBE2D3  ELOC  TXNL4B  KRT8  RARA  SRSF7  NOP58  SRSF3  ANAPC16  NUDC  APEX1  MBNL2  HNRNPF  H2AC6  EEF1D  SRRM2  DDX5  UBB  UBC  BAIAP2  BCL6 | web link |
| nucleus | Gene Ontology | GO:0005634 | 28 (47.46%) | 6548 (35.26%) | 0.0356663753248 | 0.119946215841 | PNRC1  RCAN1  NFKBIA  UBE2D3  ELOC  TXNL4B  KRT8  UGP2  RARA  SRSF7  NCOA7  SRSF3  ANAPC16  NUDC  APEX1  MBNL2  HNRNPF  H2AC6  HDLBP  EEF1D  NOP58  EIF4G1  SRRM2  DDX5  UBB  UBC  BCL6  SIGMAR1 | web link |
| cytoplasm | Gene Ontology | GO:0005737 | 29 (49.15%) | 6872 (37.01%) | 0.0374831924503 | 0.119946215841 | RCAN1  NFKBIA  CHI3L1  KRT8  ARHGEF10L  UGP2  RARA  EIF3I  SRSF7  PLIN5  SRSF3  ANAPC16  NUDC  AKR1C1  APEX1  FASN  MBNL2  SLC38A2  HDLBP  CYP2A7  ACMSD  IL1RN  EEF1D  BAAT  DDX5  UBB  UBC  BAIAP2  EIF4G1 | web link |
| endoplasmic reticulum membrane | Gene Ontology | GO:0005789 | 7 (11.86%) | 1048 (5.64%) | 0.0474682659055 | 0.126582042415 | FMO3  PLD3  UBC  CYP2A7  UBB  GRAMD4  SIGMAR1 | web link |
| endoplasmic reticulum | Gene Ontology | GO:0005783 | 8 (13.56%) | 1579 (8.50%) | 0.125384837335 | 0.24598354029 | FMO3  PLD3  EEF1D  APEX1  CYP2A7  CHI3L1  GRAMD4  SIGMAR1 | web link |
| nucleolus | Gene Ontology | GO:0005730 | 5 (8.47%) | 848 (4.57%) | 0.131342748421 | 0.24598354029 | RARA  DDX5  BCL6  NOP58  APEX1 | web link |
| extracellular space | Gene Ontology | GO:0005615 | 9 (15.25%) | 1880 (10.12%) | 0.138365741413 | 0.24598354029 | IL1RN  CXCL2  CHI3L1  MRPL18  HP  IL32  UBB  UBC  F11 | web link |
| Golgi apparatus | Gene Ontology | GO:0005794 | 6 (10.17%) | 1366 (7.36%) | 0.265459667949 | 0.424735468718 | LTBR  ST3GAL1  PLD3  FASN  BAIAP2  BCL6 | web link |
| mitochondrion | Gene Ontology | GO:0005739 | 6 (10.17%) | 1573 (8.47%) | 0.383523921293 | 0.557852976427 | MRPL34  MRPL18  APEX1  UBB  GRAMD4  PLIN5 | web link |
| integral component of plasma membrane | Gene Ontology | GO:0005887 | 5 (8.47%) | 1398 (7.53%) | 0.460229648636 | 0.593014987533 | ABCC2  SLC22A7  SLC5A6  SLC17A2  SLC38A2 | web link |
| plasma membrane | Gene Ontology | GO:0005886 | 17 (28.81%) | 5167 (27.83%) | 0.481824677371 | 0.593014987533 | LTBR  IL1RN  NFKBIA  UBB  SLC38A2  HDLBP  UBE2D3  AP2M1  SLC22A7  FASN  HNRNPF  SLC5A6  UBC  BAIAP2  F11  SIGMAR1  ABCC2 | web link |
| extracellular region | Gene Ontology | GO:0005576 | 8 (13.56%) | 2461 (13.25%) | 0.530082960738 | 0.605809097986 | IL1RN  ST3GAL1  CXCL2  HP  SAA1  IL32  CHI3L1  F11 | web link |
| membrane | Gene Ontology | GO:0016020 | 24 (40.68%) | 8478 (45.66%) | 0.815639874578 | 0.868087974332 | UBE2D3  SLC5A6  LTBR  NOP58  NUDC  AP2M1  FASN  GRAMD4  HNRNPF  FMO3  ST3GAL1  SLC38A2  IL32  BAIAP2  F11  ACMSD  PLD3  EIF4G1  SLC17A2  DDX5  SLC22A7  CYP2A7  SIGMAR1  ABCC2 | web link |
| integral component of membrane | Gene Ontology | GO:0016021 | 14 (23.73%) | 5472 (29.47%) | 0.868087974332 | 0.868087974332 | LTBR  FMO3  ST3GAL1  SLC38A2  PLD3  NUDC  SLC17A2  SIGMAR1  SLC22A7  CYP2A7  SLC5A6  GRAMD4  ACMSD  ABCC2 | web link |
