# Supplementary material for: Comprehensive and Combined Omics Analysis Reveals Immune–Infiltration‐Associated Alternative Splicing and RBP Regulators Contributing to Ischemia–Reperfusion Injury in Liver Transplantation
Source: Int J Genomics. 2026 Sep 21;2026:4835678. doi: 10.1155/ijog/4835678 (PMC13592132; doi:10.1155/ijog/4835678)
Supplement: Supplementary file 2 [file IJOG-2026-4835678-s019.zip › Supplementary_Enrichment_Results/olp.txt.anno.gene_GO_analysis/olp.txt.anno.gene_GO_enrichment_web_F.html]

GO enrichment analyze


# GO enrichment analyze result


| Term | Database | ID | Input number (57) | Background number (18273) | P-Value | Corrected P-Value | Input | Hyperlink |
| --- | --- | --- | --- | --- | --- | --- | --- | --- |
| RNA binding | Gene Ontology | GO:0003723 | 15 (26.32%) | 1527 (8.36%) | 4.81484882897e-05 | 0.000329567170706 | EIF3I  SRSF7  SRRM2  NOP58  SRSF3  MRPL18  EIF4G1  HDLBP  APEX1  DDX5  FASN  UBC  MBNL2  CCDC9  HNRNPF | web link |
| protein binding | Gene Ontology | GO:0005515 | 54 (94.74%) | 13627 (74.57%) | 7.32371490457e-05 | 0.000329567170706 | PNRC1  RCAN1  HP  NFKBIA  UBE2D3  APEX1  ELOC  SLC5A6  TXNL4B  KRT8  AKR1C1  UGP2  RARA  EIF3I  SRSF7  F11  NCOA7  SRSF3  LTBR  CHI3L1  ANAPC16  NUDC  ARHGEF10L  AP2M1  FASN  GRAMD4  MBNL2  HNRNPF  FMO3  SLC38A2  MRPL18  H2AC6  HDLBP  EEF1D  IL32  CCDC9  ACMSD  NOP58  PLIN5  IL1RN  CXCL2  PLD3  EIF4G1  SRRM2  BAAT  DDX5  SLC22A7  UBB  UBC  BAIAP2  HAO2  BCL6  SIGMAR1  ABCC2 | web link |
| oxidoreductase activity | Gene Ontology | GO:0016491 | 7 (12.28%) | 602 (3.29%) | 0.00257803309722 | 0.00773409929165 | FMO3  CDO1  APEX1  FASN  CYP2A7  HAO2  AKR1C1 | web link |
| nucleic acid binding | Gene Ontology | GO:0003676 | 6 (10.53%) | 585 (3.20%) | 0.00955097130982 | 0.0196110142827 | SRSF7  SRSF3  RCAN1  HDLBP  DDX5  HNRNPF | web link |
| identical protein binding | Gene Ontology | GO:0042802 | 11 (19.30%) | 1628 (8.91%) | 0.0108950079348 | 0.0196110142827 | LTBR  F11  RCAN1  EIF4G1  NFKBIA  FASN  BAIAP2  PLIN5  SIGMAR1  UGP2  BCL6 | web link |
| hydrolase activity | Gene Ontology | GO:0016787 | 7 (12.28%) | 1700 (9.30%) | 0.277621723395 | 0.416432585093 | PLD3  BAAT  APEX1  DDX5  FASN  F11  ACMSD | web link |
| transferase activity | Gene Ontology | GO:0016740 | 5 (8.77%) | 1849 (10.12%) | 0.696663506633 | 0.862208611201 | BAAT  ST3GAL1  FASN  UBE2D3  UGP2 | web link |
| DNA binding | Gene Ontology | GO:0003677 | 5 (8.77%) | 2236 (12.24%) | 0.842688046853 | 0.862208611201 | RARA  H2AC6  EEF1D  BCL6  APEX1 | web link |
| metal ion binding | Gene Ontology | GO:0046872 | 9 (15.79%) | 3785 (20.71%) | 0.862208611201 | 0.862208611201 | RARA  SRSF7  CDO1  APEX1  CYP2A7  MBNL2  BCL6  ACMSD  UGP2 | web link |
