# Supplementary material for: Comprehensive and Combined Omics Analysis Reveals Immune–Infiltration‐Associated Alternative Splicing and RBP Regulators Contributing to Ischemia–Reperfusion Injury in Liver Transplantation
Source: Int J Genomics. 2026 Sep 21;2026:4835678. doi: 10.1155/ijog/4835678 (PMC13592132; doi:10.1155/ijog/4835678)
Supplement: Supplementary file 2 [file IJOG-2026-4835678-s019.zip › Supplementary_Enrichment_Results/olp.txt.anno.gene_GO_analysis/olp.txt.anno.gene_GO_enrichment_web_P.html]

GO enrichment analyze


# GO enrichment analyze result


| Term | Database | ID | Input number (44) | Background number (17574) | P-Value | Corrected P-Value | Input | Hyperlink |
| --- | --- | --- | --- | --- | --- | --- | --- | --- |
| protein localization | Gene Ontology | GO:0008104 | 6 (13.64%) | 145 (0.83%) | 1.55056813689e-06 | 2.48090901902e-05 | BAAT  UBE2D3  UBB  UBC  HAO2  BCL6 | web link |
| RNA splicing | Gene Ontology | GO:0008380 | 7 (15.91%) | 301 (1.71%) | 8.97596187466e-06 | 5.92062812442e-05 | SRSF7  SRSF3  SRRM2  DDX5  TXNL4B  MBNL2  HNRNPF | web link |
| cytokine-mediated signaling pathway | Gene Ontology | GO:0019221 | 7 (15.91%) | 311 (1.77%) | 1.11011777333e-05 | 5.92062812442e-05 | IL1RN  CXCL2  SAA1  IL32  UBB  UBC  BCL6 | web link |
| mRNA splicing, via spliceosome | Gene Ontology | GO:0000398 | 6 (13.64%) | 255 (1.45%) | 3.91279221734e-05 | 0.000139785658844 | SRSF7  SRSF3  SRRM2  DDX5  TXNL4B  HNRNPF | web link |
| mRNA processing | Gene Ontology | GO:0006397 | 7 (15.91%) | 385 (2.19%) | 4.36830183888e-05 | 0.000139785658844 | SRSF7  SRSF3  SRRM2  DDX5  MBNL2  TXNL4B  HNRNPF | web link |
| inflammatory response | Gene Ontology | GO:0006954 | 7 (15.91%) | 398 (2.26%) | 5.38966719443e-05 | 0.000143724458518 | IL1RN  CXCL2  PLD3  CDO1  CHI3L1  BCL6  ABCC2 | web link |
| translation | Gene Ontology | GO:0006412 | 5 (11.36%) | 311 (1.77%) | 0.00103682392217 | 0.00209014024403 | MRPL18  EIF3I  MRPL34  EIF4G1  EEF1D | web link |
| oxidation-reduction process | Gene Ontology | GO:0055114 | 6 (13.64%) | 469 (2.67%) | 0.00104507012202 | 0.00209014024403 | FMO3  CDO1  CYP2A7  FASN  HAO2  AKR1C1 | web link |
| transmembrane transport | Gene Ontology | GO:0055085 | 6 (13.64%) | 641 (3.65%) | 0.00498868473535 | 0.00886877286285 | UBB  SLC17A2  SLC22A7  SLC5A6  UBC  ABCC2 | web link |
| negative regulation of apoptotic process | Gene Ontology | GO:0043066 | 5 (11.36%) | 543 (3.09%) | 0.0111227719925 | 0.0177964351879 | RARA  BCL6  NFKBIA  UBB  UBC | web link |
| viral process | Gene Ontology | GO:0016032 | 5 (11.36%) | 596 (3.39%) | 0.0161011821645 | 0.02225896452 | KRT8  LTBR  EIF4G1  NFKBIA  ELOC | web link |
| lipid metabolic process | Gene Ontology | GO:0006629 | 5 (11.36%) | 607 (3.45%) | 0.0172953585348 | 0.02225896452 | BAAT  ST3GAL1  HDLBP  FASN  IL1RN | web link |
| protein ubiquitination | Gene Ontology | GO:0016567 | 5 (11.36%) | 614 (3.49%) | 0.0180854086725 | 0.02225896452 | ANAPC16  UBC  ELOC  UBB  UBE2D3 | web link |
| negative regulation of transcription by RNA polymerase II | Gene Ontology | GO:0000122 | 6 (13.64%) | 880 (5.01%) | 0.0215524159009 | 0.0246313324581 | RARA  UBE2D3  DDX5  UBB  UBC  BCL6 | web link |
| apoptotic process | Gene Ontology | GO:0006915 | 5 (11.36%) | 692 (3.94%) | 0.0285556247201 | 0.0304593330348 | LTBR  NFKBIA  UBE2D3  CHI3L1  GRAMD4 | web link |
| positive regulation of transcription by RNA polymerase II | Gene Ontology | GO:0045944 | 6 (13.64%) | 1149 (6.54%) | 0.0651072469475 | 0.0651072469475 | RARA  NCOA7  NFKBIA  APEX1  UBB  UBC | web link |
