# Supplementary material for: Comprehensive and Combined Omics Analysis Reveals Immune–Infiltration‐Associated Alternative Splicing and RBP Regulators Contributing to Ischemia–Reperfusion Injury in Liver Transplantation
Source: Int J Genomics. 2026 Sep 21;2026:4835678. doi: 10.1155/ijog/4835678 (PMC13592132; doi:10.1155/ijog/4835678)
Supplement: Supplementary file 2 [file IJOG-2026-4835678-s019.zip › Supplementary_Enrichment_Results/olp.txt.anno.gene_GO_analysis/Rplots.pdf]

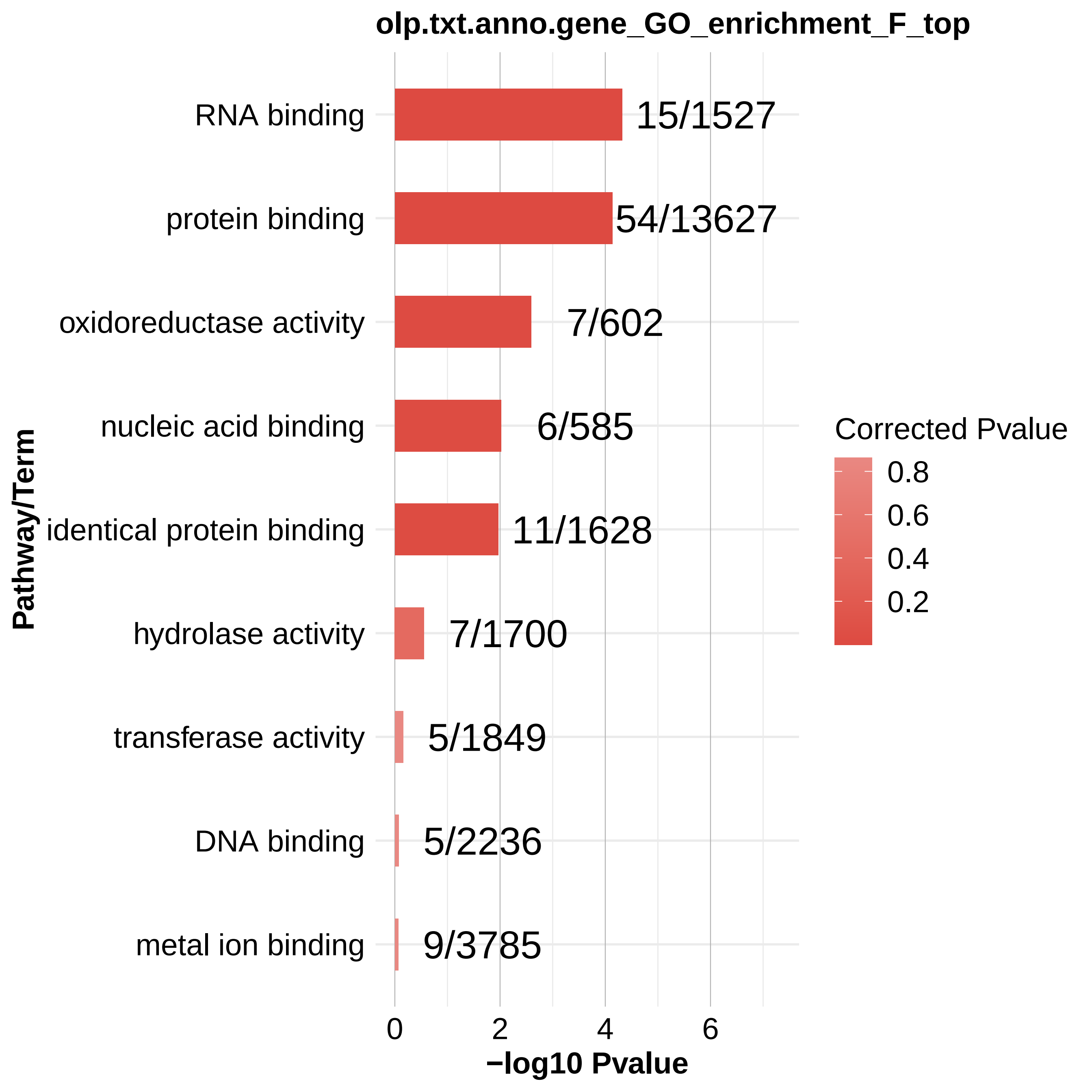

olp.txt.anno.gene_GO_enrichment_F_top
RNA binding
protein binding
oxidoreductase activity
nucleic acid binding
identical protein binding
hydrolase activity
transferase activity
DNA binding
metal ion binding
15/1527
54/13627
7/602
6/585
11/1628
7/1700
5/1849
5/2236
9/3785
Pathway/Term
0
2
4
6
−log10 Pvalue
Corrected Pvalue
0.8
0.6
0.4
0.2
