# Supplementary material for: Comprehensive and Combined Omics Analysis Reveals Immune–Infiltration‐Associated Alternative Splicing and RBP Regulators Contributing to Ischemia–Reperfusion Injury in Liver Transplantation
Source: Int J Genomics. 2026 Sep 21;2026:4835678. doi: 10.1155/ijog/4835678 (PMC13592132; doi:10.1155/ijog/4835678)
Supplement: Supplementary file 3 [file IJOG-2026-4835678-s015.doc]

**Supplementary Text S1. Complete Analytical Pipeline Pseudocode**

This document provides a step-by-step pseudocode description of the complete bioinformatics pipeline used in this study. All parameters and thresholds are derived directly from the Methods section of the main manuscript.

STEP 1: Data Acquisition and Quality Control

1.1 Download data from NCBI SRA

FOR each sample in GSE151648:

RUN prefetch to download .sra file

1.2 Convert SRA to FASTQ

FOR each .sra file:

RUN fastq-dump (NCBI SRA Tool) with:

- Split paired-end reads

- Output format = FASTQ

1.3 Quality trimming (FASTX-Toolkit v0.0.13)

FOR each FASTQ file:

RUN fastq_quality_filter with:

- Quality threshold = Q20

- Discard reads shorter than 36 bp

1.4 Quality assessment (FastQC)

FOR each trimmed FASTQ file:

RUN FastQC to generate quality report

IF per-base quality < Q20 OR GC content deviates > 3 SD:

FLAG sample for review

ELSE:

PROCEED to alignment

1.5 Quality control metrics

ASSESS final FPKM expression matrix:

- Mean genes detected per sample = 23,155 (range: 18,616–24,933)

- Verify consistency across four groups:

IRI− Pre, IRI− Post, IRI+ Pre, IRI+ Post

STEP 2: Read Alignment and Quantification

2.1 Build reference index (HISAT2)

BUILD index from human GRCh38 genome reference

2.2 Align reads to reference genome

FOR each trimmed FASTQ pair:

RUN HISAT2 with:

- index = GRCh38

- output = SAM file

2.3 Post-alignment processing

FOR each SAM file:

RUN samtools view -bS (convert to BAM)

RUN samtools sort (sort BAM file)

RUN samtools index (generate BAI index)

2.4 Quantify gene expression

FOR each sorted BAM file:

CALCULATE FPKM (fragments per kilobase of exon per million mapped fragments)

OUTPUT: Gene expression matrix (80 samples × genes)

STEP 3: Differential Expression Analysis (DESeq2)

3.1 Prepare input

READ raw read count matrix

CREATE DESeq2 dataset:

dds = DESeqDataSetFromMatrix(countData = counts,

colData = sample_metadata,

design = ~ patient + condition)

3.2 Run differential expression analysis

dds = DESeq(dds)

results = results(dds, contrast = c("condition", "Post", "Pre"))

3.3 Identify differentially expressed genes (DEGs)

FILTER results:

IF |log2FoldChange| >= 1 (i.e., |FC| >= 2) AND padj < 0.05:

MARK as significant DEG

ELSE:

MARK as non-significant

NOTE: padj = Benjamini-Hochberg-adjusted p-value

3.4 Export results

SAVE table: gene_id, log2FC, pvalue, padj

STEP 4: Alternative Splicing Analysis (SUVA Pipeline)

4.1 Run SUVA to detect alternative splicing (AS) events

FOR each sample (80 samples):

INPUT: sorted BAM file

RUN SUVA pipeline

CALCULATE pSAR (reads proportion of SUVA AS event) for each event

OUTPUT: Full list of AS events with pSAR values

4.2 Filter regulated alternative splicing (RAS) events for network construction

KEEP RAS events meeting BOTH criteria:

- frequency >= 0.2 (detected in >=20% of samples)

- pSAR >= 50% (major transcript isoform)

JUSTIFICATION:

- Frequency >= 0.2: retains events detected in at least 20% of

patient-paired comparisons within each IRI group, ensuring

recurrence across patients while preserving discovery sensitivity

- pSAR >= 50%: restricts analyses to major transcript isoforms,

which are more likely to have functional consequences

REFERENCE: Cheng et al., RNA Biology, 2021 (PMID: 34152934)

4.3 Identify highly conserved RAS events

SELECT RAS events meeting BOTH criteria:

- frequency >= 0.5

- pSAR >= 70%

LABEL as "highly conserved" for display in Figure 1G-H

4.4 Classify RAS events by type

ANNOTATE each RAS event as:

- alt5p (alternative 5' splice site)

- alt3p (alternative 3' splice site)

- ir (intron retention)

- olp (overlapping exons)

- MXE (mutually exclusive exons)

- A3SS (alternative 3' splice site)

- 5pMXE (mutually exclusive 5' UTRs)

- complex (involving multiple splice junctions)

4.5 Compare RAS between IRI− and IRI+ groups

PERFORM overlap analysis:

- RAS events overlapping between IRI− and IRI+ groups

- RAS host genes overlapping between groups

IDENTIFY group-specific RAS events

STEP 5: Immune Cell Deconvolution (CIBERSORT)

5.1 Prepare expression matrix

INPUT: FPKM expression matrix (80 samples × genes)

CONVERT to TPM values (required for CIBERSORT)

5.2 Run CIBERSORT (version 1.03)

FOR each sample:

RUN CIBERSORT with:

- Signature matrix = LM22 (22 human immune cell phenotypes)

- Parameters = default

- Permutations = 1000

OUTPUT: Immune cell fraction matrix (80 samples × 22 cell types)

5.3 Compare pre- vs post-transplant immune profiles

FOR each cell type:

PERFORM Student's t-test between Pre and Post groups

CALCULATE log2(Post/Pre) ratio

PERFORM separately for IRI− group (17 patients) and IRI+ group (23 patients)

MARK significance: * p ≤ 0.05; ** p ≤ 0.01; *** p < 0.001

STEP 6: RBP-RAS Co-expression Network Construction

6.1 Compile RBP catalog

MERGE RBPs from four published reports:

- Castello et al., Cell, 2012

- Castello et al., Molecular Cell, 2016

- Gerstberger et al., Nat Rev Genet, 2014

- Hentze et al., Nat Rev Mol Cell Biol, 2018

TOTAL: 2,142 unique human RBPs

6.2 Identify differentially expressed RBPs (DE RBPs)

FILTER expression data for RBP genes only

APPLY DESeq2 thresholds: |FC| >= 2, FDR < 0.05

TAKE union of DE RBPs from IRI− and IRI+ groups

OUTPUT: List of DE RBPs (43 RBPs identified in network)

6.3 Calculate RBP-RAS correlations

FOR each DE RBP:

FOR each RAS event (frequency >= 0.2, pSAR >= 50%):

CALCULATE Pearson correlation coefficient (PCC)

CALCULATE p-value

KEEP significant correlations:

IF |PCC| >= 0.6 AND p <= 0.01:

RETAIN edge

6.4 Calculate RAS-immune cell correlations

FOR each RAS event:

FOR each immune cell type:

CALCULATE Pearson correlation coefficient (PCC)

CALCULATE p-value

KEEP correlations:

IF |PCC| >= 0.5 AND p <= 0.01:

RETAIN edge (used in network, Figure 5B)

6.5 Build co-variation network

CREATE nodes:

- DE RBPs (triangles)

- RAS events (squares)

- Immune cells (rhombi)

ADD edges:

- RBP-RAS: |PCC| >= 0.6, p <= 0.01

- RAS-immune: |PCC| >= 0.5, p <= 0.01

Node size = degree of connection

VISUALIZE network (Figure 5B)

6.6 Threshold justification

- RBP-RAS |r| >= 0.6: strong effect size (Cohen's convention)

- RAS-immune |r| >= 0.5: moderate-to-strong effect size (Cohen's convention)

- Combined with p <= 0.01 (Benjamini-Hochberg adjusted)

REFERENCE: Cohen, Statistical Power Analysis for the Behavioral Sciences, 1988

STEP 7: Functional Enrichment Analysis

7.1 GO enrichment analysis (KOBAS 2.0)

FOR each gene list:

- RAS host genes (IRI− specific)

- RAS host genes (IRI+ specific)

- RAS host genes (overlapping)

- Host genes of 30 immune-associated RAS events

SUBMIT to KOBAS 2.0 server

USE hypergeometric test

APPLY Benjamini-Hochberg FDR correction

KEEP significant GO terms (FDR < 0.05)

7.2 KEGG pathway analysis (KOBAS 2.0)

FOR RAS host genes:

QUERY KEGG pathways

IDENTIFY enriched pathways (FDR < 0.05)

7.3 Reactome pathway analysis

FOR selected gene sets:

QUERY Reactome database (http://reactome.org)

IDENTIFY enriched pathways (FDR < 0.05)

STEP 8: Single-Cell RNA-Seq Analysis (Seurat v4.0.4)

8.1 Load data

DOWNLOAD UMI count matrix from GSE171539

CREATE Seurat object

8.2 Quality control and filtering

FILTER cells:

- nUMI >= 1,000

- nGenes >= 500

- mitochondrial UMI percentage <= 15%

FILTER genes:

- Detected in at least 3 cells

8.3 Normalization and scaling

APPLY LogNormalize (scale factor = 10,000)

SELECT top 2,000 variable genes (vst method)

SCALE all genes

8.4 Dimensionality reduction

RUN PCA on variable genes

RUN UMAP on first 30 principal components

8.5 Clustering (Louvain algorithm)

RUN FindNeighbors (dims = 1:30)

RUN FindClusters (resolution = 0.5)

8.6 Cell type annotation

FOR each cluster:

FIND marker genes (logFC >= 0.25, pct. >= 0.25)

ANNOTATE based on established cell type marker genes

Cell types identified:

C1: Granulocytes, C2: NK cells, C3: CD8+ T lymphocytes,

C4: Macrophages, C5: Endothelial cells, C12: Hepatocytes,

C14: Smooth muscle cells, C17: Macrophages, C19: Endothelial cells

NOTE: Seurat FindMarkers applies Benjamini-Hochberg correction by default,

with significance defined as p_val_adj < 0.05

8.7 Compare cluster proportions (Pre vs Post)

CALCULATE percentage of each cluster in Pre and Post samples

COMPUTE log2(Post/Pre) for each cluster

IDENTIFY significantly altered clusters

8.8 Visualize RBP expression

FOR each RBP (ZFP36, MBNL2, KRT18, ZC3H12A):

PLOT FeaturePlot on UMAP (Figure 6F-G)

PLOT VlnPlot across clusters (Figure 6H-I)

8.9 Differential expression per cluster

FOR each cluster:

RUN FindMarkers(ident.1 = "Post", ident.2 = "Pre")

PERFORM GO enrichment on up-regulated and down-regulated genes

per cluster (Figure 6D-E)

STEP 9: External Validation (GSE12720)

9.1 Load and preprocess microarray data

DOWNLOAD GSE12720 CEL files (20 paired pre-ischemia vs. post-reperfusion biopsies)

APPLY RMA normalization

MAP probes to gene symbols using hgu133plus2.db

9.2 Differential expression analysis (limma)

FOR each gene:

FIT linear model with paired design (donor + timepoint)

APPLY empirical Bayes moderation

COMPUTE log2 fold change and p-value

APPLY Benjamini-Hochberg correction

KEEP significant DEGs (FDR < 0.05)

9.3 Cross-reference with 43 RBPs

IDENTIFY which of 43 RBPs are detected on the array

FOR each detected RBP:

EXTRACT log2FC, FDR, direction of change

COMPARE with GSE151648 direction

9.4 Protein-level validation (Human Protein Atlas v23)

QUERY HPA database for liver tissue immunohistochemistry data

FOR each of 43 RBPs:

EXTRACT expression level (High/Medium/Low/Not detected)

NOTE cell type specificity (hepatocytes, cholangiocytes, etc.)

RECORD reliability score (Enhanced/Supported/Approved/Uncertain)

STEP 10: Visualization and Output

10.1 Principal Component Analysis (factoextra)

RUN PCA on RAS events

PLOT PCA with first two components (Figure S1D)

10.2 Clustering heatmap (pheatmap)

PERFORM hierarchical clustering based on Euclidean distance

GENERATE heatmaps for:

- RAS events (Figure 1G-H)

- DE RBPs (Figure 5A)

- HPA protein expression (Figure S7A)

10.3 Generate main figures

- Figure 1: RAS identification (scatter plots + heatmaps)

- Figure 2: RAS comparison (Venn + GO + boxplots + visualization)

- Figure 3: Immune cell profiles (boxplots + ratios)

- Figure 4: RAS-immune correlations (heatmap + GO + boxplots)

- Figure 5: RBP-RAS network (network + expression boxplots)

- Figure 6: Single-cell analysis (UMAP + proportions + RBP expression)

10.4 Generate supplementary figures

- Figure S1: PCA and additional RAS heatmaps

- Figure S2: KEGG pathway enrichment

- Figure S3: Complete immune cell fraction profiles

- Figure S4: Splicing ratio profiles of 30 immune-associated RAS events

- Figure S5: Additional RBP expression profiles

- Figure S6: Single-cell expression of KRT18 and ZC3H12A

- Figure S7: HPA protein expression validation

- Figure S8: GSE12720 external validation

Software and Versions Used

FASTX-Toolkit version 0.0.13 was used for quality trimming. FastQC was used for quality assessment. HISAT2 was used for read alignment to the GRCh38 reference genome. Samtools was used for BAM file processing and indexing. DESeq2 was used for differential expression analysis. SUVA version 1.0 was used for alternative splicing event detection and quantification. CIBERSORT version 1.03 was used for immune cell deconvolution. Seurat version 4.0.4 was used for single-cell RNA-seq data preprocessing, clustering, and visualization. KOBAS 2.0 was used for Gene Ontology and KEGG pathway enrichment analysis. Reactome was used for pathway profiling. Limma was used for differential expression analysis of the GSE12720 microarray dataset. R version 4.0.3 was used as the statistical computing environment. Factoextra was used for principal component analysis visualization. Pheatmap was used for hierarchical clustering heatmap generation.

Statistical Thresholds Summary

For differential expression analysis using DESeq2, genes with an absolute fold change of 2 or greater (|FC| ≥ 2, equivalent to |log2FC| ≥ 1) and a Benjamini-Hochberg-adjusted false discovery rate (FDR) below 0.05 were considered statistically significant differentially expressed genes. DESeq2 applies the Benjamini-Hochberg procedure internally, and significance was determined using padj (BH-adjusted p-value) < 0.05.

For alternative splicing analysis using the SUVA pipeline, regulated alternative splicing events (RAS) were filtered using two criteria. A frequency threshold of 0.2 was applied to retain events detected in at least 20% of patient-paired comparisons within each IRI group, ensuring recurrence across patients while preserving discovery sensitivity. A pSAR (reads proportion of SUVA AS event) threshold of 50% was applied to restrict analyses to major transcript isoforms, which are more likely to have functional consequences than minor isoforms. For highly conserved events, stricter thresholds of frequency ≥ 0.5 and pSAR ≥ 70% were applied. Sensitivity analyses evaluating alternative choices for both thresholds are provided in Table S10. No multiple testing correction was applied to the SUVA-based RAS event filtering, as frequency and pSAR are descriptive metrics of event recurrence and abundance rather than statistical test p-values.

For co-expression network construction, two distinct correlation thresholds were applied to reflect the different goals of each network layer. For RBP-RAS associations, a Pearson correlation coefficient of |r| ≥ 0.6 combined with p ≤ 0.01 was used to ensure that only strong-effect correlations entered network construction. For RAS-immune cell associations, a threshold of |r| ≥ 0.5 combined with p ≤ 0.01 was used to preserve sufficient network connectivity for visualization and biological interpretation. By Cohen's convention, |r| ≥ 0.6 and |r| ≥ 0.5 represent strong and moderate-to-strong effect sizes, respectively. Sensitivity analyses across alternative correlation thresholds confirmed that the chosen values are positioned at optimal inflection points in the retention curves (Table S10). For these exploratory co-expression network analyses, formal Benjamini-Hochberg FDR correction across all pairwise correlations was not applied, as it would be overly conservative for network discovery. Instead, we used a stringent nominal p-value threshold (p ≤ 0.01) in combination with a biological effect size filter (|r| ≥ 0.6 for RBP-RAS; |r| ≥ 0.5 for RAS-immune), consistent with the exploratory, hypothesis-generating nature of this network analysis. This approach prioritizes biologically meaningful correlations while controlling false positives through strict p-value and effect size cutoffs.

For functional enrichment analysis using KOBAS 2.0, the hypergeometric test was applied with Benjamini-Hochberg FDR correction, and enriched terms with FDR < 0.05 were considered statistically significant.

For single-cell RNA-seq analysis using Seurat version 4.0.4, quality control filtering excluded cells with unique molecular identifier (UMI) counts below 1,000, detected genes below 500, or mitochondrial UMI proportion exceeding 15%. Genes detected in fewer than three cells were also excluded. For marker gene identification, the FindMarkers function applies Benjamini-Hochberg correction by default, with significance defined as adjusted p-value (p_val_adj) < 0.05. Louvain clustering was performed at a resolution of 0.5 using the first 30 principal components.

For external validation in the GSE12720 microarray dataset, differential expression was performed using limma with a paired design. The Benjamini-Hochberg procedure was applied, and genes with FDR < 0.05 were considered statistically significant.

For comparisons between two groups in other statistical analyses, Student's t-test was used without multiple testing correction, with significance levels denoted as *p ≤ 0.05, **p ≤ 0.01, and ***p < 0.001.

Contact for Code Access

The custom analysis scripts used in this study are subject to institutional intellectual property protection and are integral to multiple ongoing research projects. Detailed pseudocode is provided above to ensure methodological transparency. Researchers interested in specific analytical details may contact the corresponding author for discussion.
